# Chalepin: isolated from *Ruta angustifolia* L. *Pers* induces mitochondrial mediated apoptosis in lung carcinoma cells

**DOI:** 10.1186/s12906-016-1368-6

**Published:** 2016-10-12

**Authors:** Jaime Stella Moses Richardson, Gautam Sethi, Guan Serm Lee, Sri Nurestri Abdul Malek

**Affiliations:** 1 Institute of Biological Sciences, Faculty of Science, University of Malaya, 50603 Kuala Lumpur, Malaysia; 2 Department of Pharmacology, Yong Loo Lin School of Medicine, National University of Singapore, Singapore, Singapore

**Keywords:** *Ruta angustifolia* L. *Pers*, Chalepin, Apoptosis, Mitochondrial mediated Apoptosis, Intrinsic pathway

## Abstract

**Background:**

Cancer has been one of the leading causes of mortality in this era. *Ruta angustifolia* L. *Pers* has been traditionally used as an abortifacient, antihelmintic, emmenagogue and ophthalmic. In Malaysia and Singapore, the local Chinese community used it for the treatment of cancer.

**Methods:**

In this study, the methanol and fractionated extracts (hexane, chloroform, ethyl acetate and water) of *R. angustifolia* were tested for its cytotoxicity using the sulforhodamide (SRB) cytotoxicity assay against HCT-116, A549, Ca Ski and MRC5 cell lines. Chemical isolation was carried out by using the high performance liquid chromatography (HPLC) and the isolated compounds were tested for its cytotoxicity against A549 cell line. Cellular and nuclear morphological changes were observed in the cells using phase contrast microscopy and Hoechst/PI fluorescent staining. The externalisation of phosphatidylserine was observed through FITC-labelling Annexin V/PI assay whilst DNA fragmentation was observed through the TUNEL assay. Other indication of apoptosis occuring through the mitochondrial pathway were the attenuation of mitochondrial membrane potential and increase in ROS production. Activation of caspase 9 and 3 were monitored. Western blot analysis was done to show the expression levels of apoptotic proteins.

**Results:**

The chloroform extract (without chlorophyll) exhibited the highest cytotoxic activity with IC_50_ of 10.1 ± 0.15 μg/ml against A549 cell line. Further chemical investigation was thus directed to this fraction which led to the isolation of 12 compounds identified as graveoline, psoralen, kokusaginine, methoxysalen, bergapten, arborinine, moskachan B, chalepin, moskachan D, chalepensin, rutamarin and neophytadiene. Among these compounds, chalepin exhibited excellent cytotoxicity against A549 cell line with an IC_50_ value of 8.69 ± 2.43 μg/ml (27.64 μM). In western blot analysis, expression of p53, truncated Bid, Bax and Bak while the anti-apoptotic proteins Bcl-2, survivin, XIAP, Bcl-X_L_,cFLIP decreased in a time-dependent manner when A549 cells were treated with 36 μg/ml of chalepin. In addition, the level of PARP was found to decrease.

**Conclusion:**

Hence these findings indicated that chalepin-induced cell death might involve the intrinsic mitochodrial pathway resulting in the upregulation of pro-apoptotic proteins and downregulation of anti-apoptotic proteins. Thus, chalepin could be an excellent candidate for the development of an anticancer agent.

**Electronic supplementary material:**

The online version of this article (doi:10.1186/s12906-016-1368-6) contains supplementary material, which is available to authorized users.

## Background

Lung cancer has the largest number of fatality and complications in comparison to other types of cancer. There are mainly two types of lung cancer i.e., the small cell lung cancer and the non-small cell lung cancer. According to the National Cancer Institute, US, tabacco smoking is the most common cause of lung cancer. In Malaysia, lung cancer is reported as the most common killer among malignancies with an estimated annual incidence of 30,000 [1]. It is noteworthy that, the current treatment for lung cancer does not cure the disease.

Cells undergoing apoptosis are characterised by cell shrinkage, blebbing of plasma membrane, and maintenance of organelle integrity, chromatin condensation and fragmentation of DNA, followed by programmed removal of dead cells by phagocytes. It is like a “suicide” program but does not cause any damage to the surrounding tissues. Apoptosis has been subclassified into two types of death pathways, namely, the extrinsic pathway and the mitochondria-mediated pathway [2]. All pathways of apoptosis converge upon the activation of caspases, which are a family of cysteine proteases that orchestrate the efficient and non-inflammatory demolition of cells. Two main pathways leading to caspase activation have been well characterized: the extrinsic route initiated by cell surface receptors leading directly to caspase 8 activation, and the intrinsic path that is regulated by the mitochondria [3].

Natural products has been a great success in our society with the usage of plant and microbial secondary metabolites in reducing ailments. The use of plant and microbial secondary metabolites has helped in doubling our life span in the 20th century. Since their chemical diversity is based on biological and geographical diversity, the entire globe is explored for bioprospecting by researchers [4].

*Ruta angustifolia* L*. Pers*. is a medicinal plant which originates from Southern Europe and North Africa. It grows up in the mountains to an elevation of 1,000 m above sea level. The plant often grows up to 1.5 m height with slender woody stems and light green leaves. The flowering period is normally between April to July. The plant has yellow flowers which gives off a very strong foetid odour. The plant reproduces via seeds and also grows from stem cuttings.

*Ruta angustifolia* is locally known as ‘garuda’ or ‘sadal’ in Malaysia, ‘inggu’, ‘godong minggu’ or ‘aruda’ in Indonesia, Rue in English and ‘luru’ in Vietnam. It is used for medicinal and culinary purposes since ancient times. *Ruta angustifolia* is not native to Southeast Asia but has been introduced here. The plant normally grows in mountainous areas i.e., about 1000 m above sea level. Besides that, it is also cultivated as a pot plant in Malaysia and occasionally in Vietnam and Java for medicinal purposes. The plant’s decoction is commonly used to cure cramps, flatulence and fever. In Indonesia, *R. angustifolia* has been known as traditional medicine for liver disease and jaundice. It has been reported to contain coumarin, alkaloid and flavonoid compounds [5]. The extracts of *Ruta angustifolia* (ethanol, hexane, dichloromethane and methanol) were recently reported to exhibit anti-viral activity. It exhibited anti-viral activity against hepatoma cell line (Huh7.5) with IC_50_ values ranging between 1.6 to 15.6 μg/ml [5]. Besides that, isolated compounds from the methanol extract of the roots and aerial parts of *Ruta graveolen*s which is another species of Ruta species, stimulated inhibition of platelet aggregation property and exhibited cytotoxicity against selected cancer cell lines such as colon, lung and cervical cancers [6]. These may suggest that the extracts of *R. angustifolia* may possess cytotoxicity. Other than that, extracts of leaves of *Ruta angustifolia* was found to be commonly used by the chinese community in Malaysia and Singapore in treatment of cancer (personal communication).

There are several earlier studies that has been reported for *Ruta angustifolia*. In 1986, Del Castillo [7] isolated angustifolin, a coumarin, from this plant and in 1966, a new shikimate metabolite was found from the aerial parts of *Ruta angustifolia*. These compounds were identified as moskachan A,B,C and D. In another study, chalepin and pseudane IX isolated from *Ruta angustifolia* was found to stop the replication of hepatitis C virus [5].

Up to date, there is no report on chalepin as a therapeutic agent for cancer. It is therefore of interest to study on the capability of chalepin to induce apoptosis.

## Methods

### Source of plant material

The whole plant of *Ruta angustifolia* L. *Pers* was obtained from a plant nursery near Sungai Buloh, Selangor, Malaysia. The plant was identified by Slamet Wahyono from the Research Station of Medicinal Plant and Traditional Medicine Research and Development Centre, Tawangmangu, Central Java, Indonesia. A voucher specimen numbered KLU48128 was deposited at the Herbarium of the Institute of Biological Sciences, Faculty of Science, University of Malaya on 26th April 2014.

### Preparation of plant extracts

#### Preparation of the methanol extracts and its fractionated extracts

The leaves of the plant were separated, washed and dried in an oven at a constant temperature of 50 °C for 3 days. The dried leaves were then ground using a commercial blender to a fine powder. The powdered leaves (175.0g) were soaked in methanol at room temperature yielding a greenish MeOH extract (55.0 g, 31.43 %). The MeOH extract (55.0 g) was further extracted with hexane to give a hexane soluble extract (2.96 g, 5.33 %) and a hexane insoluble residue. The hexane-insoluble residue was further partitioned between chloroform-water (100 mL: 100 mL) to give a chloroform extract (11.85 g, 21.35 %) and the aqueous layer was further partitioned with ethyl acetate-water (100 mL: 100 mL) to give the ethyl acetate extract (0.87g, 1.57 %) and H_2_O extract (30.08 g, 54.20 %). The crude MeOH and fractionated extracts (hexane, EtOAc and chloroform) were dissolved in dimethyl sulfoxide (DMSO) with the exception of the H_2_O extract which was dissolved in distilled water to form stock solutions 20 mg/mL before testing. The final concentration of DMSO in test wells was not in excess of 0.5 % (v/v).

#### Preparation of the chloroform extract without chlorophyll

Activated charcoal (1 teaspoon) was added to the chloroform extract (20 mg) in 50 ml methanol and the mixture was then mixed well. The mixture was immediately filtered with a filter paper (no. 1) to obtain the chloroform extract without chlorophyll.

### Cell culture

The human cell lines A549 (lung cancer) and Ca Ski (cervical cancer) were cultured as monolayer in RPMI 1640 growth media whereas HCT-116 (colon cancer) and MRC5 (human normal lung fibroblasts) was cultured in McCoy’s 5A and EMEM medium, respectively. All cells were purchased from the American Tissue Culture Collection (ATCC, USA). All the media were supplemented with 10 % v/v foetal bovine serum (FBS), 100 *μ*g/mL penicillin/streptomycin, and 50 *μ*g/mL amphotericin B. The cells were cultured in a 5 % CO_2_ incubator at 37 °C.

### In vitro cytotoxicity screening

The monolayer cell culture was detached using accutase and the cell count was adjusted to 2.5 x10^5^ -3.0 x10^5^ cells/mL using medium containing feotal bovine serum. The cells were then plated into 96 wells with a cell density of 5000-7000 cells/well and then incubated for 24 h to allow adherence of cells to the base of well of the plates. The media was then removed and 200 μl of fresh media containing different concentration of various extracts or isolated compounds were added. Cisplatin was used as the positive control. The plates were incubated for 24, 48 and 72 h at 37 °C and 5 % CO_2_. The cytotoxicity of the extracts and isolated compounds were screened using the Sulphoramide B (SRB) assay. This assay was first described by Houghton in 2007 [8]. The incubation was halted by gentle addition of 50 μl ice cold 4 % trichloroacetic acid (TCA) to each well and the plates were incubated at 4 °C for 1 h. Then, the supernatant was discarded and the plates were washed with distilled water for 5 times and then air dried. SRB dye, 50 μl, (0.4 % w/v) was added into each well and the treated cells and control were incubated at room temperature for 30 min. The unbound dye was removed by rapid washing four times with 1 % acetic acid. The plates were then air dried and 100 μl of tris base (10 mM unbuffered, pH 10.5) was added and the plates were shaken at 500 rpm for 5 min to solublize the bound SRB stain. The plates were then read with an ELISA reader (Synergy H1 Hybrid) at an absorbance of 492 nm.

### Isolation of compounds in the chloroform extract (treated with charcoal) using HPLC

The chloroform extract of *R. angustifolia* (treated with charcoal) was repeatedly subjected to HPLC separation. Treatment with charcoal was to remove chlorophyll that might interfere with the separation process.

Analytical HPLC analysis was initially performed on an Agilent 1260 infinity HPLC system consisting of a quaternary pump equipped with a 1260 autosampler (ALS), a 1290 thermostat, a 1260 thermostatted column compartment (TCC), a 1260 diode array detector (DAD VL+), a 1260 fraction collector (FC-AS) and Agilent OpenLAB CDS Chemstation for LC software. The analytical analysis was carried out using a binary eluent of chromatographic grade acetonitrile (ACN) and ultrapure water under the following gradient conditions: 0 to 20 min isocratic 30 % ACN; 20 to 25 min linear gradient from 30 to 60 % ACN; 25 to 35 min linear gradient from 60 to 100 % ACN; 35 to 40 min isocratic 100 % ACN at a flow rate of 1.0 ml/min. The column used was a ZORBAX Eclipse XDB-C18 (4.6 × 250 mm, 5 μm) and the temperature was set at 30 °C. The chloroform extract of *Ruta angustifolia* was filtered with activated charcoal to remove most of the chlorophyll and the filtrate was evaporated to dryness using a rotary evaporator. The extract was then prepared to a concentration of 5 mg/ml with methanol and filtered through a membrane filter (0.45 μm, Sartorius). The sample (5.0 μL) was injected onto the column and peaks were detected by monitoring the UV absorbance at 200 nm. Subsequently the sample was prepared at 50 mg/ml in methanol and then 100 μl of the sample was injected into the Agilent Semi Prep XDB-C18 column (9.4 × 250 mm, 5 μm) at a flow rate of 4.18 ml/min. Selected peaks in the resulting chromatogram were repeatedly collected using a fraction collector. Similar fractions from each round of separation were combined and the mobile phases were evaporated using a rotary evaporator at 40 °C, and the fractions were weighed.

From the HPLC analysis, twenty peaks were observed, and the eluents of the peaks were collected and then pooled to give twenty fractions (1-20) based on similarity of spots on TLC. Fractions that showed single spot on TLC were subjected to analytical HPLC analysis for determination of purity.

### Chemical characterization of compounds

All the compounds were identified through their mass spectral and NMR data. Some of the compounds were further confirmed by their Rf values from the TLC analysis by comparison with authenticated compounds. TLC precoated plates (silica gel 60F254) of 20-25 mm thickness were used and a solvent system consisting of chloroform with a few drops of methanol was used. Identification of compounds using gas chromatography-mass spectrometry (GCMS) was done using the Agilent Technologies 6980N gas chromatography equippped with a 5979 mass selective detector (70 eV direct inlet) was used. The column used was HP-5MS (5 % phenylmethyl siloxane) capillary column (30.0 mm × 25 mm × 25 μm) with helium as carrier gas at flow rate of 1mL min^-1^. The column temperature was programmed initially at 100 °C, then increased to 300 °C, at 3 °C per minute afterwhich the temperature was kept isothermally for 10 min. The compounds were identified by comparison of their mass spectral data with an accompanying mass spectral library NIST08 Spectral Library.

### Morphological studies

#### Phase contrast microscopy

A549 cells were seeded at a density of 5000-7000 cells into a tissue culture dish (6cm) and left overnight to adhere. Then, the cells were treated with chalepin at various concentrations for 24, 48, and 72 h at 37 °C and 5 % CO_2_. Changes in cytomorphology of the cells which include shrinkage, detachment, rounding, spiking, blebbing and formation of apoptotic bodies were observed using phase contrast microscopy (Zeiss Axio Vert. A1).

### Hoechst/PI fluorescent staining

This study was carried out to observe changes in cell nucleus. 5000-7000 cells were seeded into each 6 cm culture dish and allowed to adhere overnight. The cells were subsequently treated with various concentrations of chalepin for 48 and 72 h. At the end of the incubation period, the media in the culture dishes were discarded and the cells were washed twice with PBS, after which 1mL of PBS was added to each culture dish. To stain the cells with fluorescent dyes, 100 μ L of Hoechst solution (100μ g/mL) and 25 μL of PI solution (100 μ g/mL) were added to each culture dish and incubated for 15min. Finally, the cells were photographed using Leica DM-16000B fluorescent microscope.

### Apoptosis Detection by Annexin V-FITC/PI Staining

FITC annexin V apoptosis detection kit (BD) was used to detect apoptosis. Cells were seeded in tissue culture dishes (5000-7000 cells/dish) and allowed to adhere overnight. Next, the cells were treated with various concentrations of chalepin for 48 and 72 h. The cells were harvested, washed, and resuspended in 1x binding buffer according to the manual provided in the kit. In the next step, the cells were stained in the ratio of per 100,000 cells; staining of cells with 5 μL of FITC-annexin V and 5 μL of propidium iodide was done for 15 min. Then, 200 μL of 1× binding buffer was added to each sample. Unstained and single-stained untreated cells were also included as controls. A minimum number of 10,000 events was acquired for each replicate using Accuri C6 flow cytometer. A quadrant statistic was used to measure the population of viable, early apoptotic, late apoptotic and secondary necrotic cells. The determination betweent these different populations were obtained by estimating quantitatively the cells that were AnnexinV/PI stained.

### DNA fragmentation measurement by TUNEL assay

DNA strand breaks in apoptotic cells caused by the activation of endonucleases, were detected by APO-BrdU TUNEL assay kit. After incubation with various concentrations of chalepin at 48 and 72 h, cells were harvested and washed with PBS. Next, the cells were fixed with 1 % (w/v) paraformaldehyde for 15 mins, then washed with PBS, and fixed with 70 % (v/v) ethanol overnight. Ethanol was removed by centrifugation and DNA labeling steps were performed according to the manual provided in the kit. Samples were analyzed by flow cytometer and a minimum of 10,000 events were acquired for each replicate.

### Mitochondrial membrane potential assay

#### Flow cytometric analysis

A lipophilic and cell-permeable fluorochrome JC-1 (5,5’,6,6’-tetrachloro-1,1’,3,3’-tetraethyl benzimidazolyl carbocyanine iodide), was used to measure mitochondrial membrane potential (Δψm) according to the manufacturer’s instructions (BD MitoScreen Kit). The cells (1×10^6^) were seeded in tissue culture plates and were treated with 18, 27, 36, and 45 μg/ml of chalepin for 48 h incubation. The cells were then harvested, washed with PBS twice and incubated with JC-1 dye for 15 mins at 37°C. At the end of the incubation, the cells were washed and resuspended with 500 μl of 1x assay buffer (provided in the kit). The intracellular fluorescence signals of JC-1 in the cells were then measured with Accuri C6 flow cytometer. Approximately 10,000 events were recorded per analysis. In healthy cells, JC-1 accumulates as aggregates in the mitochondria and emits red fluorescence. In cells, which have undergone apoptosis, JC-1 remains in monomeric form in the cytoplasm and emits green fluorescence. The red and green fluorescence were detected at FL-2 and FL-1 channels respectively in the flow cytometer. Ratio of mean fluorescence intensity between the FL1 and FL2 channels were calculated and this is used to determine the changes in the mitochondrial membrane potential. The results were analysed by calculating the ratio of JC-1 dimers to JC-1 monomers. A higher ratio indicated a higher membrane depolarization of mitochondria in cells (BD MitoScreen Flow Cytometry Mitochondrial Membrane Potential Detection Kit Instruction Manual). Cells that was untreated in 0.5 % DMSO acted as the control.

#### Fluorescence microscope imaging

JC-1 (5,5’,6,6’-tetrachloro-1,1’,3,3’-tetraethyl benzimidazolyl carbocyanine iodide) mitochondrial membrane potential assay kit by Abcam was used to stain the A549 cells to view the mitochondrial membrane potential (Δψm) changes in cells. Prior to the JC-1 staining initiation, 1x dilution buffer were prepared by adding 10 mL 10× dilution buffer to 90 mL deionized water. The solution was mixed gently and throughly. JC-1 stock (1mM) was diluted to 20 μM working JC-1 solution. This was done by adding 200 μl of JC-1 stock solution into 10 mL of 1× dilution buffer. The cells (1×10^6^) were seeded in tissue culture plates and were treated with 18, 27, 36, and 45 μg/ml of chalepin for 48 and 72 h incubation. The cells were then harvested, washed with PBS twice and incubated with JC-1 dye for 20 mins at 37 °C. At the end of the incubation, the cells were washed and resuspended with 1000 μl of 1× dilution buffer. The cells were viewed using a fluorescence microscope at an excitation wavelength of Ex475 ± 20nm and emission wavelength of Em530 ± 15 nm and 590 ± 17.5 nm. The images were captured and recorded.

### Intracellular Reactive Oxygen Species (ROS) measurement

The assay was conducted as described by Ling et al. [9] with some minor modifications. The ROS measurement was done using the fluorescent probe 2’,7’-dichlorofluorescein diacetate (DCFH-DA) that enabled the monitoring of intracellular accumulation of ROS. The A549 cells were seeded into a 96 well plate with a density of 2×10^4^ cells per well and allowed to attach overnight. Treatment with chalepin at various concentrations were done the next day and incubated for different time periods. At the end of the incubation period, the media was removed and 5 μM of DCFH-DA which is diluted in media was added to the cells and incubated for 40 min at 37 °C. The cells were then washed three times with clear media and the fluorescence intensity (excitation = 485 nm and emission = 530 nm) was measured using a microplate reader. The morphology of the cells were observed using a fluorescence microscope.

### Caspase-3 and Caspase-9 Activity

Caspase activity assays were performed according to the instructions in the manual (CaspILLUME, Genetex). Cells were seeded at a density of 1×10^6^ cells per culture dish. After being subjected to treatment for 48 and 72 h, the cells were detached with accutase, washed, and resuspended in PBS. Next, 300 μL of each sample was aliquoted into centrifuge tubes, after which 1 μL of fluorescent substrate (FITC-DEVD-FMK for caspase 3 and FITC-LEHD-FMK for caspase 9 activity) was added to each tube and incubated for 1 h at 37°C incubator. At the end of the incubation period, the cells were centrifuged at 3000 rpm for 5 min and the supernatant was removed. After that, the cells were resuspended in 0.5 mL wash buffer (provided in the kit) and centrifuged at 3000 rpm for 5 min. This step was repeated before performing the analysis with flow cytometer (Accuri C6) and a minimum of 10,000 events were acquired for each replicate.

### Western blot analysis

For this analysis, 1× 10^5^ of A549 cells were plated per tissue culture dish. Chalepin treated whole-cell extracts were lysed in lysis buffer (20 mM Tris (pH 7.4), 250 mM NaCl, 2 mM EDTA (pH 8.0), 0.1 % Triton X-100, 0.01 mg/ml aprotinin, 0.005 mg/ml leupeptin, 0.4 mM PMSF, and 4 mM NaVO4). Lysates were then spun at 15,000 rpm for 10 min to remove insoluble material and resolved on a 10.0 % SDS-PAGE gel. After electrophoresis, the proteins were electrotransferred to a nitrocellulose membrane, blocked with Blocking One (Nacalai Tesque, Inc.), and probed with various antibodies (1:1000) overnight at 4 °C. The blot was washed, exposed to HRP-conjugated secondary antibodies for 1 h, and finally examined by chemiluminescence (ECL, Advansta).

### Statistical analysis

All the values reported are shown as mean ± standard error of the mean and all the experiments were conducted at least twice using sample triplicates. Figures from morphological studies, flow cytometry plots and Western blot analyses are representative of the experiment replicates. Comparison between control and treated groups were performed using one-way ANOVA with post hoc Tukey test (*p < 0.05 considered statistically significant). Statistical analysis were performed using SPSS 20.0 software and calculations were done using the Microsoft Excel. Quantification of the bands in the western blot analysis was done using the ImageJ software.

## Results

### Extraction, isolation of compounds and screening for cytotoxicity

Extraction and fractionation was done on the leaves of *R. angustifolia*. The yield of the methanol extract was 55.0 g which is 31.4 % of the total dried ground leaves. The methanol extract was further partitioned with solvents of different polarity. Water extract gave the highest yield followed by chloroform extract. Ethyl acetate extract had the lowest yield from methanol extract, as compared to the other fractions. The percentage of the fractionated extracts (hexane, chloroform, ethyl acetate and water) were calculated based on the methanol extract (Table 1).

**Table 1:** The yield of extracts of *R. angustifolia L. Pers* from 175.0 g of dried ground leaves

Extracts	Yield (g)	Percentage (%)
Methanol	55.0	-
Hexane	2.96	5.33
Chloroform	11.85	21.35
Ethyl Acetate	0.87	1.57
Water	30.08	54.20

### Cytotoxicity screenings of the crude and fractionated extracts of *R. angustifolia*

**Fig. 1:**
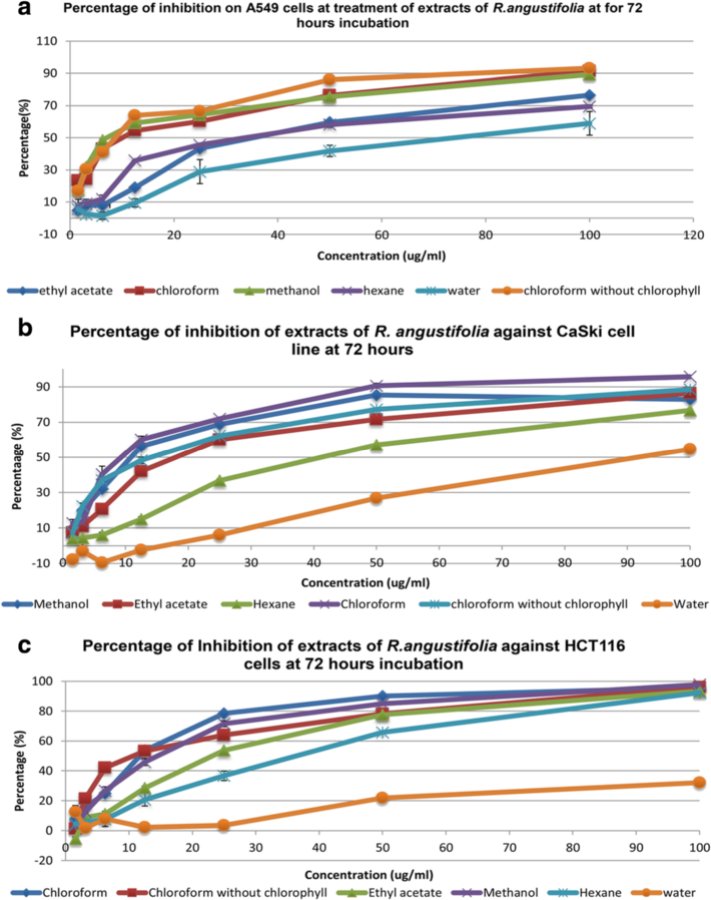
The percentage of inhibition of various extracts of *Ruta angustifolia* L. *Pers* against different cancer cell lines. **a** Graph shows the percentage of inhibition of various extracts of *Ruta angustifolia* L. *Pers* against A549 cell line at a range of concentrations in 72 h of incubation. **b** Graph shows the percentage of inhibition of various extracts of *Ruta angustifolia* L. *Pers* against Ca Ski cell line at a range of concentrations in 72 h of incubation. **c** Graph shows the percentage of inhibition of various extracts of *Ruta angustifolia* L. *Pers* against HCT 116 cell line at a range of concentrations in 72 h of incubation. The data expressed as mean ± S.D. of three independent replicates (*n* = 3)

Selected cancer cell lines (A549, Ca Ski and HCT-116) were used in the screening of the crude and fractionated extracts of *R. angustifolia* for their cytotoxic potency. Toxicity towards normal cells were tested against MRC5 cell line. All the extracts showed no toxicity towards MRC5 with an IC_50_ value of more than 100.00 μg/ml. The chloroform extract (without chlorophyll), showed slight toxicity with an IC_50_ value of 65.7 ± 0.5 μg/ml. It was found that the activity of all the extracts were time and concentration dependant in which the lowest IC_50_ value was observed when treatment was done over 72 h. The methanol extract exhibited good cytotoxic activity in all the cancer cell lines tested and without toxicity to normal cells. The chloroform extract showed the highest activity and the cytotoxicity was even better when chlorophyll was removed; however it exhibited slight toxicity to MRC5 cell. The chloroform extract without chlorophyll exhibited the best cytotoxicity in A549 cell line with an IC_50_ of 8.8 μg/ml; hence it was chosen as a candidate for further isolation of the chemical components (Table 2) (Fig. 1).

**Table 2:** In vitro cytotoxicity screening with SRB assay upon various extracts of *Ruta angustifolia* L. *Pers* against selected cell lines

Cell Line		A549	CasKi	HCT-116	MRC5
Extract	Incubation time	IC_50_ (μg/ml)			
Methanol	72	7.02 ± 0.36	10.56 ± 0.09	14.3 ± 0.93	>100
	48	51.2 ± 1.85	35.00 ± 1.13	49.9 ± 0.36	>100
	24	85.60 ± 0.85	>100.0	14.3 ± 0.93	>100.0
Hexane	72	33.1 ± 1.667	41.20 ± 0.23	49.9 ± 0.36	>100.0
	48	>100.0	>100.0	>100.0	>100.0
	24	>100.0	>100.0	36.40 ± 2.01	>100.0
Chloroform	72	10.1 ± 0.35	9.38 ± 0.49	88.0 ± 2.65	>100.0
	48	22.67 ± 4.31	29.00 ± 0.46	>100.0	>100.0
	24	72.0 ± 0.5	>100.0	11.8 ± 0.1	>100.0
Ethyl Acetate	72	35.1 ± 1.00	18.10 ± 0.15	12.40 ± 0.85	>100.0
	48	>100.0	46.00 ± 0.76	27.1 ± 0.21	>100.0
	24	>100.0	>100.0	23.2 ± 0.42	>100.0
Water	72	73.9 ± 8.30	92.2 ± 2.58	>100.0	>100.0
	48	>100	>100.0	*>*100.0	>100.0
	24	>100	>100.0	>100.0	>100.0
Chloroform without chlorophyll	72	8.8 ± 0.32	13.9 ± 1.23	10.8 ± 0.7	65.7 ± 0.5
	48	23.20 ± 0.90	22.1 ± 0.21	14.3 ± 0.45	>100.0
	24	88.0 ± 0.5	>100.0	21.9 ± 0.46	>100.0

Tabulated values are mean ± standard deviation (SD) of three replicates

### Chemical isolation of active compounds from the chloroform extract (without chlorophyll) of *R. angustifolia*

**Fig. 2:**
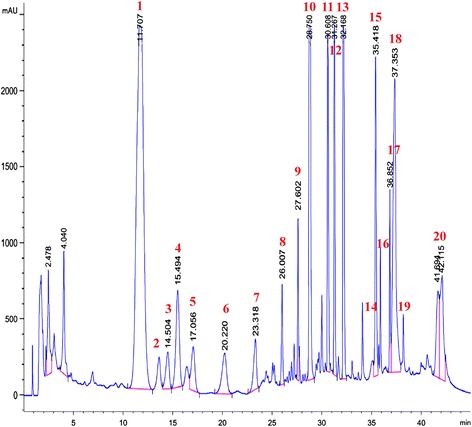
High Performance Liquid Chromatography (HPLC) chromatogram of the chloroform extract (without chlorophyll) of *Ruta angustifolia* L. *Pers*

**Fig. 3:**
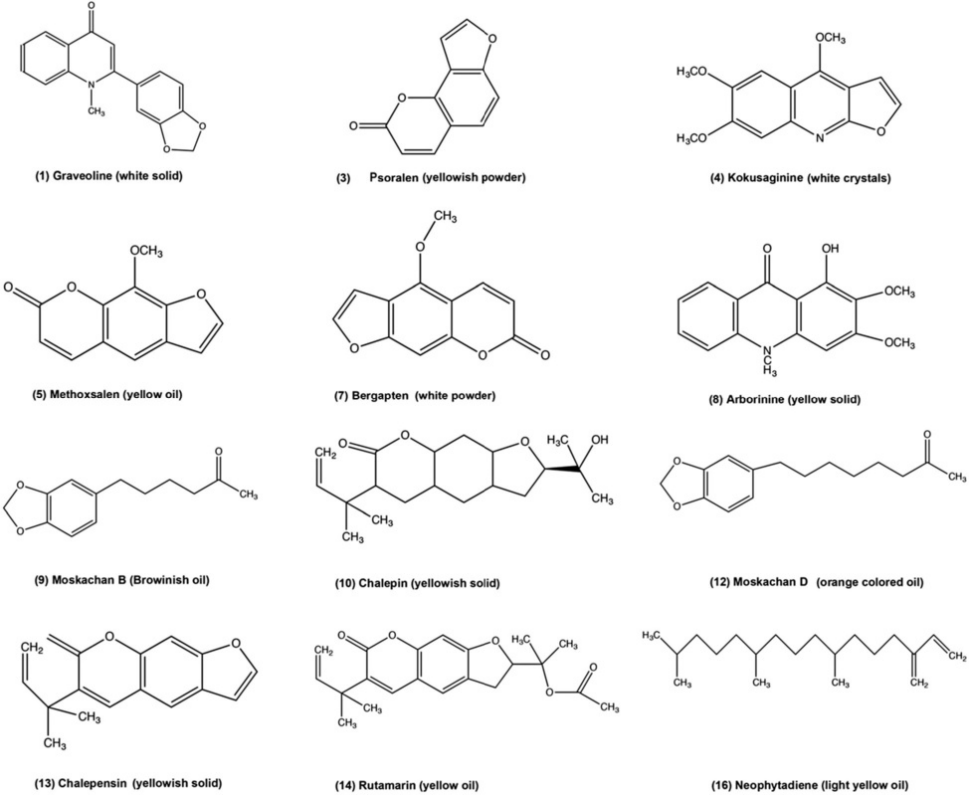
Structures of isolated compounds

The chloroform extract was subjected to isolation and purification using HPLC. Fractions were repeatedly collected using preparative HPLC method. The HPLC profile of the chloroform extract was shown in Fig. 2. Twenty fractions were collected and twelve compounds were successfully identified using GCMS analyses. The mass spectral data of the isolated compounds obtained were found to be consistent with previous reports [6, 7, 10–12]. The compounds which has been successfully identified are graveoline (1), psoralen (3), kokusaginine (4), methoxsalen (5), bergapten (7), arborinine (8), moskachan B (9), chalepin (10), moskachan D (12), chalepensin (13), rutamarin (14) and neophytadiene (16). The structures of these compounds are illustrated in Fig. 3.

### Identification of chemical components

**Fig. 4:**
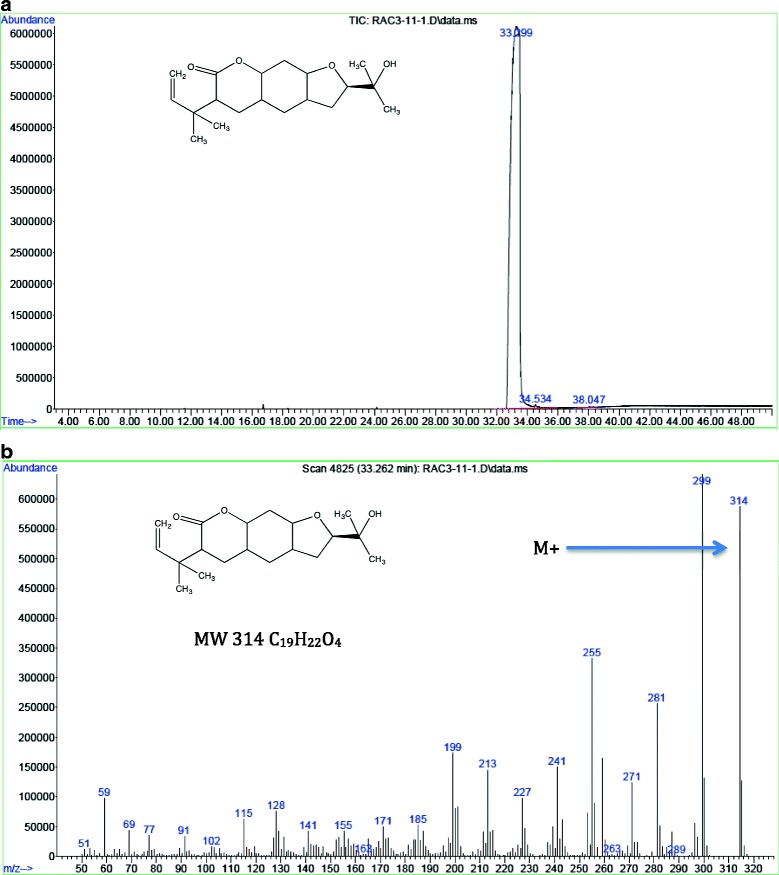
GCMS analysis of Chalepin (**a**) Total ion chromatogram (**b**) Mass spectrum

**Fig. 5:**
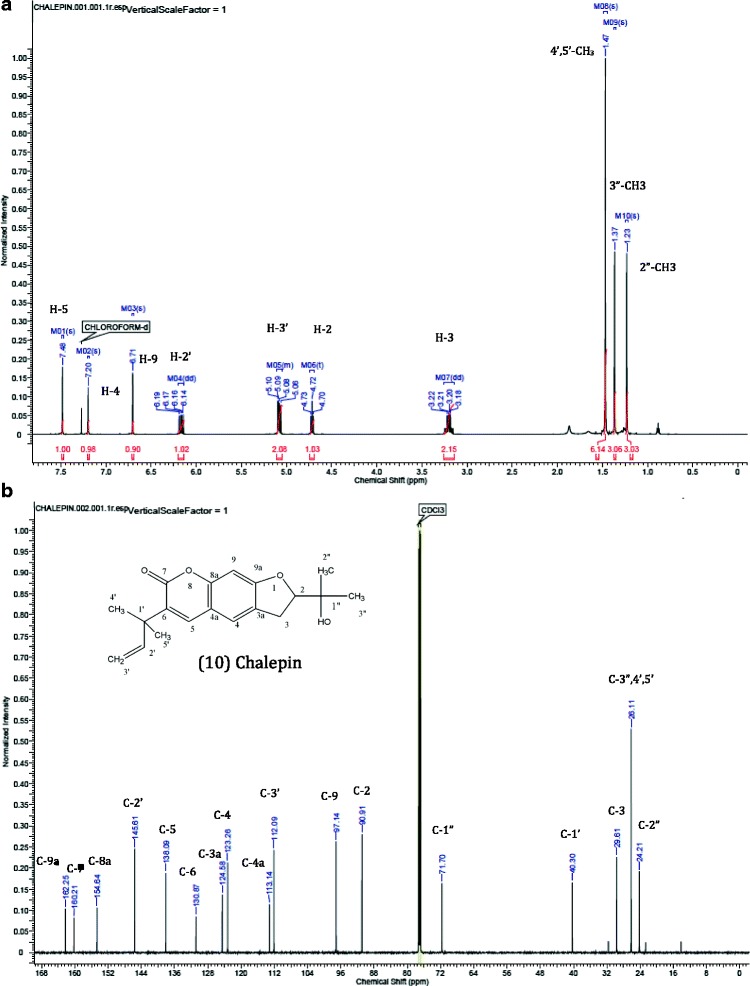
NMR spectra of isolated chalepin. **a** Proton NMR spectra and **b** ^13^C NMR spectra

The compound graveoline, psoralen, kokusaginine, methoxsalen, bergapten, arborinine, moskachan B, moskachan D, chalepensin, rutamarin and neophytadiene were identified through their mass spectral data which were found to be consistent with published data [6, 7, 10–12]. However chalepin was identified through its’ mass spectral which were consistent with published data [12] and NMR data. Some collected peaks of HPLC were not identified due to insufficient amount. The following compounds analysed were identified from their EI mass spectra by their m/z values: *Graveoline,* colorless powder, EIMS m/z (%), 279 ([M]^+^, 100), 251 ([M-CO]^+^, 92), 192 (22), 125 (25). The mass spectral data obtained was in agreement with published data [6]. *Psoralen*, yellowish powder, EIMS m/z (%), 186 ([M]^+^, 100), 158 ([M-CO]^+^, 65), 130 ([M-CO-CO]^+^, 19) and 102 ([M-CO-CO-CO]^+﻿^, 32). The mass spectral data was in agreement with published data of psoralen [6, 11]. *Kokusaginine*, white crystals, EIMS m/z (%), 259 ([M]^+^, 100), 244 ([M-Me]^+^, 43), 216 ([M-Me-CO]^+^, 15), 201 ([M-Me-CO-Me]^+^, 19), 186 ([201-Me]^+^, 25) and 173 ([186-CH]^+^, 16). The obtained mass spectral data is in agreement with published data [6]. *Methoxsalen*, yellowish oil, EIMS m/z (%), 216 ([M]^+^,100), 201 ([M-Me]^+^, 35), 188 ([M-CO]^+^,14), 173 ([188-CO]^+^, 54) and 145 ([173-CO]^+^, 21). The data obtained was consistent with the mass spectral data of methoxsalen from NIST webbook. *Bergapten*, white powder, EIMS m/z (%), 216 ([M]^+^, 100), 201 ([M-Me]^+^, 35), 188 ([M-CO]^+^, 15), 173 ([M-Me-CO]^+^, 64) and 145 ([M-Me-CO-CO)]^+^, 21). The data obtained was consistent with published data [12]. *Arborinine*, yellow crystal, EIMS m/z (%), 285 ([M]^+^, 66), 270 ([M-Me]^+^, 100), 242 ([M-Me-CO]^+^, 49). The mass spectra was in agreement with published data [11]. *Moskachan B*, brownish oil, EIMS m/z (%), 220 ([M]^+^, 21), 135 ([M-CH_2_CH_2_CH_2_COCH_3_]^+^, 100). The mass spectra was in agreement with published data [7]. *Chalepin*, white crystals, EIMS m/z (%), 314 ([M]^+^, 88), 299 ([M-Me]^+^, 100), 281 ([M-Me-H_2_O]^+^, 34) and 255 ([M-(CH_3_)_2_COH]^+^. The mass spectral data in the present study corresponds to the data reported in published data [12]. The GCMS analysis of chalepin is shown in Fig. 4. ^1^H NMR data (600 MHz, CDCl_3_): δ 7.48 (1H, s, H-5), δ 7.20 (1H, s, H-4), δ 6.71 (1H, s, H-9), δ 6.17 (1H, dd, J = 18.00, 12.00 Hz, H-2’), δ 5.09 (2H, overlapping dd, H-3’), δ 4.72 (1H, dd, J = 12.00, 6.00 Hz, H-2), δ 3.21 (2H, overlapping dd, J = 18.00, 12.00, 6.00 Hz, H-3), δ 1.47 (6H, s, 4’,5’-CH_3_), δ 1.37 (3H, s, 3”-CH_3_), δ 1.23 (3H, s, 2”-CH_3_). The ^13^C NMR data (600 MHz, CDCl3): δ 162.25 (C-9a), δ 160.21 (C-7), δ 154.64 (C-8a), δ 145.61 (C-2’), δ 138.09 (C-5), δ 130.87 (C-6), δ 124.58 (C-3a), δ 123.26 (C-4), δ 113.14 (C-4a), δ 112.09 (C-3’), δ 97.14 (C-9), δ 90.91 (C-2), δ 71.70 (C-1”), δ 40.30 (C-1’), δ 29.61 (C-3), δ 26.11 (C-3”,4’,5’), δ 24.21 (C-2”). The NMR spectra of isolated chalepin is shown in Fig. 5. *Moskachan D*, yellowish oil, EIMS m/z (%), 248 ([M]^+^, 30), 148 ([M-CH_2_-CH_2_-CH_2_-CH_2_-COCH_3_]^+^, 15), 135 ([M- CH_2_-CH_2_-CH_2_-CH_2_-COCH_3_-CH]^+^,100),91([M-CH_2_-CH_2_-CH_2_-CH_2_-COCH_3_-CH-COO]^+^,3), 77([C_6_H_5_]^+^, 11). The obtained data is consistent with published data [7]. *Chalepensin*, yellowish solid, EIMS m/z (%), 254 ([M]^+^, 100), 239 ([M-CH_3_]^+^, 95), 211 ([M-CH_3_-CH-CH_3_]^+^, 70), 199 ([M-CH_3_-CH-CH3-C]^+^, 85). The mass spectra is in agreement with published data [6]. *Rutamarin*, colorless crystals, EIMS m/z (%), 356 ([M]^+^, 8), 341 ([M-CH_3_]^+^, 4), 296 ([M-CH_3_-COO]^+^, 19), 281 ([M-CH_3_-COO-CH_3_]^+^, 100), 253 ([M-CH_3_-COO-CH_3_-CO]^+^, 14). The mass spectral data is in agreement with published data [6]. *Neophytadiene*, light yellow oil, EIMS m/z (%), 278 ([M]+, 15), 137 (14), 123 (68), 109 (42), 95 (71), 82 (70) and 71 (100). The mass spectral from GCMS analysis is identified through comparison with NIST MS Library data (Table 3).

**Table 3:** Identified isolated chemical compounds using GC-MS

Peak No.	Compounds	Molecular weight	Molecular formula	Retention time (s)
1	Graveoline	279	C_17_H_13_NO_3_	39.22
2	Unidentified	-	-	-
3	Psoralen	186	C_11_H_6_O_3_	18.46
4	Kokusaginine	259	C_14_H_13_NO_4_	29.98
5	Methoxsalen	216	C_12_H_8_O_4_	22.54
6	Unidentified	-	-	-
7	Bergapten	216	C_12_H_8_O_4_	23.06
8	Arborinine	285	C_16_H_15_NO_4_	37.13
9	Moskachan B	220	C_13_H_16_O_3_	18.35
10	Chalepin	314	C_19_H_22_O_4_	33.26
11	Unidentified	-	-	-
12	Moskachan D	248	C_15_H_20_O_3_	22.65
13	Chalepensin	254	C_16_H_14_O_3_	25.65
14	Rutamarin	356	C_21_H_24_O_5_	34.72
15	Unidentified	-	-	-
16	Neophytadiene	278	C_20_H_38_	23.85
17	Unidentified	-	-	-
18	Mixture of acids, Stigmasterol and Vitamin E	-	-	-
19	9,12,15-octadecatrienoic acid, Vitamin E, γ-sitosterol	-	-	-
20	12-docosenamide	-	-	-

The isolated compounds were tested for their cytotoxicity effect against A549 human lung carcinoma cells and the normal human lung fibroblasts MRC5 cells. Results showed that the cytotoxicity effect against A549 cell was dose and time dependent. Chalepin exhibited the highest cytotoxicity against A549 cell line after 72 h incubation with a IC_50_ value of 8.69 ± 2.43 μg/ml. It was also mildly toxic to the normal cell line with a IC_50_ value of 23.4 ± 0.6 μg/ml. Arborinine, chalepensin and also moskachan D also exhibited promising cytotoxic property against A549 cell line with IC_50_ values of 13.1 ± 0.06, 14.0 ± 0.15 and 18.5 ± 0.65 μg/ml respectively. However, arborinine and chalepensin showed moderate toxicity towards MRC5 normal human lung fibroblast cell line with IC_50_ values of 20.8 ± 0.15 and 23.3 ± 0.55 μg/ml respectively. Moskachan D was non-toxic towards normal cell with an IC_50_ value of 90.8 ± 0.8 μg/ml. Other isolated compounds showed no toxicity towards the normal cells with IC_50_ values ranging more than 50.0 μg/ml. The results are represented in Table 4.

**Table 4:** IC_50_ value of isolated compounds against A549 cell lines at 48 and 72 h incubation and MRC5 cell line at 72 h treatment

Isolated Compounds	IC_50_ in μg/ml (μM)		
	(A549)		(MRC5)
	48 h	72 h	72 h
Graveoline	76.3 ± 2.08 (273.5)	44.6 ± 0.47 (159.9)	69.7 ± 2.5 (249.8)
Kokusaginine	87.4 ± 7.08 (337.5)	>100 (>386.1)	74.8 ± 0.82 (288.8)
Bergapten	>100 (>463.0)	43.53 ± 1.81 (201.5)	>100 (>463.0)
Moskachan B	>100 (>454.5)	>100 (>454.5)	>100 (>454.5)
Moskachan D	77.5 ± 3.0 (312.5)	18.5 ± 0.65 (74.6)	90.8 ± 0.8 (366.1)
Chalepensin	30.5 ± 1.30 (120.1)	14.0 ± 0.15 (55.1)	23.3 ± 0.55 (91.7)
Rutamarin	56.3 ± 1.53 (158.1)	68.5 ± 3.06 (192.4)	>100 (>281.0)
Arborinine	27.7 ± 0.26 (97.2)	13.1 ± 0.06 (46.0)	20.8 ± 0.15 (73.0)
Chalepin	28.3 ± 1.06 (90.1)	8.69 ± 2.43 (28.0)	23.4 ± 0.6 (74.5)
Neophytadiene	69.2 ± 1.06 (248.9)	68.00 ± 2.43 (244.6)	77.4 ± 0.98 (278.4)
Cisplatin^a^	-	24.5 ± 1.8 (81.67)	>100 (>333.33)

^a^Cisplatin was used as the positive standard reference. Tabulated values are mean ± standard deviation (SD) of three replicates

### Morphological changes induced by chalepin

#### Phase contrast microscopy studies

**Fig. 6:**
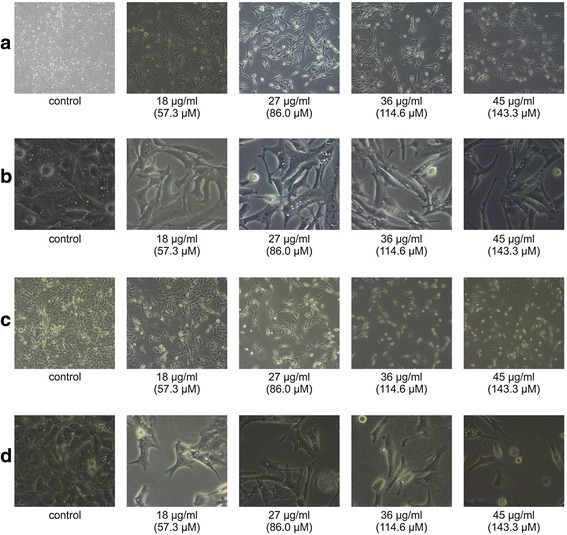
Untreated control was compared with A549 cells treated at different doses and time. The morphological changes in the cells were observed under phase-contrast microscopy at 10x and 40x magnification. **a** A549 cells treated with different concentrations of chalepin for 48 h and observed at 10x magnification. **b** A549 cells treated with different concentrations of chalepin for 48 h and observed at 40x magnification. **c** A549 cells treated with different concentrations of chalepin 72 h and observed at 10x magnification. **d** A549 cells treated with different concentrations of chalepin for 72 h and observed at 40x magnification. Cells shrinkage, formation of echinoid spikes, vacuolisation, formation of apoptotic bodies and rounding were observed clearly

Morphological changes in the A549 cells treated with different concentrations of chalepin were observed using the inverted phase contrast microscope. Typical morphological features of apoptosis such as plasma membrane blebbing, cell vacuolisation, echinoid spiking, chromatin condensation, formation of apoptotic bodies, cell shrinkage, nuclear fragmentation and others were observed. Untreated cells appeared to be thriving healthily in the culture. After 48 h of incubation, formation of apoptotic bodies and cell vacuolisation were observed whereas after 72 h, most of the cells were floating and there was a shrinkage and also decrease in the cell number. At 40× magnification, alteration in condensation of chromatin and in shape of cells were observed (Fig. 6).

### Hoesct 33342 and PI nuclear staining

**Fig. 7:**
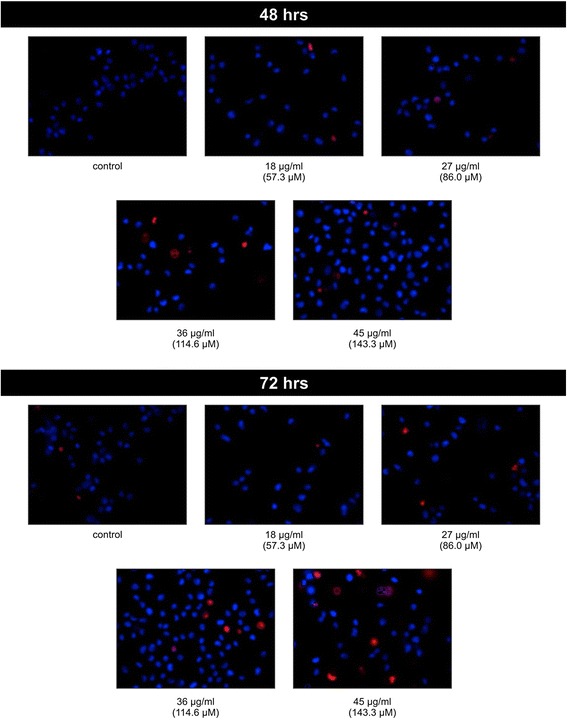
Effects of chalepin at different concentrations on nuclear morphological changes of A549 cell line at 48 h and 72 h incubation. After the incubation period, the cells were stained with Hoechst 33342 and PI and were examined with a fluorescent microscope under magnification of 400x to observe the chromatin condensation, nuclear fragmentation and cell shrinkage

The nuclear morphological changes after treatment with chalepin at various concentrations were observed using Hoesct 33342 and propidium iodide (PI) dye, and was observed under a fluorescent microscope. The control showed only low blue color. After treatment of chalepin for 72 h at various concentrations, observation of cells fluorescing bright blue colored nucleus was observed. The higher the concentration of chalepin, the higher the number of cells that emits a bright blue signal. In 40x magnification, chromatin condensation and nuclear cleavage were observed (Fig. 7).

### Annexin V-FITC/PI apoptotic analysis

**Fig. 8:**
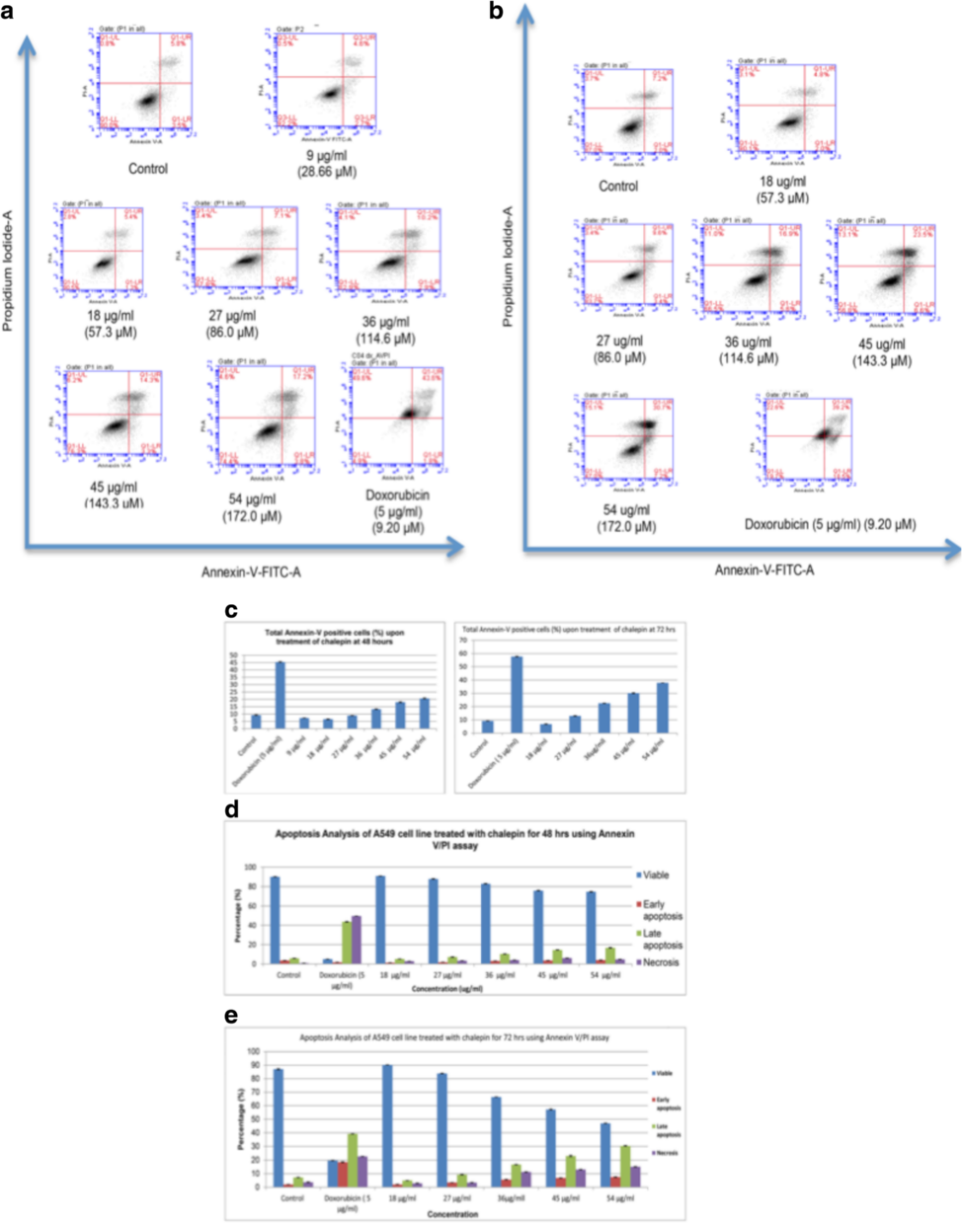
Induction of early and late apoptosis by chalepin at various concentrations in A549 cell line. **a** shows the flow cytometric density plot of Annexin V-FITC/PI staining in A549 cells when treated with different concentrations of chalepin (18.0-45.0 μg/ml) at 48 h incubation. **b** Shows the flow cytometric density plot of Annexin V-FITC/PI staining in A549 cells when treated with different concentrations (18.0 - 45.0 μg/ml) at 72 h incubation time. **c** The bar chart shows the total annexin V positive cells. **d** The bar charts shows the distribution of cells in viable, early apoptosis, late apoptosis and secondary necrosis phase in 48 h incubation. **e** The bar charts shows the percentage of distribution of the treated cell population in viable, early apoptosis, late apoptosis or secondary necrosis phase in 72 h incubation. Doxorubicin served as the positive control. The data is expressed as mean ± S.D. from three replicates. Asterisks indicates significantly different value as compared to control (*p < 0.05)

The phosphatidylserine (PS) exposure on the outer leaflet of the plasma membrane is a hallmark of apoptosis. Annexin V is a protein which has high affinity for PS. Thus the binding of Annexin V to PS provides a very sentitive method for detecting cellular apoptosis. A population of cells undergoing apoptosis may contain necrotic cells due to their damaged plasma membrane. To distinguish between Annexin V positive apoptotic and necrotic cells, the fluorescent dye propidium iodide was used. Membranes of damaged cells are permeable to propidium iodide. Thus, using Annexin V conjugated to FITC (a fluorescent dye) enables apoptotic cells to be identified and quantified on single cell basis by flow cytometry. In this study, it can be observed that as the treatment concentration of chalepin increased, the density plot showed increase in the cell population at the lower right quadrant (Q1-LR) which represents the early apoptotic cells and also subsequently towards the upper right quadrant (Q1-UR) which represents population of late apoptotic/secondary necrotic cells. Comparison between 48 h and 72 h showed that at longer incubation, the cell population in early, late apoptosis/secondary necrosis (upper right quadrant – Q1-UL) increased with dose. But the highest increase was observed in the late apoptosis quadrant represented by upper right quadrant in the density plot. The untreated, control cells which stayed at lower left quadrant (Q1-LL) showed low staining with both annexin V and PI showing viable cells. The total AnnexinV positive cells represents a total of early and late apoptotic cells in A549 cells, shows an increase in dose and time dependant manner. The Annexin V positive cells showed an increase from 7.27 ± 0.16 % at 9 μg/ml to 20.6 ± 0.53 % at 45 μg/ml of chalepin. The population of AnnexinV positive cells observed was significantly higher after 72 h incubation. These results are﻿ shown in Fig. 8.

### DNA fragmentation induced by chalepin on A549 cell

**Fig. 9:**
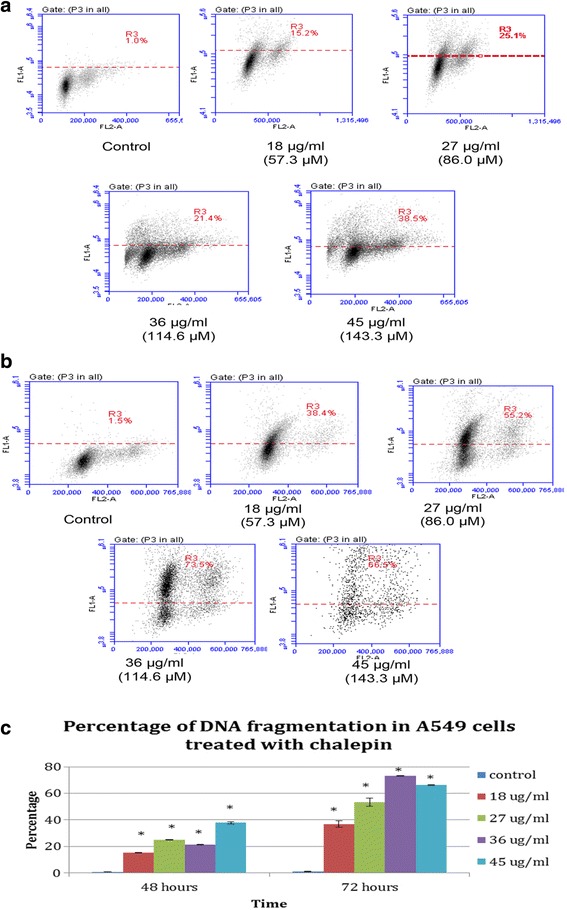
DNA fragmentation based on TUNEL assay on A549 cells treated with chalepin at various concentrations at 48 h and 72 h incubation. **a** R3 region in the dot plots obtained from flow cytometry represents TUNEL positive staining in which cells were stained with FITC-conjugated anti-BrdU antibody which resulted from treatment of chalepin on A549 cells at 48 h incubation. **b** R3 region in the dot plots obtained from flow cytometry represents TUNEL positive staining in which cells were stained with FITC-conjugated anti-BrdU antibody resulted from treatment of chalepin on A549 cells at 72 h incubation. **c** Bar chart represents the percentage of TUNEL positive cells. Histograms are representative of three separate experiments (*n* = 3). Asterisks indicate significantly different value from control (*p < 0.05)

DNA fragmentation is one of the main distinctive feature of apoptosis. DNA fragmentation is activated by nucleases which would degrade the nuclear DNA into fragments of about 200 base pairs in length. This feature is measured by using APO-BrDU TUNEL assay kit. In this study, chalepin was treated at various concentrations and incubated for 48 and 72 h to measure the ability to induce DNA fragmentation. It was found that chalepin induces DNA fragmentation in A549 cell in a dose and time dependent manner (Fig. 9). DNA fragmentation is represented in the density plot above with R3 representing percentage of cells that has nuclear DNA fragmentation for treatment at 48 h and 72 h. The cell population would move to the upper region in the density plot when DNA fragmentation is labelled. Upper region in the density plot represents cells with fragmented DNA. Based on the results obtained, after 48 h incubation, the DNA fragmentation increased from 1.0 ± 0.08 % for untreated control cells to 38.5 ± 0.70 % for cells treated with chalepin at 45 μg/ml. At 72 h, the DNA fragmentation increased from 1.50 ± 0.35 % for untreated control cells to 73.50 ± 0.20 % at 36 μg/ml however a drop was observed at 45 μg/ml to 66.50 ± 0.29 %. This result indicates that upon treatment of chalepin, DNA fragmentation is initiated in the cell by endonucleases to enable cells to undergo apoptosis. With increasing concentration of treated chalepin, the percentage of DNA fragmentation in the treated cells increases. This trend is also true for higher incubation time. The DNA fragmentation that occurs in the cells is dependant on the concentration of chalepin and the incubation hours.

### Measurement of mitochondrial membrane potential

**Fig. 10:**
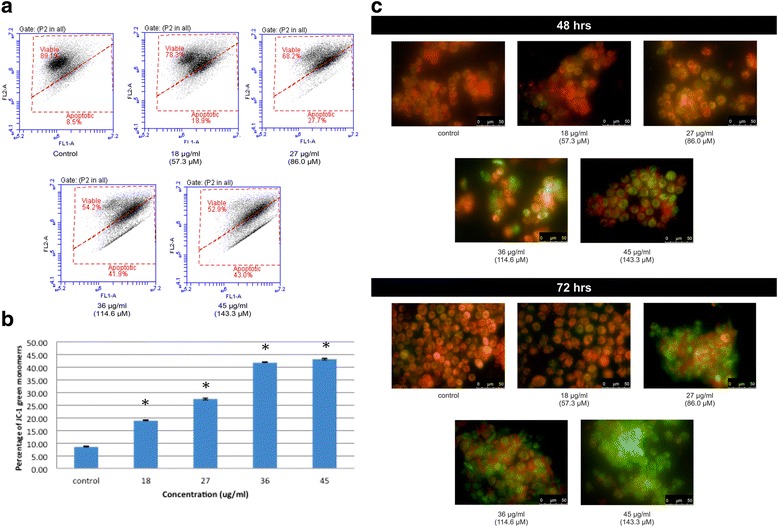
**a** Dot plots obtained from flow cytometer shows the attenuation of mitochondrial membrane potential (MMP) in A549 cells treated with chalepin at various concentrations and incubated at 48 h. **b** Bar chart represents the percentage of JC-1 that remained as green monomer which shows the apoptotic cells with compromised mitochondrial membrane potential upon treatment of chalepin in dose dependent manner. The data expressed as mean ± S.D from triplicates in experiment. Asterisks indicates significantly different value from control (*p < 0.05). **c** Effects of chalepin at different concentrations on mitochondrial membrane potential changes of A549 cell line at 48 h and 72 h treatment time. After the incubation period, the cells were stained with JC-1 dye and were examined with a fluorescent microscope under magnification of 630x to observe whether the JC-1 aggregates in mitochondria (healthy cells) or JC-1 remains in monomer form at cytoplasm (apoptotic cells)

Mitochondrial membrane potential (MMP) may be disrupted during early apoptosis. The loss of MMP in A549 cells which were treated with various concentrations of chalepin was observed using the JC-1 dye. During the early stage of apoptosis, mitochondrial depolarisation occurs and this enabled fluorescence of JC-1 to turn from red aggregates to green monomers. Figure 10a showed untreated control cell did not show any increase in green monomer formation. Upon treatment with chalepin at different concentrations, the percentage of green monomer increases in cytoplasm (Fig. 10b). This shows that, chalepin significantly induced reduction of mitochondrial membrane potential in A549 cells in a concentration dependant manner. Figure 10c shows that at 48 h incubation time, the untreated cells gives red fluorescence which indicates that there is high membrane potential in the mitochondrial membrane which enables the JC-1 dye to pass through the mitochondrial membrane and form aggregates which could give the red fluorescence. This shows that the cells are healthy. As the cells are treated with chalepin, there is increase in the presence of the green fluorescence as the concentration of chalepin treated is increased. When a cell undergoes apoptosis, the mitochondrial membrane potential would drop and this would inhibit the influx of JC-1 dye into mitochondria. It would remain in its initial monomeric form at cytoplasm which would give the green fluorescence that is observed in cells that were treated with high concentration of chalepin i.e., 36 μg/ml and 45 μg/ml. This observation is similar at 72 h incubation time with a difference of more fluorescence intensity at higher treatment time. We observed that the cells at 45 μg/ml treatment dosage at 72 h, almost all gives a green fluorescence. This indicates that almost all the cells that were observed is undergoing apoptosis.

**Fig. 11:**
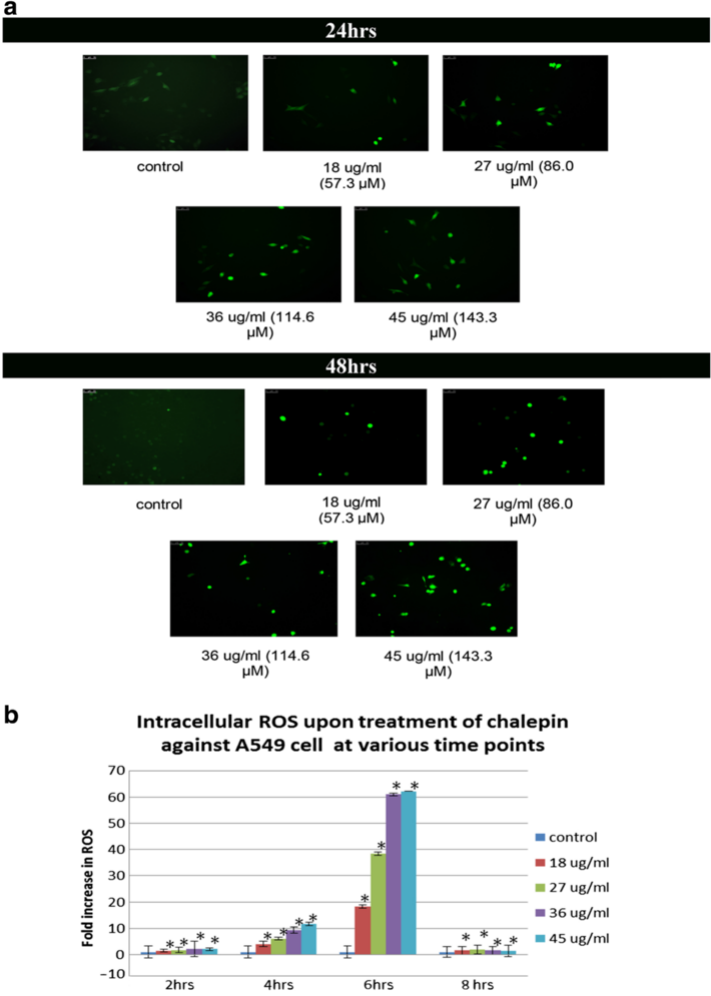
Elevation of the intracellular reactive oxygen species (ROS) level was observed qualitatively through observation of the cells after incubating with DCFH-DA dye and examining the changes using a fluorescent microscope and quantified using a fluorescence plate reader. **a** Induction of ROS in A549 cells treated with chalepin at various concentrations and incubated for 24 h and 48 h **b** Bar chart represents the fold increase of intracellular ROS level in A549 cells treated with different concentrations of chalepin as compared to untreated negative control cells. The data expressed as mean ± S.D. from three replicates. Asterisks indicates significantly different value from control (*p < 0.05)

The effect of chalepin in inititating the generation of intracellular ROS in A549 cells was detected using the DCFH-DA dye. Observation through fluorescent microscope was done on lung carcinoma cells after treatment of chalepin for 24 and 48 h showed an increase in intensity of green fluorescence of DCF in a dose and time dependant manner in (Fig. 11a). It was observed that, as the concentration of chalepin that was treated to the cells was increased, there was an increase in the intensity of green fluorescence by the DCFH-DA dye. This trend was also true for an increase in the incubation time. The DCFH-DA is a non-polar dye which would be converted into DCFH by cellular esterases. DCFH is non-fluorescent but is switched to fluorescent DCF when it is oxidized by ROS in the cell. Hence, the qualitative imagining showed that the increase in the intracellular ROS content is dependant upon the concentration of chalepin and also the incubation time of the treatment. Besides the qualitative imaging, the quantification of the intracellular content of ROS by measurement of the fluorescence of the DCF was also conducted. The fluorescence were then calculated in the ratio of fold increase of the fluorescence intensity in the treated cells as compared to the untreated negative control cells. The fold increase of intracellular ROS was calculated based on fluorescence value obtained from microplate reader. It was observed that in comparison to the control, the treated cells showed a gradual increase in the fold increase of intracellular ROS content starting at 2 h up till 6 h. This increase was time and dose dependant. However at 8 h, there was a drop in the fluorescent measurement of the treated cells (Fig. 11b). This observation could be due to the reason where cell death has commenced and thus there is a drop.

**Fig. 12:**
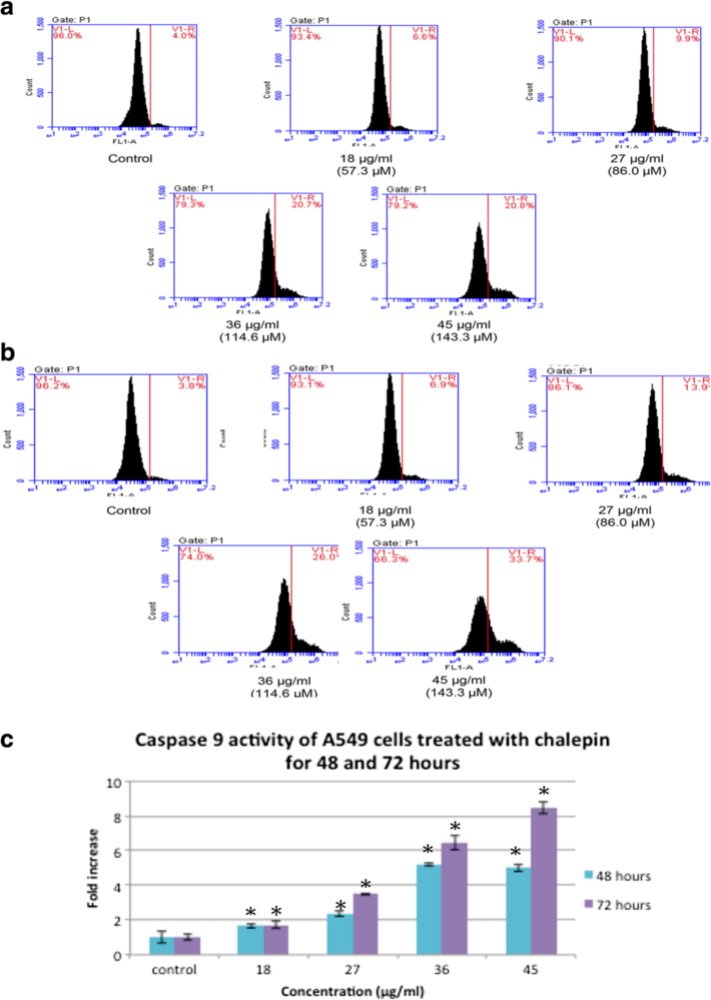
Caspase 9 activity of A549 cells treated with various concentrations of chalepin and incubated for 48 h and 72 h incubation. **a** Flow cytometric analysis of apoptotic (V1-R) and non-apoptotic populations (V1-L) for active caspase 9 activity for untreated A549 cells and A549 cells treated with chalepin at various concentrations for 48 h. **b** Flow cytometric analysis of apoptotic (V1-R) and non-apoptotic populations (V1-L) for active caspase 9 activity for untreated A549 cells and A549 cells treated with chalepin at various concentrations for 72 h. **c** Bar chart representing a comparison of fold increase between chalepin treated A549 cells at different incubation times (48 & 72 h). The data expressed as mean ± S.D. of three replicates. Asterisks indicates significantly different value from control (*p < 0.05)

Caspase 9 is associated with the activation of the intrinsic mitochondrial mediated pathway. CaspILLUME fluorescein active caspase-9 staining kit was used to determine the caspase 9 activity in regards to the treatment of chalepin in A549 cells. Cells with activated caspase 9 would move to the right side (V1-R) of the density plot. The results shows that there is a remarkable increase in the caspase 9 activity of cells treated with chalepin as compared to the control (Fig. 12). It was observed that after 48 h of incubation period there is a five-fold increase in activation of caspase 9 from 4.5 ± 0.35 % in untreated control to 22.57 ± 0.21 % in cells treated with 45 μg/ml of chalepin (Fig. 12a). As for the 72 h incubation period, there was an increase from 3.97 ± 0.15 % for untreated control cells to 33.5 ± 0.35 % for treated cells with 45 μg/ml of chalepin and this is a 8.44 fold increase (Fig. 12b). When cells receive apoptotic stimuli, the mitochondria releases cytochrome c which then binds to Apaf-1, together with dATP. The resultant complex recruits caspase-9 leading to its activation. Activated caspase-9 cleaves downstream caspases such as caspase-3, -6 and -7 initiating the caspase cascade [13]. This results shows that chalepin treated cells undergo apoptosis through the mitochondrial pathway.

**Fig. 13:**
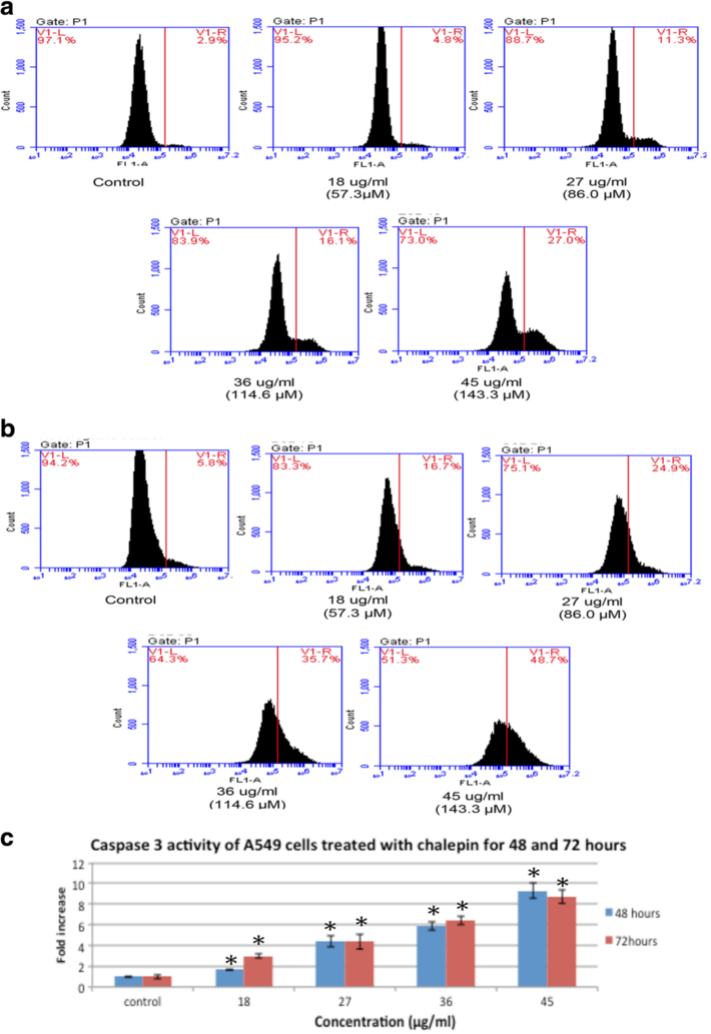
Caspase 3 activity of A549 cells treated with various concentrations of chalepin and incubated for 48 h and 72 h. **a** Flow cytometric analysis of apoptotic (V1-R) and non-apoptotic populations (V1-L) for active caspase 3 activity for untreated A549 cells and A549 cells treated with chalepin at various concentrations for 48 h. **b** Flow cytometric analysis of apoptotic (V1-R) and non-apoptotic populations (V1-L) for active caspase 3 activity for untreated A549 cells and A549 cells treated with chalepin at various concentrations for 72 h. **c** Bar chart representing the fold increase of caspase 3 activity in A549 cells treated with chalepin at various concentrations as compared to untreated control cells. **d** Bar chart representing a comparison of fold increase between chalepin treated A549 cells at different incubation times (48 & 72 h). The data expressed as mean ± S.D. of three replicates. Asterisks indicates significantly different value from control (*p < 0.05)

Caspase 3 is a downstream caspase signal also known as the effector caspase. Upon activation of caspase 9, a signal cascade results in an activation of caspase 3 which in turn results in the hydrolysis of more than 100 target protein that subsequently results in apoptosis. Defect in caspase 3 activity results in cancer. In this study, it was observed that A549 cells treated with chalepin induces caspase 3 activation. CaspILLUME fluorescein active caspase-3 staining kit was used and the cell population was analysed using a flow cytometer. In the event of caspase 3 activation, the cell population would move to the right hand side (V1-R) of the density plot (Fig 13). It is observed that after 48 h of incubation period there is an increase of 2.83 ± 0.06 % for untreated control to 26.40 ± 0.79 % for cells treated with 45 μg/ml of chalepin and this is a 9.32 fold increase (Fig. 13a). As for the 72 h incubation period, there was an increase from 5.63 ± 0.21 % for untreated control cells to 49.07 ± 0.63 % for treated cells with 45 μg/ml of chalepin and this is a 8.71 fold increase (Fig. 13b). Caspase-3 is required for some typical characteristics of apoptosis, and is crucial for apoptotic chromatin condensation and DNA fragmentation in all cell types. Caspase-3 is essential for certain processes associated with the dismantling of the cell and the formation of apoptotic bodies, but it may also function before or at the stage when commitment to loss of cell viability is made [14]. Activation of caspase 3 in chalepin treated A549 cells therefore is an evidence that apoptosis has commenced.

### Western blot analysis

**Fig. 14:**
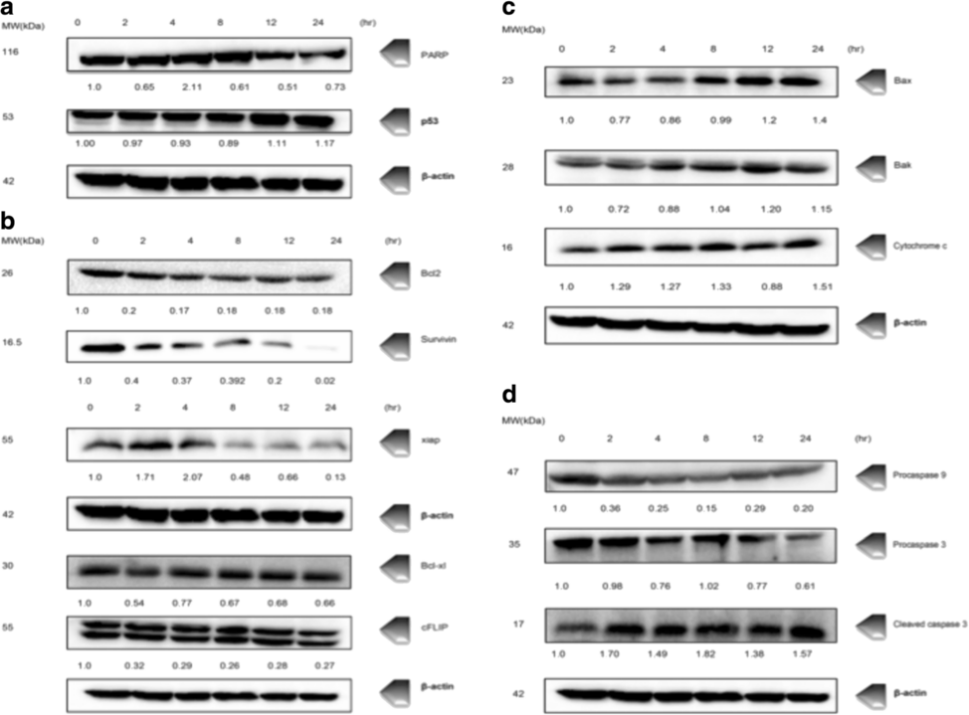
Western blot analysis of whole cell protein lysate of A549 cells treated with chalepin at concentration of (36 μg/ml) at 2,4,8,12, and 24 h. **a** Cleavage of PARP and upregulation of p53 tumor supressor factor, induced by chalepin.**b** Chalepin (36 μg/ml) inhibits anti-apoptotic gene products. **c** Chalepin induces Bax and bak expression and cytochrome c release. **d** Chalepin activates cleavage of procaspase 9 and procaspase 3

Changes in the expression of proteins involved in regulation of survival and cell death following chalepin treatment were examined by Western Blot analysis. Activation of caspase cascade would trigger proteolytic degradation of PARP and DNA fragmentation by endonucleases, leading to apoptosis. The expression of PARP was studied in A549 cells treated with chalepin at different incubation time. It was observed that the quantity of PARP expressed decreased with time. This shows that the level of PARP decreased with time as shown by Fig. 14a.

The mitochondrial mediated pathway is also known as BCL-2 regulated pathway. The balance of function between proapoptotic and antiapoptotic BCL-2 protein family determines the fate of cells towards apoptosis [15]. Studies have shown Bax, Bad, Bak promote the release of cytochrome c, while bcl-2 and Bcl-X_L_ delays this response and promote cell survival. Bcl-2 family members regulate the apoptotic response by controlling the mitochondrial membrane permeabilization (MMP). Depolarisation of MMP and the formation of MPT (mitochondrial permeability transition) pore is a result of the tranlocation of Bax from mitochondria to cytosol [16].

Chalepin was found to downregulate anti-apoptotic gene products such as Bcl-2, survivin, XIAP, Bcl-xl and cFLIP as shown by Fig. 14b This clearly shows that it downregulates proteins that play a vital role in the cell survival and thus, induces apoptosis. An upregulation in the expression of Bax was observed (Fig. 14c). There was an upregulation in the expression of cytochrome c, Fig. 14c, which shows that the cytochrome c moved out of mitochondria to cytosol leading to an upregulation of cytochrome c. All these factors promotes cell cytolysis or cytostasis.

The cytochrome c release and binding with dATP and Apaf-1 results in formation of apoptosome which recruits and activates caspase-9. Activated caspase 9, results in activation of effector caspases such as caspase-3/7. These effector caspases then initiates apoptosis events. Results indicated that chalepin induces cleavage of procaspases 9 and 3 to the active cleaved form. Procaspase 9 showed a downregulation which indicated cleavage of the protein to an active form. Procaspase 3, also showed downregulation which indicated an activation. This result is further stregthened by detection of the cleaved caspase 3 which showed an upregulation. All these results are illustrated in Fig. 14d. This clearly indicates that the intrinsic pathway which is mediated by the mitochondria is activated. These results are consistent with earlier caspase 3 and caspase 9 activity determination using flow cytometer.

## Discussions

This is the first report on the cytotoxic potential of the methanol and chloroform extracts on the human lung carcinoma (A549), human colon carcinoma (HCT-116) and human cervical carcinoma (CaSki) cells and also the human normal lung fibroblast (MRC5). A crude extract is considered to possess in vitro cytotoxicity if the IC_50_ value, at incubation time between 48 and 72 h, is less than 20 μg/ml [17]. Among all the extracts, the chloroform extract (without chlorophyll) exhibited the highest cytotoxic activity (IC_50_ value of 8.8 ± 0.32 μg/ml) and was thus selected for further investigation. The chloroform extract (without chlorophyll) exhibited better cytotoxicity than the total chloroform extract which could possibly be due to the removal of some inactive components by charcoal. Activated carbon was used as the absorbing material to remove the chlorophyll present in the extract, inevitably charcoal can also absorb other components besides chlorophyll.

The chloroform extract (without chlorophyll) was subjected to chemical isolation using the HPLC. Twelve (12) components were successfully isolated and were sufficient in quantity for further cytotoxicity screening. Among these compounds were graveoline, kokusaginine, bergapten, moskachan B, moskachan D, chalepensin, rutamarin, arborinine, chalepin and neophytadiene. Most of the compounds fall into the class of alkaloids, furanocoumarin and also dihydrofuranocoumarins. Arborinine, graveoline, moskachan B and moskachan D are alkaloids. Besides chalepin, moskachan D and arborinine showed good cytotoxic effect against A549 cell line at 72 h incubation with IC_50_ values of 18.5 ± 0.65 μg/ml and 13.1 ± 0.06 μg/ml respectively. A previous report showed that arborinine isolated from *Ruta graveolens* showed IC_50_ values of 1.84 μg/ml, 11.74 μg/ml and 12.95 μg/ml on cervical carcinoma (HeLa), breast adenocarcinoma (MCF-7) and skin epidermoid carcinoma (A431) respectively [18]. Several alkaloids isolated from natural herbs exhibit antiproliferative and antimetastasis effects on various types of cancers both in vitro and in vivo. Alkaloids, such as camptothecin and vinblastine, have already been successfully developed into anticancer drugs [19]. Rutamarin and chalepin are furanocoumarins and it was reported that coumarins are the most abundant in Rutaceae and Umbelliferae family [20]. Bergapten, a furanocoumarin, exhibited mild cytotoxicity with an IC_50_ value of 43.53 ± 1.81 μg/ml against A549, human lung carcinoma cell line. Chalepin exhibited excellent cytotoxocity activity (IC_50_ value of 8.69 ± 2.43 μg/ml) whereas rutamarin exhibited mild cytotoxicity (IC_50_ value of 68.5 ± 3.06 μg/ml). Rutamarin is the acetylated form of chalepin. A previous report [21] showed that rutamarin had an IC_50_ value of 1.318 μg/ml against A549 cell line. The reason for the lower activity of rutamarin in our hands, may be due to crystallisation in the media at higher concentration of rutamarin. Based on all the collective results, chalepin was selected for further investigation.

Cells undergoing apoptosis show typical, well-defined morphological changes which includes plasma membrane blebbing, chromatin condensation with margination of chromatin to the nuclear membrane, karyorhexis (nuclear fragmentation), and formation of apoptotic bodies [22]. The morphological changes of A549 cells treated with various concentrations of chalepin were observed using a phase contrast microscope (Fig. 6). Observation showed decrease in number of cells as the treatment concentration was increased at a magnification of 100×. Formation of apoptotic bodies, vacuolisation, nuclear condensation, echinoid spikes were visible at higher magnification (400×). Longer incubation period shows bigger change in the morphology of the cells. In contrast, the untreated control cells maintained a healthy structure and exponentially increased with time.

Early, late apoptosis or secondary necrosis, and necrosis were visualised using Hoechst 33342 and propidium iodide double staining assay. Hoechst 33342 dye can cross the nuclear membrane to tag the nucleus. Propidium iodide does not possess this capacity and could only cross compromised nuclear membrane to give a red staining. Nuclear membrane compromisation is a characteristic of late apoptosis or necrosis and this property could distinguish the cells undergoing early apoptosis or late apoptosis or necrosis. In primary necrosis, cytoplasmic swelling occurs and plasma membrane ruptures together with organelle breakdown but notably absence of nuclear fragmentation [23]. The low blue fluorescence represents viable cells, bright blue fluorescence represents cells undergoing early apoptosis, pink or red fluorescence with fragmented nuclear DNA represents cells at late apoptosis or secondary necrosis and red fluorescence with intact nucleus represents cells undergoing primary necrosis. The A549 cells treated with chalepin primarily showed bright blue fluorescence and the intensity of the fluorescence increased as treatment concentration of chalepin is increased. At higher concentraion and incubation time, pink or red fluorescence with fragmented DNA were observed (Fig. 7). An apoptotic event in vivo, ends with phagocytosis by phagocytes to remove the apoptotic bodies. However, in in vitro conditions where the phagocytes are absent, the cells that initially were in early apoptotic phase would transform to late apoptotic or secondary necrotic phase which is visualized by pink or red fluorescence with fragmented nuclei and this was observed at chalepin treatment of 45 μg/ml at both 48 and 72 h incubation. This could be an indication that at higher concentration of chalepin, the apoptosis cell death progresses faster. The primary necrotic cells are usually stained red but with uncompromised nucleus. The morphological studies gives a preliminary insight on the morphological changes and the type of death the cell undergoes upon treament with chalepin.

Several biochemical criteria characterize apoptosis. These include phophatidylserine (PS) exposure on the outer leaflet of plasma membrane, changes in mitochondrial membrane permeability, release of intermembrane space mitochondrial proteins and caspase-dependent activation and nuclear translocation of a caspase activated DNAse resulting in DNA cleavage and fragmentation [22]. Expression of cell surface markers results in the early phagocytic recognition of apoptotic cells by adjacent cells, thus permitting quick phagocytosis with minimal compromise to the surrounding tissue. Flow cytometric Annexin V-FITC/PI double staining analysis can be used to determine early apoptosis and to distinguish between apoptotic and necrotic cells. In this study, we observed that the Annexin V positive cells which is the collective cells at the phase of early and late apoptosis increased with the increase of treatment concentration of chalepin and the treatment time. This clearly shows that chalepin has apoptosis inducing property. DNA fragmentation was observed in the TUNEL assay. Chalepin treated A549 cells also exhibited DNA fragmentation in a concentration and time dependent manner as shown in Fig. 9. DNA fragmentation is a hallmark of apoptosis. The tightly controlled activation of the apoptosis-specific endonucleases provides an effective means to ensure the removal of unwanted DNA and the timely completion of apoptosis [23]. DNA which breaks exposes large amount of 3’-OH ends and this can serve as the starting point for terminal deoxynucleotidyl transferase (TdT) to add deoxyribonucleotides in a template independent manner. Addition of the deoxythymidine analog 5-bromo-2’-deoxyuridine 5’triphosphate (BrdUTP) to the TdT reaction acts to label the break sites. BrdU is then detected by an anti-BrdU antibody using immunohistochemical techniques.

Reactive oxygen species (ROS) are widely generated in biological systems. Due to this, humans have evolved antioxidant defence systems that limit their production. Intracellular production of reactive oxygen species such as •OH, O_2_^-^ and H_2_O_2_ is associated with the arrest of cell proliferation. Similarly, generation of oxidative stress in response to various external stimuli has been implicated in the activation of transcription factors and to the triggering of apoptosis [24]. It was described that ROS and the mitochondria plays a major role in apoptosis induction under both physiologic and pathologic conditions [25]. Interestingly, mitochondria are both source and target of ROS. In our experiment, we have observed that there was an increase in the intracellular ROS level upon treatment with chalepin concentration and time dependently (Fig. 11). The increase was significantly higher as compared to control for up to 6 h of incubation with chalepin but a sharp drop was noticed after 8 h. This could be due to the fact that after 8 h the A549 cells started to die. Our experiment is designed such that ROS is only measured in viable cells as the dye used could only stain viable cells. A sharp decrease in the ROS content after 8 h shows that cell death commences after 6 h of incubation. The highest ROS fold increase was observed up to 62.24 fold at 45 μg/ml chalepin with 6 h incubation. ROS promotes outer membrane permeabilization and mitochondria-to-cytosol translocation of cytochrome c, AIF or Smac/Diablo and triggers the caspase cascade. Thus, ROS stimulates apoptotic pathway [26]. Irrespective of the morphological features of end-stage cell death (that may be apoptotic, necrotic, autophagic, or mitotic), mitochondrial membrane permeabilization (MMP) is frequently the decisive event that determines the survival and death of a cell [27]. The treatment of chalepin on A549 cells at 48 h incubation showed an increase in the reduction of mitochondrial membrane potential in a dose dependent manner (Fig. 10). This shows that chalepin induces disruption in the mitochondrial membrane potential. Depolarisation of the mitochondrial membrane often leads to the release of cytochrome c into the cytoplasm.

Caspases (cysteinyl aspartate-specific proteases) are synthesized as dormant proenzymes that, upon proteolytic activation, acquire the ability to cleave key intracellular substrates, resulting in the morphological and biochemical changes associated with apoptosis [28]. The caspase 9 activity was observed initially based on the analysis of cell population in a flow cytometer. The results showed that the caspase 9 activity was activated in A549 cells treated with chalepin in a concentration and time dependent manner. Caspase-9 is a member of the caspase family of cysteine proteases that have been implicated in apoptosis and cytokine processing. When cells receive apoptotic stimuli, the mitochondria releases cytochrome c which then binds to Apaf-1, together with dATP. The resultant complex recruits caspase-9 leading to its activation. Activated caspase-9 cleaves downstream caspases such as caspase-3, -6 and -7 initiating the caspase cascade [13]. Our results show that caspase 9 was activated which led to the downstream effector and the downstream effector caspase cascade. Caspase 3 or better known as the effector caspase is the caspase that executes the order received from caspase 9 to initiate apoptosis physically. In our preliminary analysis caspase 3 was activated in the A549 cells treated with chalepin in a concentration and also time dependent manner (Fig. 13). Caspase-3 is a frequently activated death protease, catalyzing the specific cleavage of many key cellular proteins [14] which eventually will result in DNA fragmentation as illustrated by the results from the TUNEL assay.

The mitochondria play an important role in coordinating caspase activation through the release of cytochrome c [28]. The mitochondrial-mediated pathway is also known as BCL-2 regulated pathway, intrinsic pathway and stress-induced pathway [14]. Western blot technique was employed to study the regulation expression of apoptotis related proteins. The Bcl-2 family members determine the liberation of mitochondrial protein [15]. Apoptotic threshold is set by interactions on the mitochondrial outer membrane between three functionally and structurally distinct subgroups of the BCL‐2 protein family: BH3 (the BCL‐2 homology 3) proteins (which convey signals to initiate apoptosis), the pro‐survival cell guardians such as BCL‐2 itself, and the pro‐apoptotic effector proteins BAX (BCL‐2‐associated X protein) and BAK (BCL‐2 antagonist/killer). Thus, this family can be regarded as a tripartite apoptotic switch. When enough BH3‐only proteins have been stimulated in response to various cytotoxic stresses to exceed the apoptotic threshold, BAX and/or BAK begin to oligomerize to a pore that permeabilize the mitochondrial outer membrane. This releases apoptogenic factors into the cytosol, particularly cytochrome *c*, which promotes the activation of procaspase 9 on APAF1; activated caspase 9 in turn processes and activates the effector caspases i.e., caspase 3 [15]. In the western blot analysis, it was observed that the Bcl-2 protein and Bcl-XL, pro-survival cell proteins which also inhibit apoptosis, was downregulated in a time dependent manner (0,2,4,8,12,24 h). Bax and Bak was upregulated time dependently and this shows that the promoter of apoptosis is upregulated.

p53, is a critical tumor suppressor. It functions to induce apoptosis as a result of DNA damage, hypoxia and oncogenic activation. Various biological functions such as cell cycle arrest, angiogenesis, senescence, metastasis, metabolism, and autophagy is associated to p53 [29]. In this study, upon treatment with chalepin, it was observed that there was an upregulation in the expression of p53 in Fig. 14(a). Bax is directly activated by the p53 tumor suppressor proteins following stress induction or indirectly through the p53-activation of the Bcl-2 pro-apoptotic member Noxa and PUMA, or throught the p53-independant mechanisms. The downregulation of Bcl-2 is necessary to prevent it blocking Bax oligomerization. Formation of the Bax pore, as well as the loss of mitochondrial potential causes cytochrome c to leak out of the mitochondria to cytosol [30]. We observed an increase in the level of cytochrome c in a time dependent manner. Cytochrome c would in turn activate the cleavage of procaspase 9 which is the initiator caspase, in which we observe in our study with a downregulation of the protein as time increases. Caspase 9 would inititate a signal cascade of caspases in which caspase 3 which is the effector caspase is activated. In this study it was observed that, procaspase 3 was downregulated which indicates the activation of procaspase 3 and an upregulation in the cleaved caspase. Inhibitor of apoptosis (IAP) proteins works in multiple ways in cell death regulation, ranging from inhibition of apoptosis and necrosis to the regulation of cell cycle and inflammation. Due to their ability to control cell death and elevated expression in a variety of cancer cell types, IAP proteins are attractive targets for the development of novel anti-cancer treatments [31]. Some of the inhibitor of apoptosis such as survivin, XIAP, cFLIP, Bcl-2, Bcl-xl were found to be downregulated which allows apoptosis to proceed.

## Conclusion

**Fig. 15:**
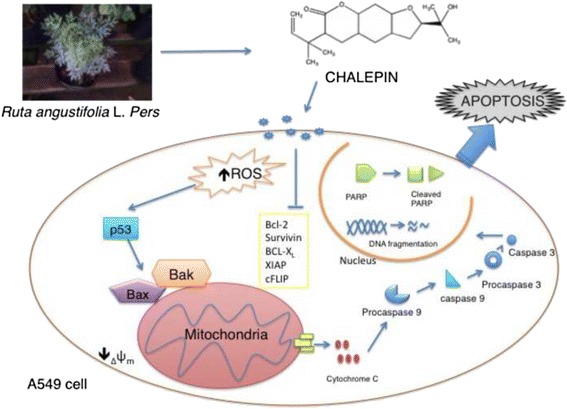
Graphical summarization of the action of chalepin in inducing mitochondrial mediated apoptosis in A549 cancer cell lines

In conclusion, this is the first report on the cytotoxic effect of chalepin isolated from *R.angustifolia* L.*Pers* against A549 cell line by induction of apoptosis. Apoptosis was found to be mediated by the mitochondria through Bax and Bak upregulation, The downregulation of inhibitor of apoptosis such as Bcl-2, survivin, Bcl-xl and cFLIP downregulation and release of cytochrome c and activation of caspases 9 and 3 to induce apoptosis. Chalepin was also found to exert DNA fragmentation and PS externalisation in A549 cells treated with it. This compound could be an excellent candidate to be considered as a therapeutic agent. This research is summarized graphically as in Fig. 15.

## Additional files

Additional file 1: Manuscript of Wu et al., 2003, i.e. reference [6], entitled "Cytotoxic and antiplatelet aggregation principles of Ruta graveolens." (PDF 1 mb)
Additional file 2: Manuscript of Del Castillo et al., 1986, i.e. reference [7], entitled "Four aromatic derivatives from Ruta angustifolia." (PDF 131 kb)
Additional file 3: Manuscript of Yang et al., 2013, i.e. reference [10], entitled "Bioactive coumarins from Boenninghausenia sessilicarpa." (PDF 192 kb)
Additional file 4: Manuscript of Orlita et al., 2008, i.e. reference [11], entitled "Identification of Ruta graveolens L. metabolites accumulated in the presence of elicitors." (PDF 115 kb)
Additional file 5: Manuscript of Orlita et al., 2008, i.e. reference [12], entitled "Application of chitin and chitosan as elicitors of coumarins and furoquinolone alkaloids in Ruta graveolens L. (common rue)." (PDF 80 kb)
Additional file 6: GCMS analysis of isolated compounds which includes the total ion chromatogram (TIC) and mass spectrum data. (PDF 12380 kb)

## Abbreviations

5-BrdUTP: 5-Bromo-2′-deoxyuridine 5′-triphosphate
ACN: Acetonitrile
AIF: Apoptosis-inducing factor
ALS: Automatic Liquid Sampler
ANOVA: Analysis of variance
ATCC: American type culture collection
Bak: BCL-2 homologous antagonist/killer
Bax: BCL2-associated X protein
BCL-2: B-cell CLL/lymphoma 2
Bcl-xL: B-cell lymphoma extra-large
BH3: Bcl-2 homology 3
BID: BH3-interacting domain death agonist
Caspases: Cysteinyl aspartate-specific proteases
cFLIP: Cellular FLICE-like inhibitory protein
CO_2_: Carbon dioxide
DAD: Diode array detector
dATP: Deoxyadenosine triphosphate
DCFH-DA: 2’7’-dichlorofluorescein diacetate
DMSO: Dimethyl sulfoxide
DNA: Deoxyribonucleic acid
ECL: Enhanced chemiluminescence
EDTA: Ethylenediaminetetraacetic acid
EIMS: Electron ionization mass spectrometry
ELISA: Enzyme-linked immunosorbent assay
EMEM: Eagle's minimal essential medium
EtOAc: Ethyl acetate
FBS: Feotal bovine serum
FC-AS: Fraction collector – Auto sampler
FITC: Fluorescein isothiocyanate
GCMS: Gas chromatography mass spectrometry
HPLC: High performance liquid chromatography
HRP: Horseradish peroxidase
IAPs: Inhibitor of apoptosis proteins
IC_50_: Inhibitory concentration at half maximal inhibitory
JC-1: 5,5’,6,6’-tetrachloro-1,1’,3,3’-tetraethylbehzimidazolyl carbocyanine iodide
LL: Lower left
LR: Lower right
m/z: mass-to-charge ratio
MeOH: Methanol
MMP: Mitochondrial membrane potential
MPT: Mitochondrial permeability transition
MS: Mass spectrometry
NaCl: Natrium chloride
NaVO_4_: Sodium orthovanadate
NIST: National Institute of Standards and Technology
NMR: Nuclear magnetic resonance
PARP: Poly ADP (Adenosine Diphosphate)-Ribose Polymerase
PBS: Phosphate buffered saline
PI: Propidium iodide
PMSF: Phenylmethyl-sulfonyl fluoride
PS: Phosphatidylserine
PUMA: p53 upregulated modulator of apoptosis
Rf: Retention factor
ROS: Reactive Oxygen Species
RPMI: Roswell Park Memorial Institute
SD: Standard deviation
SDS-PAGE: Sodium dodecyl sulfate polyacrylamide gel electrophoresis
Smac: Second mitochondria-derived activator of caspase
SPSS: Statistical package for the social sciences
SRB: Sulforhodamide
TCA: Trichloroacetic acid
TCC: Thermostatted column compartment
TdT: Terminal deoxynucleotidyl transferase
TLC: Thin layer chromatography
TUNEL: Terminal deoxynucleotidyl transferase dUTP nick end labeling
UL: Upper left
UR: Upper right
US: United States
USA: United States of America
UV: Ultraviolet
XIAP: X-linked inhibitor of apoptosis protein
