# Supplementary material for: Chalepin: isolated from Ruta angustifolia L. Pers induces mitochondrial mediated apoptosis in lung carcinoma cells
Source: BMC Complement Altern Med. 2016 Oct 12;16:389. doi: 10.1186/s12906-016-1368-6 (PMC5059921; doi:10.1186/s12906-016-1368-6)
Supplement: Additional file 1: — Manuscript of Wu et al., 2003, i.e. reference [6], entitled "Cytotoxic and antiplatelet aggregation principles of Ruta graveolens." (PDF 1 mb) [file 12906_2016_1368_MOESM1_ESM.pdf]

(a)

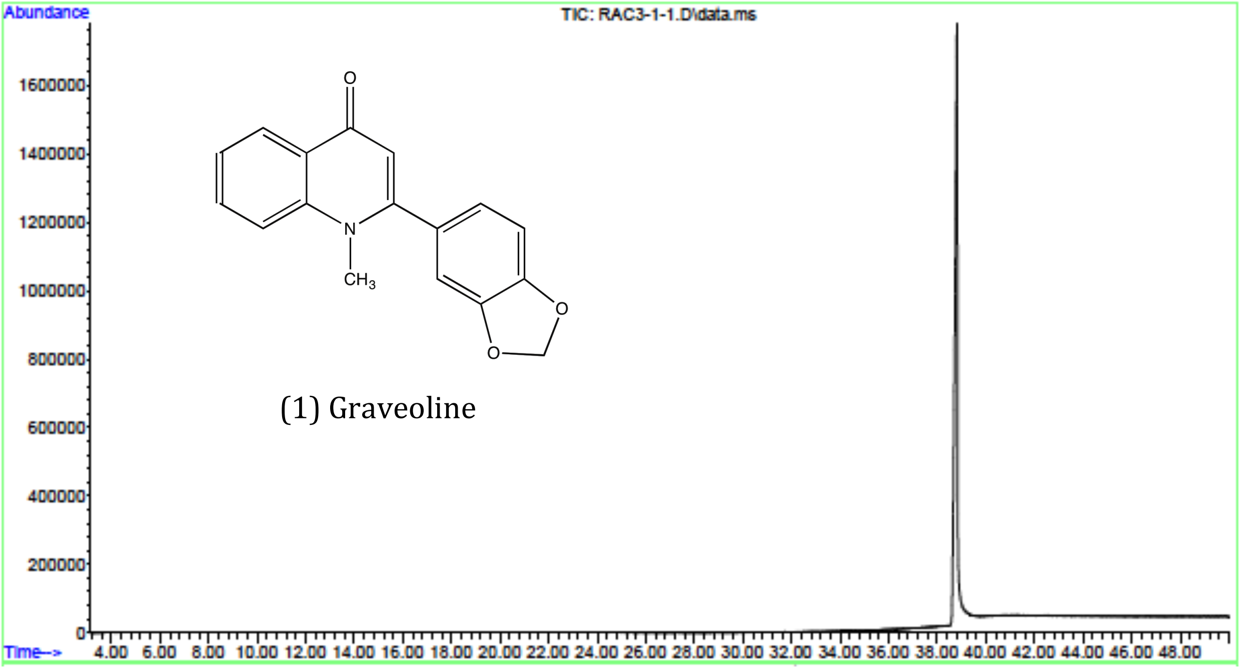

(b)

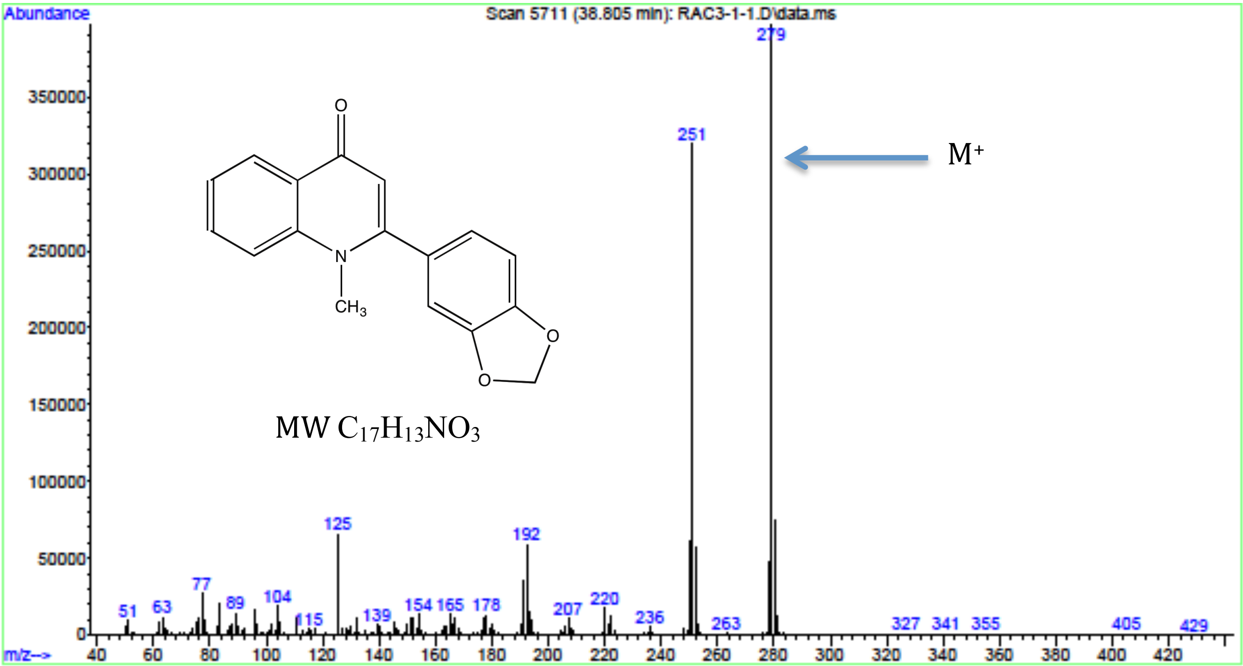

Figure : GCMS analysis of Graveoline (a) Total ion chromatogram (b) Mass spectrum

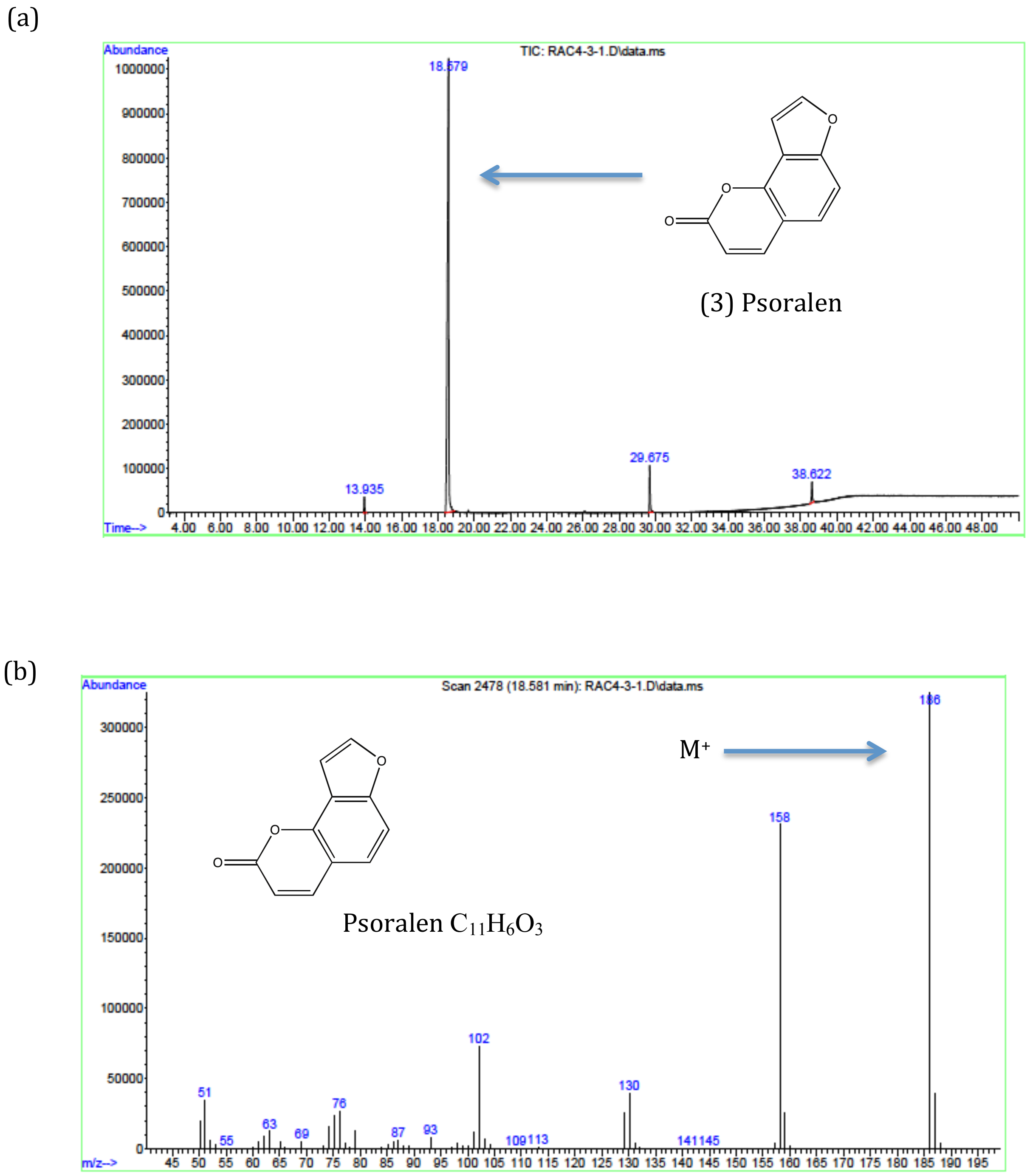

Figure : GCMS analysis of Psoralen (a) Total ion chromatogram (b) Mass spectrum

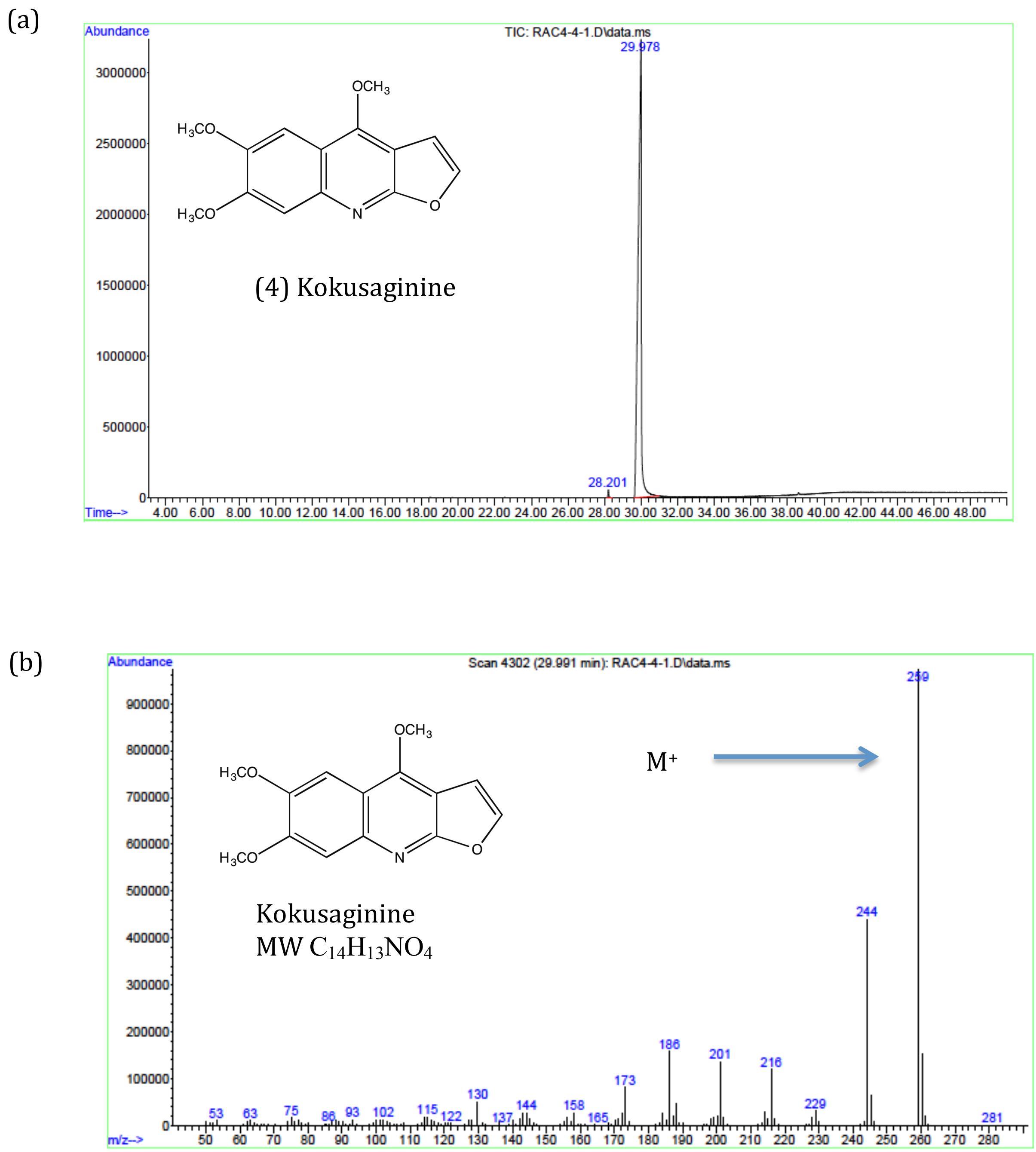

Figure : GCMS analysis of Kokusaginine (a) Total ion chromatogram (b) Mass spectrum

(a)

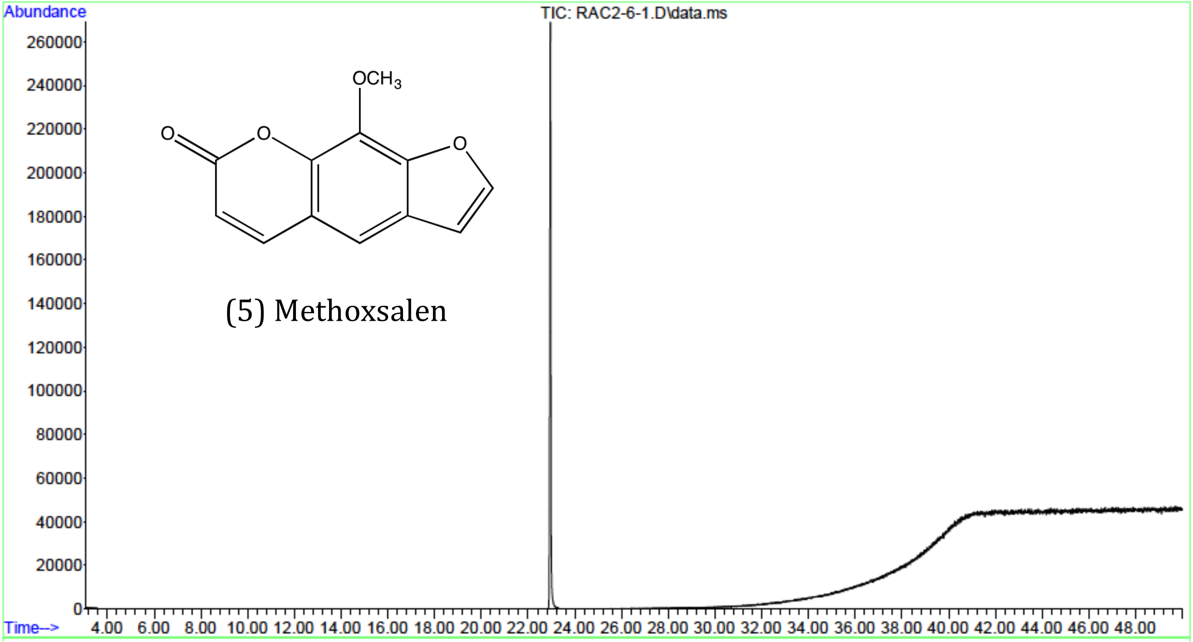

(b)

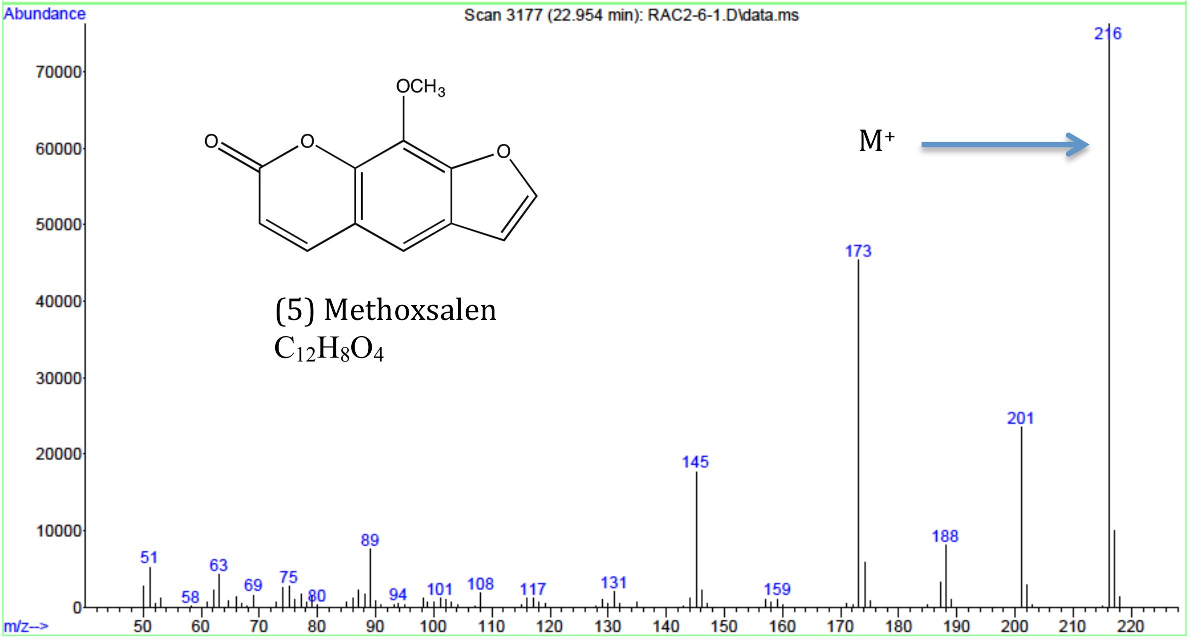

Figure : GCMS analysis of Methoxsalen (a) Total ion chromatogram (b) Mass spectrum

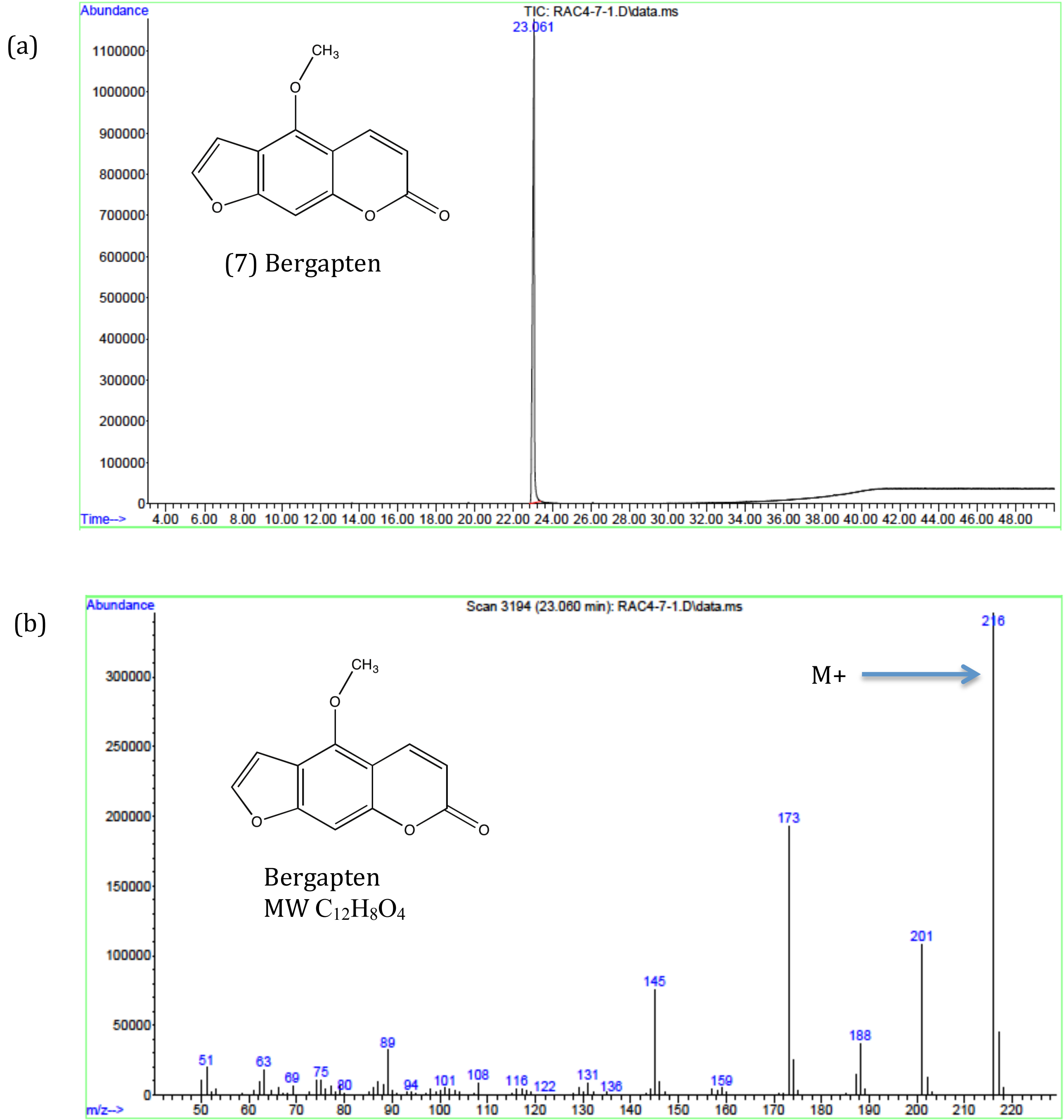

Figure : GCMS analysis of Bergapten (a) Total ion chromatogram (b) Mass spectrum

(a)

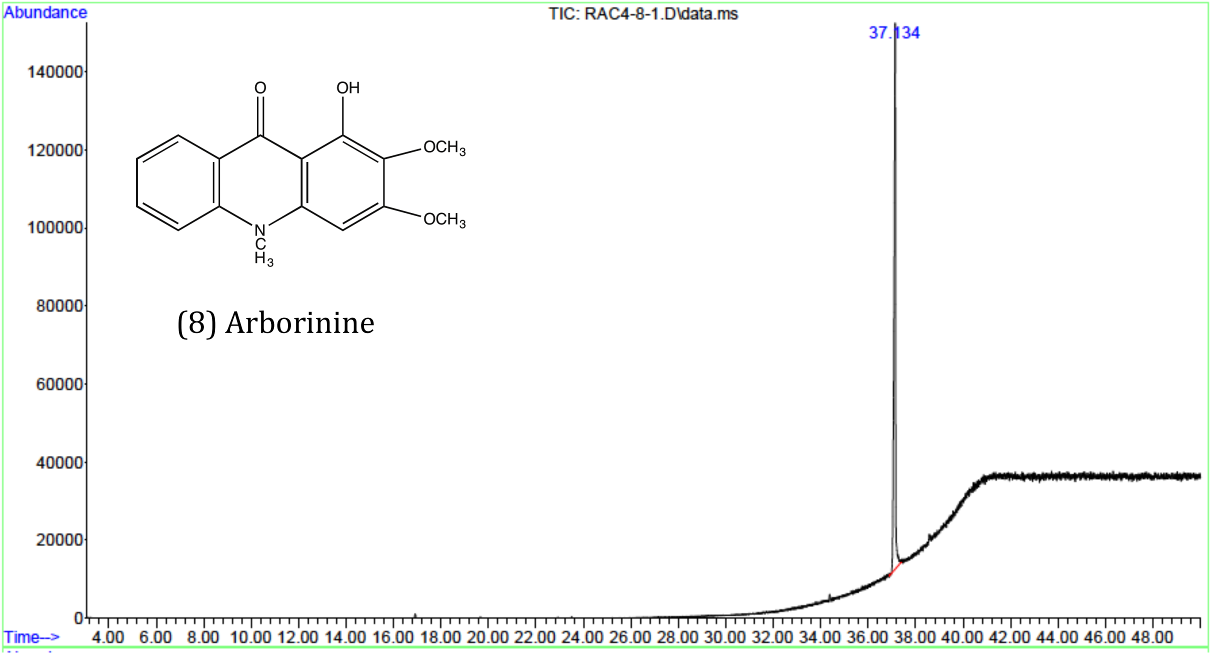

(b)

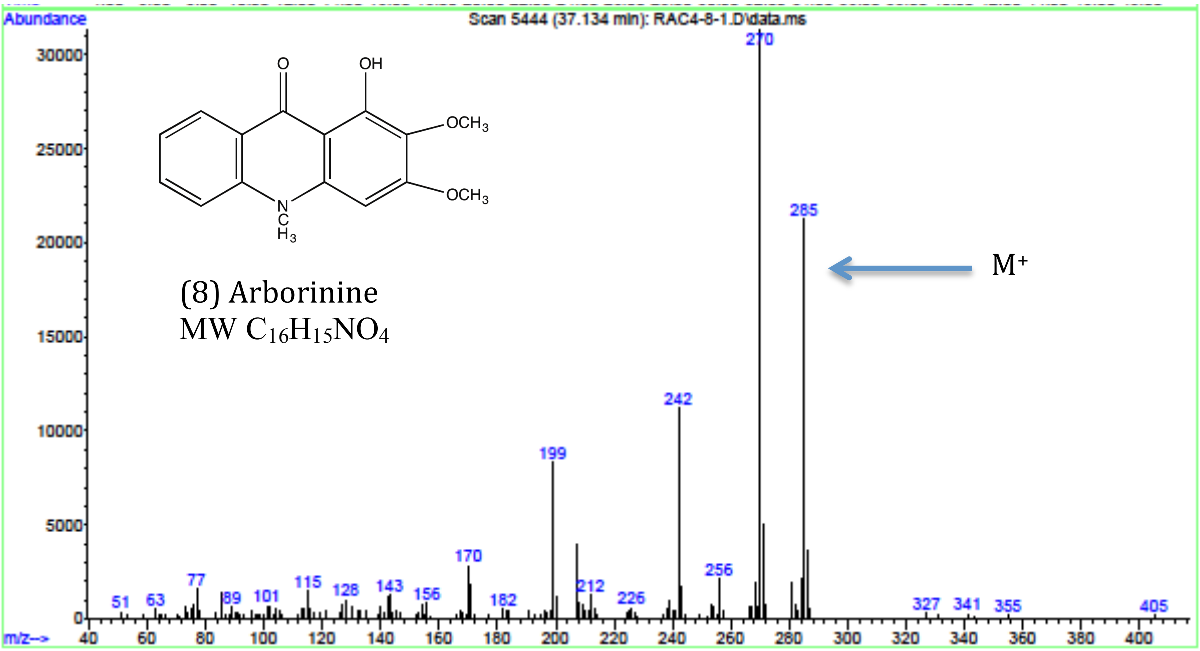

Figure : GCMS analysis of Arborinine (a) Total ion chromatogram (b) Mass spectrum

(a)

(b)

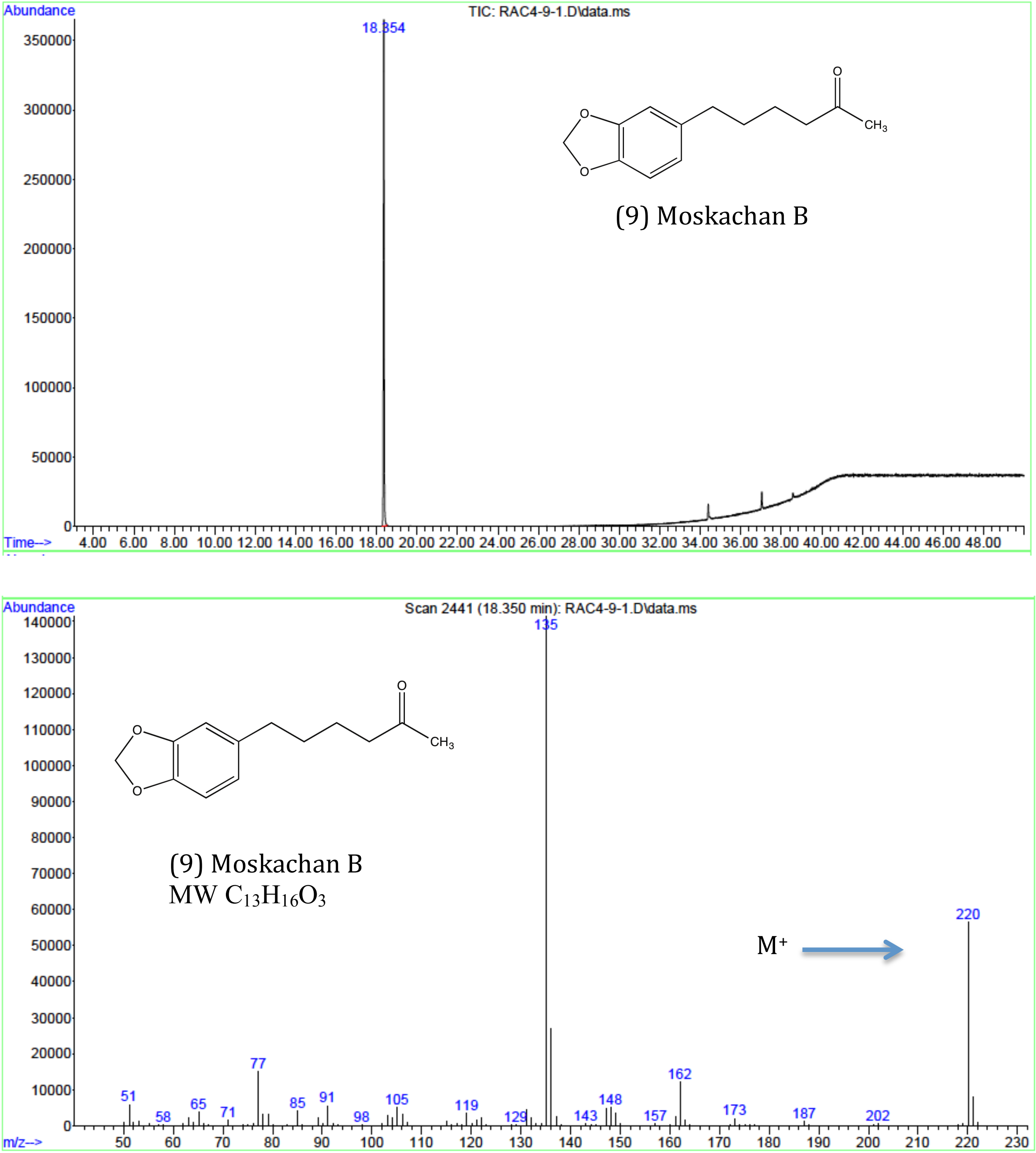

Figure : GCMS analysis of Moskachan B (a) Total ion chromatogram (b) Mass spectrum

(a)

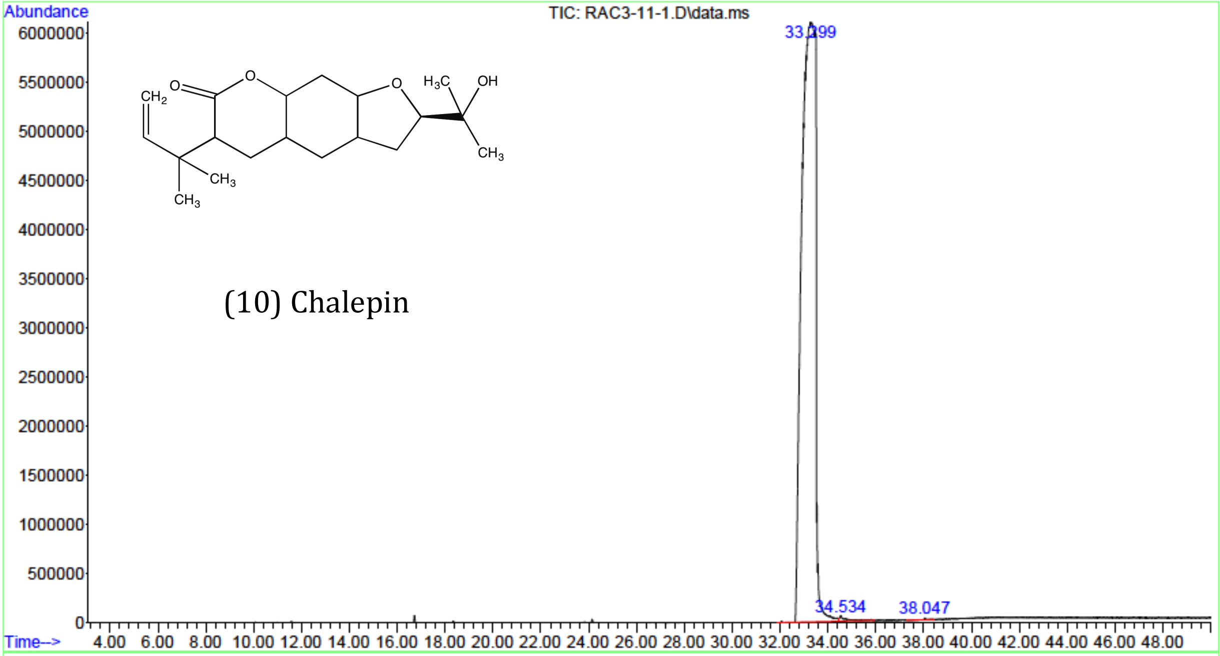

(b)

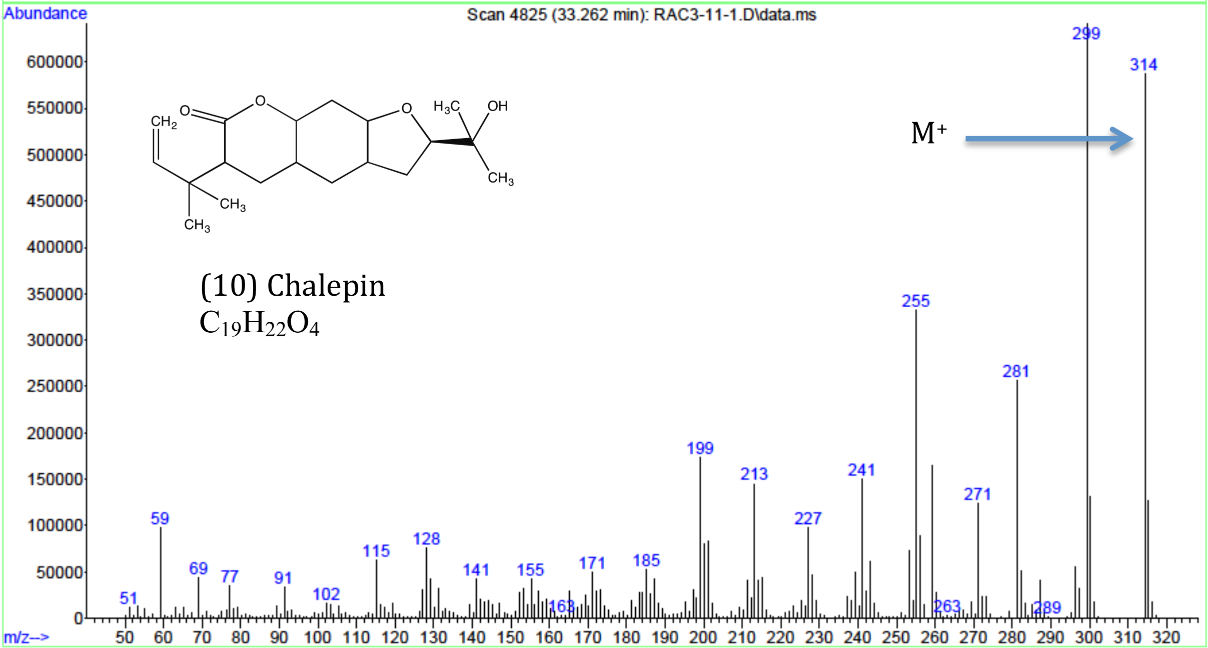

Figure : GCMS analysis of Chalepin (a) Total ion chromatogram (b) Mass spectrum

(a)

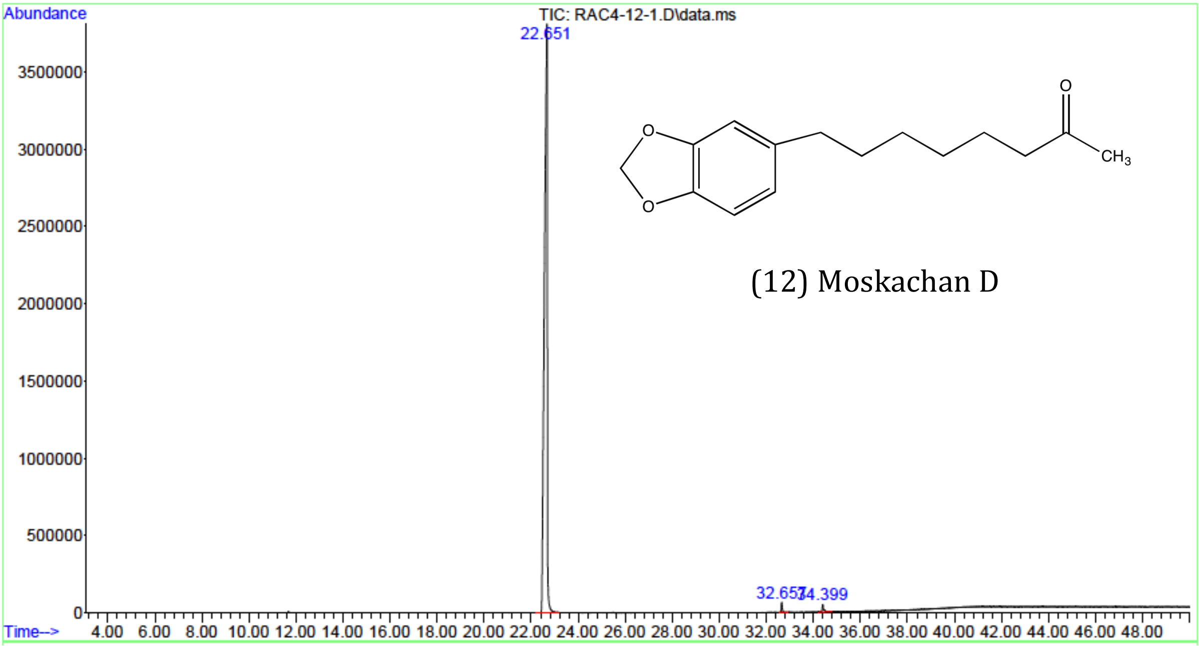

(b)

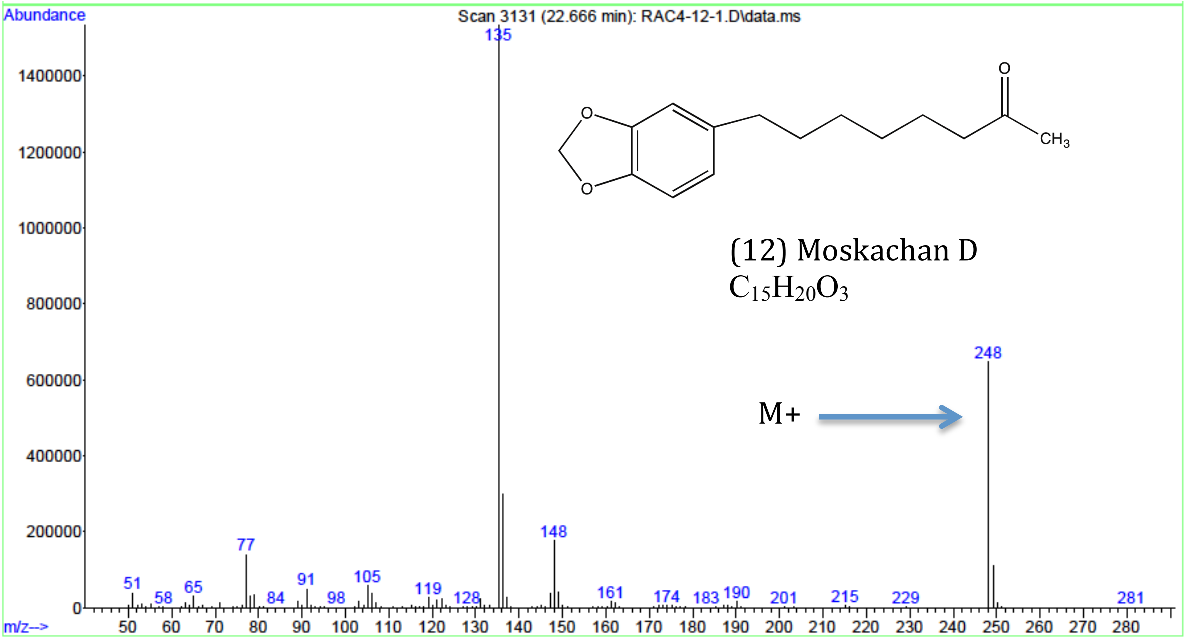

Figure : GCMS analysis of Moskachan D (a) Total ion chromatogram (b) Mass spectrum

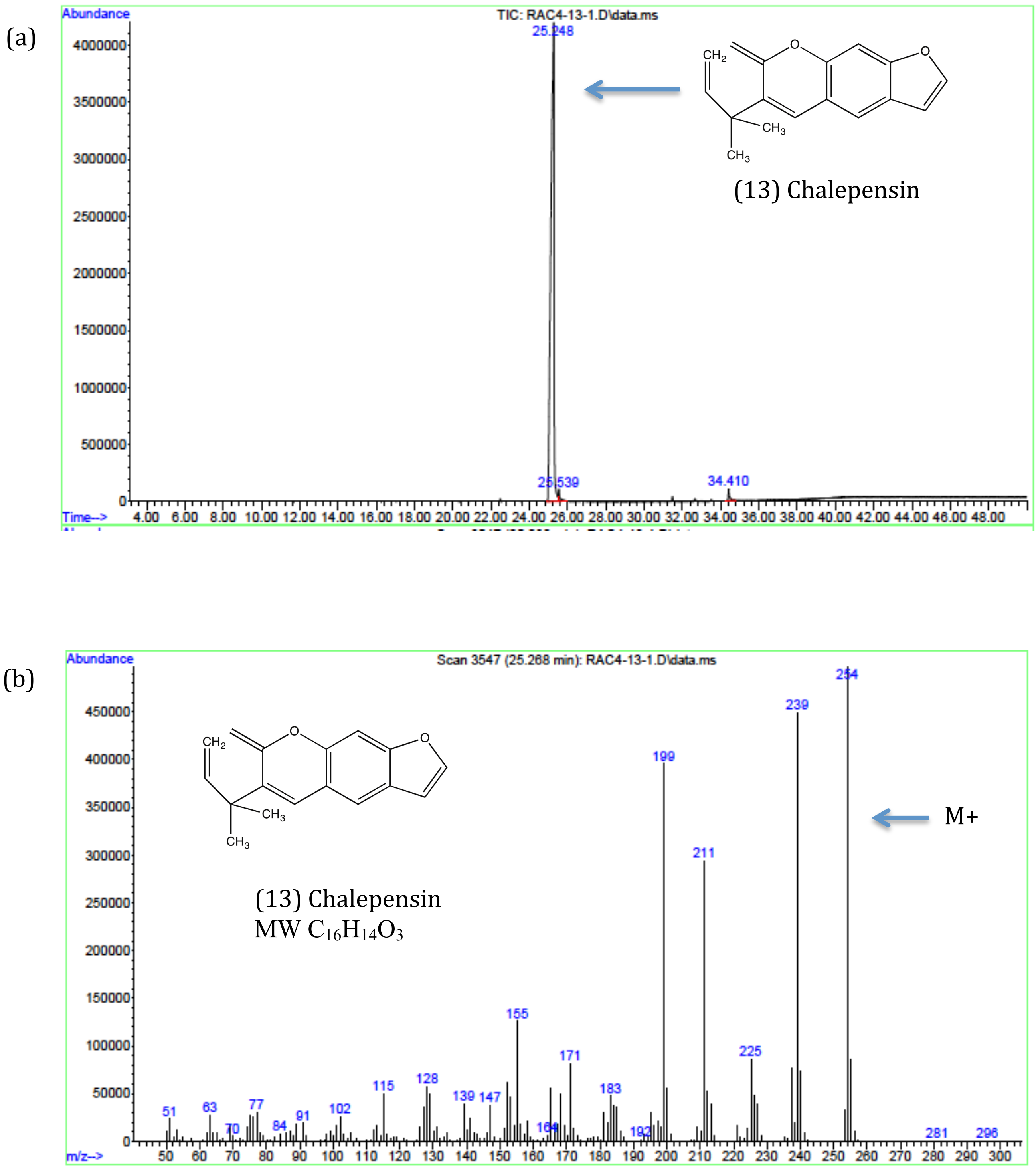

Figure : GCMS analysis of Chalepensin (a) Total ion chromatogram (b) Mass spectrum

(a)

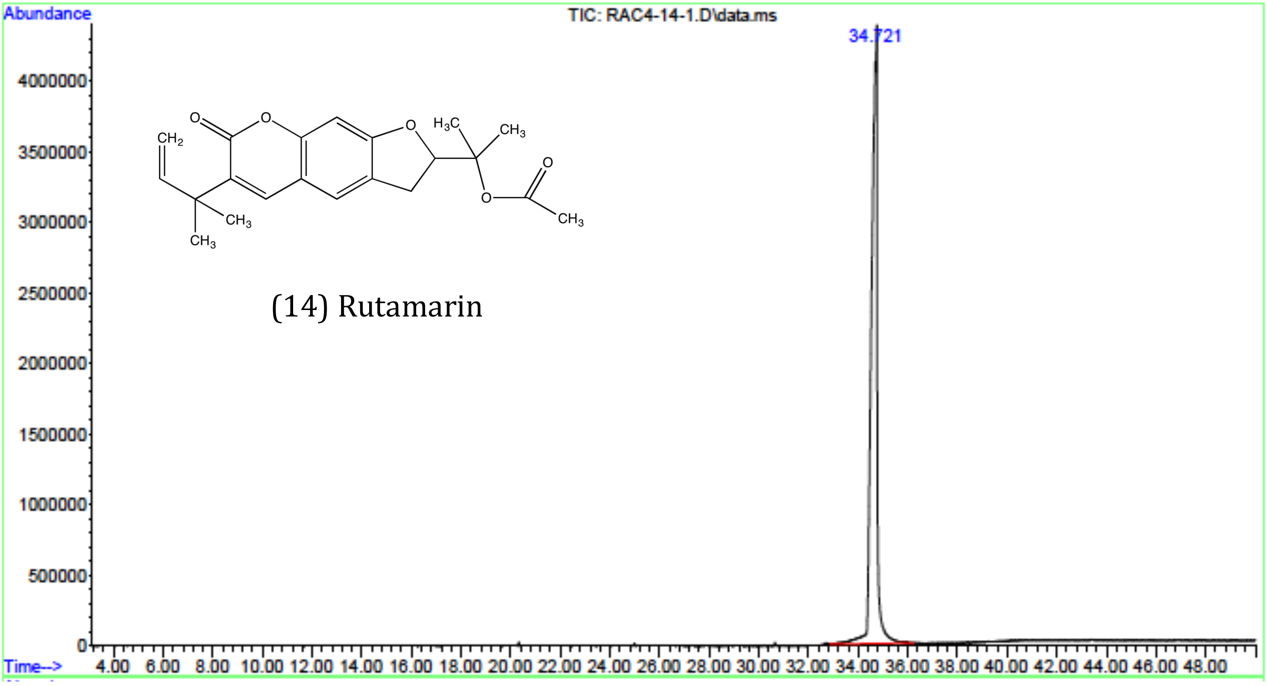

(b)

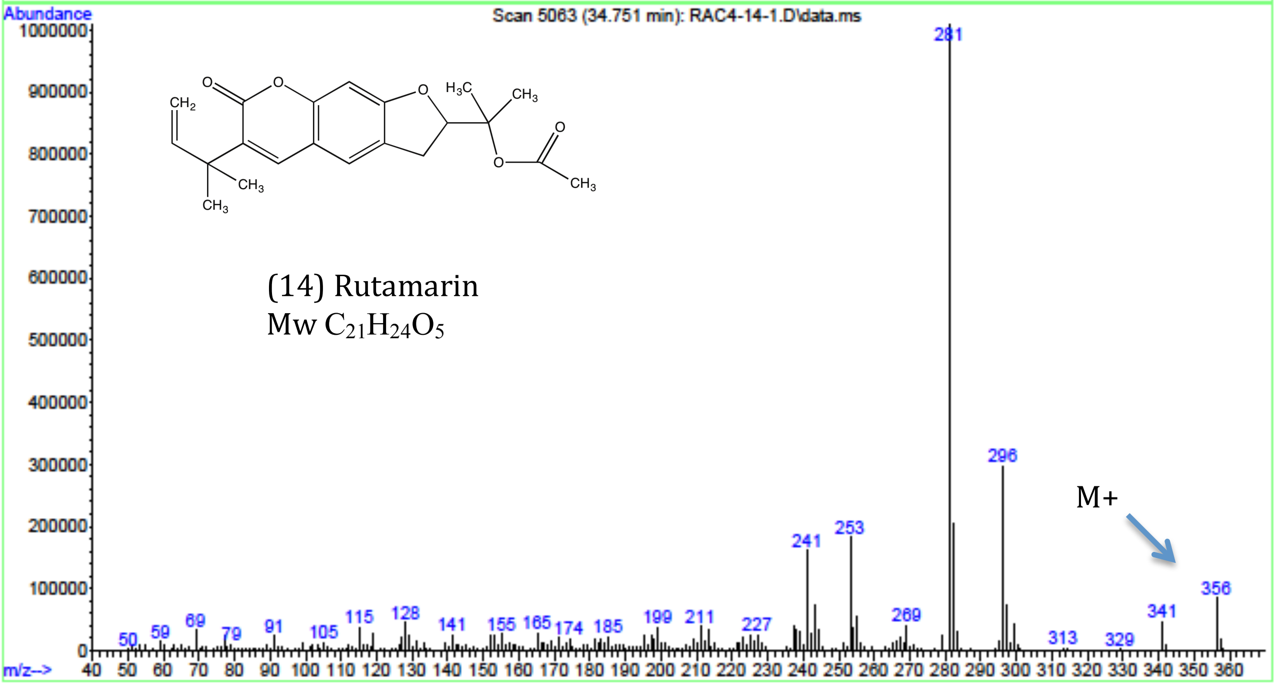

Figure : GCMS analysis of Rutamarin (a) Total ion chromatogram (b) Mass spectrum

(a)

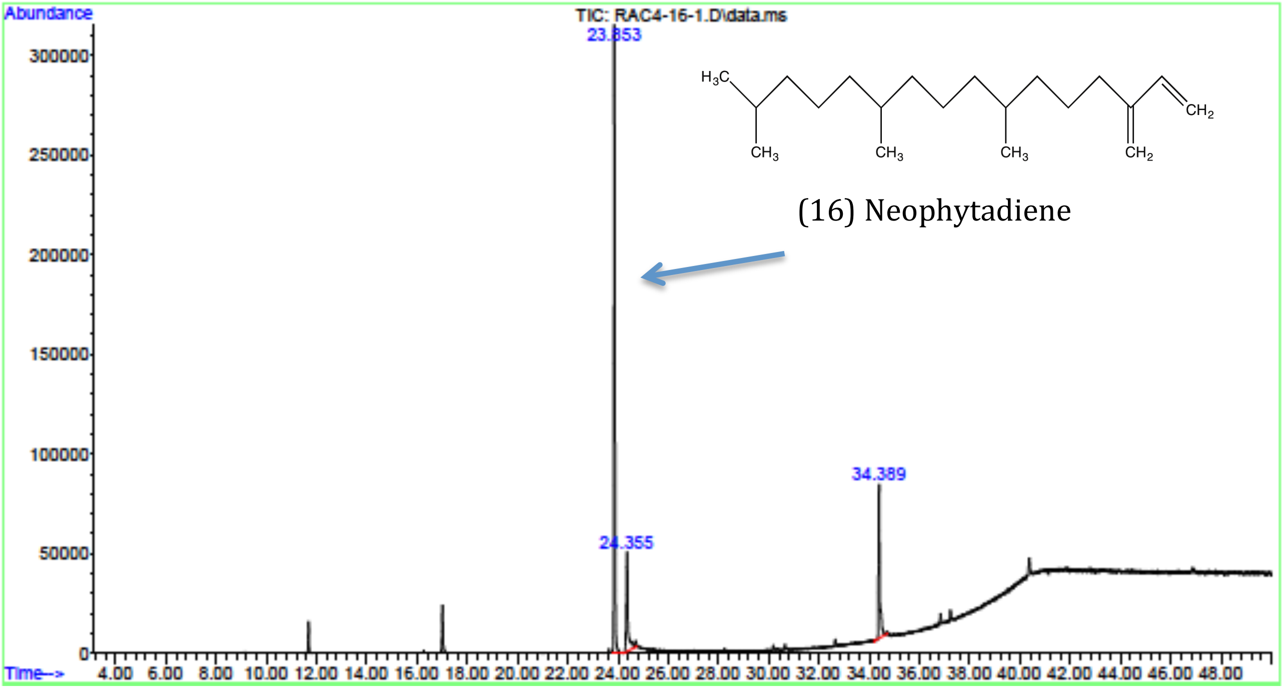

(b)

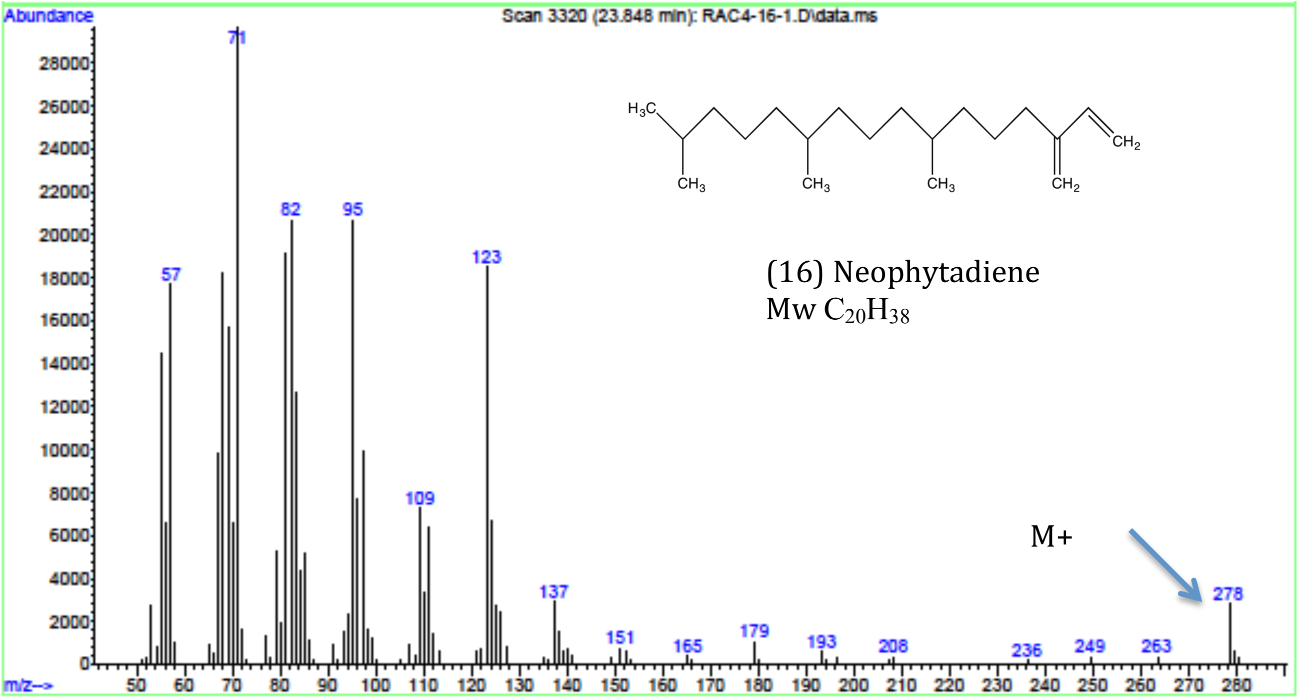

Figure : GCMS analysis of Neophytadiene (a) Total ion chromatogram (b) Mass spectrum
