# Supplementary material for: Chalepin: isolated from Ruta angustifolia L. Pers induces mitochondrial mediated apoptosis in lung carcinoma cells
Source: BMC Complement Altern Med. 2016 Oct 12;16:389. doi: 10.1186/s12906-016-1368-6 (PMC5059921; doi:10.1186/s12906-016-1368-6)
Supplement: Additional file 2: — Manuscript of Del Castillo et al., 1986, i.e. reference [7], entitled "Four aromatic derivatives from Ruta angustifolia." (PDF 131 kb) [file 12906_2016_1368_MOESM2_ESM.pdf]

# Cytotoxic and Antiplatelet Aggregation Principles of *Ruta graveolens*

Tian-Shung Wu[a]* ( 吳天賞 ), Li-Shian Shi[a] ( 石麗仙 ), Jhi-Joung Wang[b] ( 王志中 ),
Song-Chou Iou[c] ( 游松洲 ), Hsien-Chang Chang[c] ( 張憲昌 ), Yuh-Pan Chen[c] ( 陳玉盤 ),
Yao-Haur Kuo[d] ( 郭曜豪 ), Ya-Ling Chang[e] ( 張雅玲 ) and Che-Ming Teng[e] ( 鄧哲明 )
[a]*Department of Chemistry, National Cheng Kung University, Tainan, Taiwan 701, R.O.C.*
[b]*Department of Medical Research, Chi-Mei Foundation Hospital, Tainan, Taiwan, R.O.C.*
[c]*Brion Research Institute of Taiwan, Taipei, Taiwan, R.O.C.*
[d]*National Research Institute of Chinese Medicine, Taipei, Taiwan, R.O.C.*
[e]*Pharmacological Institute, College of Medicine, National Taiwan University, Taipei, Taiwan, R.O.C.*

Nineteen compounds have been isolated from the methanol extract of the root and aerial parts of *Ruta graveolens*. The structural elucidation of these isolated compounds were determined by the spectroscopic methods and/or comparison of the physical data with literature values. Their antiplatelet aggregation and cytotoxic activities were examined to find potent antiplatelet aggregation and cytotoxic compounds from natural resources. Among them, dictamine (**5**), skimmianine (**7**), psoralen (**8**), chalepensin (**12**), clausindin (**13**), and graveolinine (**16**) showed significant inhibition of platelet aggregation, induced by arachidonic acid and collagen. Arborinine (**2**), dictamine (**5**), isopimpinellin (**11**), clausindin (**13**), and graveoline (**17**) exhibited cytotoxic activity against KB, Hela, DLD, NCI and Hepa tumor cell lines.

**Keywords:** *Ruta graveolens*; Rutaceae; Structure elucidation; Antiplatelet aggregation activity; Cytotoxicity.

## INTRODUCTION

*Ruta graveolens*, a plant of Rutaceae, is native to Europe and cultivated as ornamental plant in southern China and Taiwan. It has been used in folk medicine as an antipyretic, diuretic and antitoxic, and to dispel the wind.[1] *R. graveolens* was also reported to have spasmolytic activity to the isolated rabbit ileum, ganglion-blocking and curare-like activity of cats and rats, antitumoral, antifungal and antifertility activities.[2-3] Since 1948, *R. graveolens* has been reported to contain alkaloids, coumarins, flavonoids, lignans and phenolic acids.[4-6] The constituents of *R. graveolens* were further studied not only because of interest in the chemistry of natural products, but also because of several biologically active compounds providing a base for the use of *R. graveolens* in folk medicine and to find some more biologically active compounds.

In our continuing studies on the antiplatelet aggregation principles, we found that the methanol extract of the roots and aerial parts of *R. graveolens* showed strong antiplatelet and cytotoxicity activities. These results led us to reinvestigate the chemical constituents of the methanol extract of *R. graveolens*, which resulted in the isolation and characterization of nineteen compounds. This paper deals with the structural elucidation and antiplatelet aggregation and cytotoxicity.

## RESULTS AND DISCUSSIONS

The methanol extract of the roots of *R. graveolens* exhibited 65.4% and 81.1% inhibition of platelet aggregation activity induced by arachidonic acid (100 μM) and collagen (10 μg/mL), respectively. The methanol extract of the aerial parts of *R. graveolens* exhibited antiplatelet aggregation activity induced by collagen (10 μg/mL).

The chloroform layer of the roots, and the *n*-hexane and chloroform layers of the aerial parts exhibited antiplatelet aggregation induced by arachidonic acid (100 μM) and collagen (10 μg/mL) (Table 1). Subsequent bioassay-guided fractionation resulted in the isolation of nineteen known compounds. Compounds **1-4** showed typical UV and IR absorptions of 9-acridone alkaloids.[7] The $^1$H NMR of **1** exhibited signals corresponding to four mutually coupled protons at δ 8.44

Dedicated to Professor Fa-Ching Chen on the occasion of his ninetieth birthday.

* Corresponding author. Tel: +886-6-2747538; fax: +886-6-2740552; e-mail: tswu@mail.ncku.edu.tw

Table 1. Effect of Crude Extract and Partition Layers from *R. graveolens* on the Aggregation of Washed Rabbit Platelets Induced by Thrombin (Thr), Arachidonic Acid (AA), Collagen and PAF (% inhibition)

| Inducer<br>layers (200 μg/mL) | AA<br>100 μM | Col<br>10 μg/mL | Thr<br>0.1 U/mL | PAF<br>2 ng/mL |
|---|---|---|---|---|
| control | 0.0 ± 2.2 | 0.0 ± 1.9 | 0.0 ± 1.4 | 0.0 ± 1.9 |
| RM | 65.4 ± 15.0 | 81.1 ± 3.9 | 0.2 ± 0.3 | 0.2 ± 0.1 |
| RC | 85.8 ± 9.4 | 86.6 ± 0.0 | 4.2 ± 0.2 | 3.9 ± 0.9 |
| RW | 3.5 ± 1.1 | 1.9 ± 0.2 | -0.4 ± 0.4 | 0.2 ± 0.0 |
| LM | 19.6 ± 8.0 | 45.8 ± 8.3 | 1.9 ± 0.1 | 2.6 ± 0.7 |
| LH | 78.6 ± 15.2 | 75.2 ± 0.3 | 4.4 ± 0.2 | 8.8 ± 0.8 |
| LC | 95.5 ± 1.4 | 95.0 ± 0.3 | 2.5 ± 0.4 | 15.7 ± 2.0 |
| LW | 3.5 ± 1.5 | 2.8 ± 0.1 | 0.7 ± 0.4 | 0.3 ± 0.9 |

RM: the methanol extract of root, RC: the chloroform layer of root, RW: the water layer of root, LM: the methanol extract of aerial parts, LH: the hexane layer of aerial parts, LC: the chloroform layer of aerial parts, LW: the water layer of aerial parts.

(1H, dd, $J$ = 8.4, 1.6 Hz, H-8), 7.28 (1H, dd, $J$ = 8.4, 6.8 Hz, H-7), 7.73 (1H, ddd, $J$ = 8.4, 6.8, 1.6 Hz, H-6), and 7.50 (1H, d, $J$ = 8.4 Hz, H-5), three mutually coupled protons at δ 6.84 (1H, d, $J$ = 8.4 Hz, H-4), 7.55 (1H, dd, $J$ = 8.4, 8.4 Hz, H-3), 6.67 (1H, d, $J$ = 8.4 Hz, H-2), a chelated hydroxyl proton at δ 15.0 and a *N*-methyl at δ 3.89 (s). These data allowed the identification of **1** as 1-hydroxy-*N*-methylacridone.[8] Compound **2** showed one singlet at δ 6.27 (H-2), and two methoxyls at δ 4.19 and 4.15, instead of three mutually coupled protons of **1**. Hence, **2** was identified as arborinine.[9] The $^1$H NMR of **3** displayed a set of aliphatic proton signals at δ 5.11 (1H, m, H-2′), 5.10 (1H, m, H-4′), 4.95 (1H, m, H-4′), 3.59 (2H, m, H-1′), and 1.78 (3H, s, $CH_3$) attributable to an isopropenyl dihydrofuran moiety, and a singlet at δ 6.09 (H-2), instead of three mutually coupled protons of **1**. Thus, **3** was identified as rutacridone.[10] Compound **4** exhibited similar spectral data with that of **3** except for a multiplet for oxygenated methylene protons at δ 3.69 instead of terminal methylene of isopropenyl dihydrofuran moiety suggesting that **4** was an epoxide of **3**.[11]

Compounds **5-7** exhibited characteristic spectral data of furoquinoline alkaloids. On the basis of spectral comparisons with literature values, **5-7** were identified as common furoquinoline alkaloids, dictamine (**5**),[8] kokusaginine (**6**),[11] and skimmianine (**7**),[8] respectively. Four furocoumarins **8-11**, were identified as psoralen,[12] bergapten,[12] xanthotoxin,[12] and isopimpinellin,[13] respectively, by comparison of spectral data with those reported in the literature. Compounds **12-13** showed typical furocoumarin absorptions in their UV and IR spectra. In $^1$H NMR of **12**, three singlets at δ 7.68, 7.65, 7.43 were assigned to H-4, H-5, and H-8 of coumarin skeleton, respectively. A doublet at δ 7.67 ($J$ = 2.1 Hz) and a doublet of doublet at δ 6.82 ($J$ = 2.1, 0.9 Hz) corresponding to H-2′ and H-3′ of furan ring were also observed. In addition, signals due to 1,1-dimethyl-2-propenyl side chain attachment were found at δ 6.21 (1H, dd, $J$ = 17.7, 10.3 Hz, H-2″), 5.15 (1H, d, $J$ = 0.8 Hz, H-3″), 5.08 (1H, dd, $J$ = 5.8, 0.8 Hz, H-3″), and 1.52 (6H, s, 4″, 5″-$CH_3$). These data were identical with those of chalepensin.[3] The $^1$H NMR spectral data of **13** was similar to that of **12** except signals for the 2,2-dimethylcyclopropyl side chain at δ 1.87 (1H, t, $J$ = 5.1 Hz, H-1″), 0.85 (1H, dd, $J$ = 8.0, 5.1 Hz, H-3″), 0.78 (1H, dd, $J$ = 10.8, 5.1 Hz, H-3″), and 1.31 & 0.91 (each 3H, s, 4″, 5″-$CH_3$), instead of 1,1-dimethyl-2-propenyl subunit.[14] The spectral data of compound **14** inferred that it possessed a coumarin skeleton with a 1,1-dimethyl-2-propenyl side chain attachment as **12**. The $^1$H NMR spectra of **14** also showed signals attributable to 2-(1-acetyl-1-methylethyl)dihydrofuran substituent at δ 5.07 (1H, t, $J$ = 6.8 Hz, H-2′), 3.24 (1H, dd, $J$ = 16.0, 9.2 Hz, H-1′), 3.15 (1H, dd, $J$ = 15.6, 7.2 Hz, H-1′), 1.98 (3H, s, $CH_3$), 1.59 (3H, s, 5′-$CH_3$), and 1.53 (3H, s, 4′-$CH_3$). The above spectral data coincided well with the data of rutamarin.[15]

In addition, compound **15** was identified as a common coumarin, scopoletin, by comparison with an authentic sample.[8] The UV and IR data suggested that compound **16** should be a 2-arylquinoline alkaloid. The $^1$H NMR spectra revealed four mutually coupled protons at δ 7.67 (1H, dd, $J$ = 8.8, 1.5 Hz, H-8), 8.13 (1H, ddd, $J$ = 8.8, 8.8, 1.2 Hz, H-7), 7.45 (1H, ddd, $J$ = 8.8, 8.2, 1.5 Hz, H-6), and 7.71 (1H, dd, $J$ = 8.2, 1.2 Hz, H-5), a singlet at δ 7.07 (H-3), and a methoxyl singlet at δ 4.09 (4-$OCH_3$) for 4-methoxyquinoline skeleton, and a set of ABX pattern signals at δ 7.61 (1H, dd, $J$ = 8.1, 1.7 Hz, H-6′), 6.93 (1H, d, $J$ = 8.1 Hz, H-5′), and 7.67 (1H, d, $J$ = 1.7 Hz, H-2′) and a methylenedioxy singlet at δ 6.08 for 2-aryl

C.E.P.S.

substituent. From these data **16** was identified as graveolinine.[3] The UV and IR data of compound **17** was characteristic of a quinolone alkaloid. In addition to four mutually coupled protons, a set of ABX protons, and a methylenedioxy singlet as in **16**, the $^1$H NMR spectrum of **17** also showed a *N*-methyl singlet at δ 3.70 and an upfield shift of H-3 to δ 6.49. This data is identical with that reported for graveoline.[16] Moreover, a common sterol, β-sitosterol (**18**),[17] and a benzenoid, safrole (**19**),[18] were also identified by comparison with authentic samples.

The results of antiplatelet aggregation activity of some isolates are summarized in Table 2. The furoquinolines dictamine (**5**) and skimmianine (**7**) showed inhibitory activity on platelet aggregation induced by arachidonic acid (100 μM) and collagen (10 μg/mL). The furocoumarins psoralen (**8**), chalepensin (**12**), and clausindin (**13**) exhibited inhibitory activity on platelet aggregation induced by AA and collagen. The quinoline alkaloid graveolinine (**16**) exhibited the most potent antiplatelet aggregation activity. It showed 71.2% and 72.6% of inhibition of platelet aggregation induced by

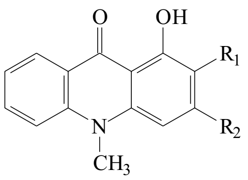

**1** $R_1 = R_2 = H$
**2** $R_1 = R_2 = OCH_3$

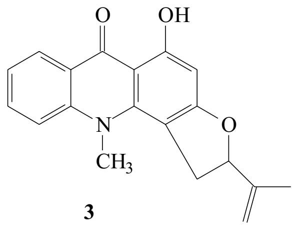

**3**

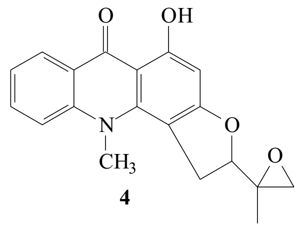

**4**

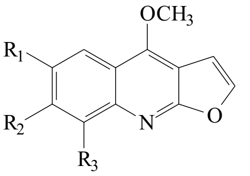

**5** $R_1 = R_2 = R_3 = H$
**6** $R_1 = R_2 = OCH_3$, $R_3 = H$
**7** $R_1 = H$, $R_2 = R_3 = OCH_3$

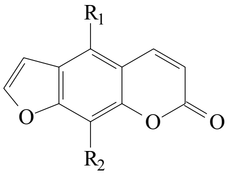

**8** $R_1 = R_2 = H$
**9** $R_1 = OCH_3$ , $R_2 = H$
**10** $R_1 = H$ , $R_2 = OCH_3$
**11** $R_1 = R_2 = OCH_3$

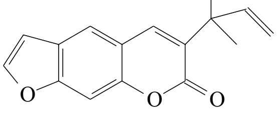

**12**

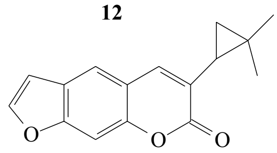

**13**

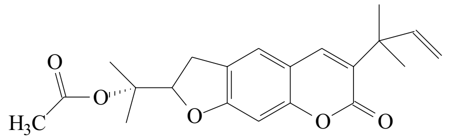

**14**

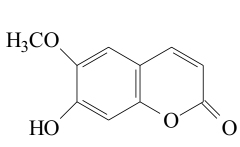

**15**

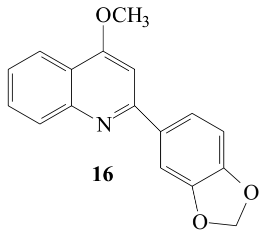

**16**

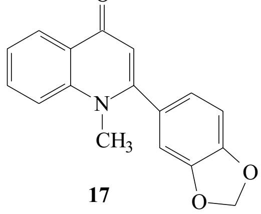

**17**

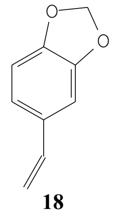

**18**

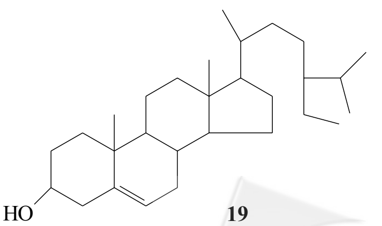

**19**

Table 2. Effect of Compounds Isolated from *R. graveolens* on the Aggregation of Washed Rabbit Platelets Induced by Thrombin (Thr), Arachidonic Acid (AA), Collagen and PAF (% inhibition)

| Inducer Compounds | Conc. (μg/mL) | AA 100 μM | Col 10 μg/mL | Thr 0.1 U/mL | PAF 2 ng/mL |
|---|---|---|---|---|---|
| control | | 0.0 ± 0.7 | 0.0 ± 0.3 | 0.0 ± 0.9 | 0.0 ± 0.6 |
| **1** | 50 | A | | | |
| | 20 | 17.8 ± 3.7$^{+++}$ | 3.7 ± 1.3$^{+}$ | 4.3 ± 1.3 | 3.4 ± 0.0$^{++}$ |
| **2** | 50 | 51.6 ± 4.7$^{+++}$ | 39.1 ± 8.5$^{+++}$ | 1.1 ± 0.6 | 4.8 ± 0.5$^{+++}$ |
| **5** | 100 | 100 ± 0.7$^{+++}$ | 84.4 ± 6.2$^{+++}$ | 1.3 ± 0.3 | 12.6 ± 0.3$^{+++}$ |
| | 50 | 69.8 ± 11.8$^{+++}$ | 44.4 ± 17.7$^{++}$ | | |
| | 20 | 22.5 ± 1.2$^{+++}$ | 4.3 ± 2.0$^{+}$ | | |
| | 10 | 8.7 ± 0.1$^{+++}$ | | | |
| **6** | 100 | 42.2 ± 6.0$^{+++}$ | 53.1 ± 17.4$^{++}$ | 0.9 ± 0.0 | 3.5 ± 0.3$^{+}$ |
| **7** | 100 | 81.5 ± 9.5$^{+++}$ | 45.9 ± 17.4$^{++}$ | | |
| | 50 | 13.1 ± 2.7$^{++}$ | 7.8 ± 2.7$^{++}$ | | |
| | 20 | 5.4 ± 0.6$^{++}$ | 2.5 ± 1.2 | | |
| **8** | 100 | 86.7 ± 10.2$^{+++}$ | 74.8 ± 6.5$^{+++}$ | 2.1 ± 0.9$^{+++}$ | 5.7 ± 0.2$^{+++}$ |
| | 50 | 63.4 ± 11.3$^{+++}$ | 15.8 ± 2.1$^{+++}$ | | |
| | 20 | 17.4 ± 2.1$^{+++}$ | 5.1 ± 2.1$^{+}$ | | |
| | 10 | 10.4 ± 0.4$^{+++}$ | | | |
| **9** | 50 | 63.5 ± 5.6$^{+++}$ | 59.0 ± 12.1$^{+++}$ | 7.5 ± 1.1$^{++}$ | 4.5 ± 1.2$^{++}$ |
| **10** | 50 | 18.5 ± 2.6$^{+++}$ | 8.4 ± 0.4$^{+++}$ | 0.6 ± 0.0 | 4.3 ± 0.1$^{+++}$ |
| **11** | 100 | 30.4 ± 0.3$^{+++}$ | 8.0 ± 0.9$^{+++}$ | 3.9 ± 1.0 | 4.5 ± 0.8$^{++}$ |
| **12** | 100 | 100.0 ± 1.1$^{+++}$ | 100.0 ± 0.3$^{+++}$ | 12.7 ± 2.4$^{++}$ | 52.0 ± 1.7$^{+++}$ |
| | 50 | 100.0 ± 1.1$^{+++}$ | 97.0 ± 2.1$^{+++}$ | | 13.3 ± 0.5$^{+++}$ |
| | 20 | 44.9 ± 9.1$^{+++}$ | 28.5 ± 11.2$^{++}$ | | |
| | 10 | 10.4 ± 0.6$^{+++}$ | 5.8 ± 1.5$^{++}$ | | |
| | 5 | 7.3 ± 0.2$^{+++}$ | | | |
| **13** | 100 | 89.2 ± 3.5$^{+++}$ | 73.9 ± 7.4$^{+++}$ | 7.9 ± 0.1$^{+++}$ | 12.8 ± 1.1$^{+++}$ |
| | 50 | 68.4 ± 15.4$^{+++}$ | | | |
| | 20 | 22.2 ± 6.9$^{++}$ | | | |
| | 10 | 7.2 ± 1.1$^{++}$ | | | |
| **14** | 50 | 7.6 ± 0.9$^{+++}$ | 10.5 ± 1.6$^{+++}$ | 3.0 ± 0.1 | 4.5 ± 0.3$^{++}$ |
| **15** | 100 | 14.7 ± 1.8$^{+++}$ | 3.4 ± 0.5$^{+++}$ | 1.5 ± 1.1$^{++}$ | 1.6 ± 0.2 |
| **16** | 100 | 100.0 ± 0.7$^{+++}$ | 100.0 ± 0.3$^{+++}$ | 9.0 ± 1.3$^{++}$ | 34.6 ± 11.7$^{+}$ |
| | 50 | 100.0 ± 0$^{+++}$· | 81.2 ± 15.1$^{+++}$ | | |
| | 20 | 77.4 ± 10.7$^{+++}$ | 72.6 ± 7.8$^{+++}$ | | |
| | 10 | 73.1 ± 11.3$^{+++}$ | 49.5 ± 8.2$^{+++}$ | | |
| | 5 | 71.2 ± 9.2$^{+++}$ | 33.3 ± 8.4$^{+++}$ | | |
| | 2 | 38.4 ± 7.8$^{+++}$ | 25.4 ± 7.1$^{+++}$ | | |
| | 1 | 26.9 ± 8.4$^{++}$ | 19.2 ± 5.4$^{+++}$ | | |
| | 0.5 | 14.9 ± 4.7$^{+}$ | 5.8 ± 1.9$^{++}$ | | |
| | 0.2 | 6.8 ± 0.8$^{++}$ | | | |
| **17** | 50 | 9.5 ± 0.3$^{+++}$ | 5.2 ± 0.9$^{+++}$ | 1.1 ± 0.2 | 6.2 ± 1.1$^{+++}$ |

Platelets were preincubated with DMSO (0.5%, control) at 37 °C for 3 mim; the inducer was then added. Values are means ± SEM (n = 3~5).

A: Platelet aggregation was promoted.

$^{+}P < 0.05$, $^{++}P < 0.01$, $^{+++}P < 0.001$ as compared with respective control.

arachidonic acid and collagen at 5 μg/mL and 20 μg/mL, respectively. 1-Hydroxy-*N*-methylacridone (**1**), arborinine (**2**), kokusaginine (**6**), bergapten (**9**), xanthotoxin (**10**), isopimpinellin (**11**), rutamarin (**14**), scopoletin (**15**), and graveoline (**17**), all showed no obvious activity at 100 μg/mL.

The compounds were also tested for their cytotoxicity against KB, Hela, DLD, CI, and Hepa tumor cell lines. The results ($ED_{50}$ values) are summarized in Table 3. The acridone alkaloid 1-hydroxy-*N*-methylacridone (**1**) showed cytotoxic effect against KB tumor cell lines; the furoquino-

Table 3. Cytotoxicity of Compounds Isolated from *R. graveolens* on Tumor Cell Lines

| | cell line $ED_{50}$ (μg/mL) | | | | |
|---|---|---|---|---|---|
| Compounds | KB | Hela | DLD | NCI | Hepa |
| **1** | 4.6 | 10.61 | 8.51 | 7.01 | - |
| **2** | 12.36 | 11.90 | - | - | - |
| **5** | 20.55 | 2.5 | 6.4 | - | - |
| **6** | 7.48 | 3.9 | - | - | - |
| **7** | - | - | - | - | - |
| **8** | - | - | - | - | - |
| **9** | 8.89 | - | - | - | - |
| **10** | - | - | - | - | - |
| **11** | 3.21 | 1.30 | 4.06 | 1.53 | 4.47 |
| **12** | -* | -* | -* | -* | -* |
| **13** | - | -* | -* | - | 3.24 |
| **14** | - | 18.06 | 13.34 | - | - |
| **15** | - | - | - | - | - |
| **16** | - | - | - | - | - |
| **17** | - | 3.35 | - | - | - |

DLD (DLD-1): Human colon adenocarcinoma
Hela: Human cervix epithelioid carcinoma
KB: Human oral epidermoid carcinoma
NCI (NCI-H661): Human lung large cell carcinoma
Hepa: Human hepatoma
*: enhance the activity of mitochondria of tumor cells
"-": $ED_{50}$ > 20 μg/mL
Standard doxorubicin: $ED_{50}$ ≤ 0.1 μg/mL

lines dictamine (**5**), and kokusaginine (**6**) against Hela tumor cell lines; the furocoumarins isopimpinellin (**11**) against KB, DLD, NCI and Hepa tumor cell lines; clausindin (**13**) against Hepa tumor cell lines; and the quinoline alkaloid graveoline (**17**) against Hela tumor cell lines.

## EXPERIMENTAL SECTION

### General Experimental Procedures

Silica gel 60 (Merck 70-230, 230-400 mesh), was used for column chromatography. Glass sheets of precoated silica gel 60 $F_{254}$ (Merck 0.2 mm thick) were used for TLC. Melting points were measured on a Yanagimoto MP-S3 micromelting point apparatus and were uncorrected. The UV spectra were recorded on a Hitachi UV-3210 spectrophotometer and IR spectra were determined as KBr discs on a Shimadzu FTIR-8501 spectrophotometer. The NMR spectra were recorded on Bruker AC-200, Avance 300 and Varian Unity Plus-400 spectrometers. Chemical shifts were shown in δ values with tetramethylsilane as internal standard. The mass spectra were performed in the EI mode on a VG 70-250S spectrometer. The optical rotations were measured on a JASCO DIP-370 polarimeter.

### Plant Materials

The root and aerial parts of *R. graveolens* were collected from Taichung, Taiwan, and identified by Prof. C. S. Kuoh. A voucher specimen (Wu 950012) was deposited in the Herbarium of National Cheng Kung University, Tainan, Taiwan.

### Extraction and Isolation

Fresh root and aerial parts of *R. graveolens* (each 10 kg) were cut into small pieces then were extracted with methanol and concentrated under reduced pressure. Methanol extract of root was partitioned with $CHCl_3$ and that of the aerial parts was treated with *n*-hexane and $CHCl_3$, successively. The $CHCl_3$ layer (120 g) of root was concentrated under reduced pressure to leave brown syrup which was directly chromatographed over silica gel and eluted with a gradient of *n*-hexane and acetone to give seven fractions. Each fraction was rechromatographed over silica gel and then subjected to TLC or recrystallization. Fraction 1 was rechromatographed over silica gel using EtOAc-hexane (1:9) as an eluent to obtain **1** (58.3 mg), **9** (16.0 mg), **10** (52.2 mg), **11** (74.8 mg), **16** (48.5 mg) and **18** (4.8 mg), respectively. Fraction 2 was repeatedly chromatographed over silica gel eluted with $Me_2CO$-$C_6H_6$ (1:25) to give **14** (34.2 mg).

The *n*-hexane layer (110 g) of aerial parts was concentrated under reduced pressure to leave brown syrup which was directly chromatographed over silica gel and eluted with a gradient of *n*-hexane and acetone to give eleven fractions. Each fraction was rechromatographed over silica gel and subjected to TLC or recrystallization. Fraction 3 was rechromatographed over silica gel using diisopropyl ether-hexane (1:20) as an eluent to obtain **12** (32.5 mg). Fraction 4 was repeatedly chromatographed over silica gel eluted with diisopropyl ether-$C_6H_6$ (1:20) to afford **12** (104.1 mg) and **13** (15.0 mg), fraction 7 was rechromatographed over silica gel using $Me_2CO$-$C_6H_6$ (1:25) as an eluent to obtain **16** (26.4 mg), fraction 8 was repeatedly chromatographed over silica gel eluted with $Me_2CO$-$C_6H_6$ (1:20) to give **9** (39.5 mg), fraction 9 was repeatedly chromatographed over silica gel eluted with EtOAc-$C_6H_6$ (1:9) to give **1** (33.1 mg), **5** (11.3 mg), **8** (27.5 mg), **9** (73.6 mg) and **19** (32.6 mg), fraction 10 was repeatedly chromatographed over silica gel eluted with $Me_2CO$-hexane (1:9) to obtain **1** (12.2 mg), **9** (83.7 mg), **11** (10.6 mg) and unknown **A** (0.5 mg) and fraction 11 was repeatedly chromatographed over silica gel eluted with $Me_2CO$-hexane (1:9) to afford **2** (80.0 mg), **3** (7.2 mg), **6** (94.5 mg), **9** (14.3 mg) and **11** (16.0 mg), successively.

C.E.P.S.

The $CHCl_3$ layer (80 g) of aerial parts was concentrated under reduced pressure to leave brown syrup, which was directly chromatographed over silica gel and eluted with a gradient of *n*-hexane and acetone to give seven fractions. On rechromatography with $Me_2CO$-hexane (1:20) eluent, fraction 1 afforded **8** (11.2 mg), **9** (106.6 mg), **10** (52.6 mg), **12** (45.8 mg), **13** (230.5 mg) and **14** (13.3 mg) and fraction 2 was eluted with $Me_2CO$-hexane (1:9) eluent gave **2** (27.0 mg), **4** (6.8 mg), **6** (31.4 mg), **7** (42.9 mg), **12** (10.0 mg), **15** (7.3 mg), **17** (602.8 mg) and **19** (34.2 mg), successively.

**1-Hydroxy-10-methylacridone (1)**

Yellow needles; Mp 131-132 °C; UV (MeOH) (log ε) $\lambda_{max}$/nm: 313, 263, 257 (sh), 243 (sh), 216; IR (KBr) $\nu_{max}$/cm$^{-1}$: 2918, 2849, 1630, 1593, 1501, 1468, 759, 671; EIMS *m/z* (%): 225 ([M]$^+$, 100), 197 (8), 182 (9), 154 (6), 127 (5), 98 (4), 77 (6); $^1$H-NMR (400 MHz, $CDCl_3$): δ 8.44 (1H, dd, *J* = 8.4, 1.6 Hz, H-8), 7.73 (1H, ddd, *J* = 8.4, 6.8, 1.6 Hz, H-6), 7.55 (1H, dd, *J* = 8.4, 8.4 Hz, H-3), 7.50 (1H, d, *J* = 8.4 Hz, H-5), 7.28 (1H, dd, *J* = 8.4, 6.8 Hz, H-7), 6.84 (1H, d, *J* = 8.4 Hz, H-4), 6.67 (1H, d, *J* = 8.4 Hz, H-2), 3.89 (3H, s, N-$CH_3$); $^{13}$C-NMR ($CDCl_3$, 100 MHz): δ 181.9 (C-9), 163.6 (C-1), 143.2 (C-10a), 142.2 (C-4a), 135.7 (C-6), 126.5 (C-8), 121.2 (C-7), 120.9 (C-8a), 114.5 (C-5), 109.8 (C-9a), 107.4 (C-4), 103.4 (C-2), 33.8 (N-$CH_3$).

**Arborinine (2)**

Yellow needles; Mp 175-177 °C; UV (MeOH) $\lambda_{max}$/nm: 293 (sh), 274, 264 (sh), 246 (sh), 230; IR (KBr) $\nu_{max}$/cm$^{-1}$: 1639, 1589, 1518, 1471, 1354, 1317, 1281, 1254, 1184; EIMS *m/z* (%): 285 ([M]$^+$, 76), 270 (100), 242 (27), 199 (17); $^1$H-NMR (300 MHz, $CDCl_3$): δ 15.0 (1H, br. s, $D_2O$ exchangeable, OH), 8.43 (1H, d, *J* = 8.0 Hz, H-8), 7.86 (1H, dd, *J* = 8.8, 7.4 Hz, H-6), 7.59 (1H, d, *J* = 8.8 Hz, H-5), 7.39 (1H, dd, *J* = 8.0, 7.4 Hz, H-7), 6.27 (1H, s, H-2), 4.19 (3H, s, $OCH_3$), 4.15 (3H, s, $OCH_3$), 3.88 (3H, s, N-$CH_3$).

**Rutacridone (3)**

Yellow needles; Mp 145-147 °C; UV (MeOH) $\lambda_{max}$/nm: 300, 273, 245, 225; IR (KBr) $\nu_{max}$/cm$^{-1}$: 3420, 1635, 1589, 1518, 1325, 1317; EIMS *m/z* (%) : 307 ([M]$^+$, 100), 292 (38), 278 (22), 264 (22), 250 (13), 239 (17), 236 (15), 211 (12), 180 (7), 146 (9), 107 (12), 89 (8), 77(24); $^1$H-NMR (200 MHz, $CDCl_3$): δ 15.20 (1H, s, $D_2O$ exchangeable, OH), 8.21 (1H, ddd, *J* = 8.0, 1.2, 0.6 Hz, H-8), 7.58 (1H, m, H-6), 7.23 (1H, br. d, *J* = 8.4 Hz, H-5), 7.13 (1H, m, H-7), 6.09 (1H, s, H-2), 5.11 (1H, m, H-2′), 5.10 (1H, m, H-4′), 4.95 (1H, m, H-4′), 3.80 (3H, s, $NCH_3$), 3.59 (2H, m, H-1′), 1.78 (3H, s, $CH_3$).

**Rutacridon-epoxide (4)**

Yellowish needles; Mp 153-155 °C; UV (MeOH) $\lambda_{max}$/nm: 290, 274, 246 (sh), 230; IR (KBr) $\nu_{max}$/cm$^{-1}$: 3325, 1639, 1528, 1474, 1468; EIMS *m/z* (%): 323 ([M]$^+$, 100), 292 (99), 277 (64), 265 (42), 250 (21), 241 (33), 225 (21), 212 (17); $^1$H-NMR (200 MHz, $CDCl_3$): δ 14.85 (1H, s, $D_2O$ exchangeable, OH), 8.38 (1H, dd, *J* = 7.9, 1.3 Hz, H-8), 7.71 (1H, ddd, *J* = 8.8, 7.4, 1.3 Hz, H-6), 7.40 (1H, d, *J* = 8.8 Hz, H-5), 7.29 (1H, dd, *J* = 7.9, 7.4 Hz, H-7), 6.22 (1H, s, H-2), 4.94 (1H, t, *J* = 8.9 Hz, H-2′), 3.99 (3H, s, N-$CH_3$), 3.69 (4H, m, H-1′, 5′), 1.39 (3H, s, H-4′).

**Dictamine (5)**

Colorless powder; Mp 126-128 °C; UV (MeOH) $\lambda_{max}$/nm: 330, 308, 241 (sh), 237; IR (KBr) $\nu_{max}$/cm$^{-1}$: 1624, 1582, 1508, 1375, 1298, 1209, 1119, 1086, 980; EIMS *m/z* (%): 199 ([M]$^+$, 100), 184 (49), 156 (27), 128 (13); $^1$H-NMR (200 MHz, $CDCl_3$): δ 8.27 (1H, dd, *J* = 8.3, 1.3 Hz, H-8), 8.02 (1H, dd, *J* = 8.0, 1.2 Hz, H-5), 7.68 (1H, ddd, *J* = 8.0, 6.8, 1.3 Hz, H-6), 7.62 (1H, d, *J* = 2.7 Hz, H-2′), 7.44 (1H, ddd, *J* = 8.3, 6.8, 1.0 Hz, H-7), 7.07 (1H, d, *J* = 2.7 Hz, H-1′), 4.44 (3H, s, 4-$OCH_3$).

**Kokusaginine (6)**

Colorless powder; Mp 171-172 °C; UV (MeOH) $\lambda_{max}$/nm: 334, 321, 308, 251, 244, 214; IR (KBr) $\nu_{max}$/cm$^{-1}$: 2997, 2951, 1622, 1587, 1506, 1481, 1427, 1367, 1321, 1258, 1211, 1161; EIMS *m/z* (%): 259 ([M]$^+$, 100), 244 (34), 216 (11), 201 (8), 186 (9), 173 (6); $^1$H-NMR (400 MHz, $CDCl_3$): δ 7.58 (1H, d, *J* = 2.8 Hz, H-2′), 7.48 (1H, s, H-5), 7.34 (1H, s, H-8), 7.05 (1H, d, *J* = 2.8 Hz, H-1′), 4.45 (3H, s, 4-$OCH_3$), 4.03 (3H, s, 7-$OCH_3$), 4.02 (3H, s, 6-$OCH_3$); $^{13}$C-NMR ($CDCl_3$, 100 MHz): δ 162.9, 155.5, 152.5, 147.7, 142.3, 112.8, 106.5, 104.5, 102.1, 100.1, 58.7, 55.9, 55.9.

**Skimmianine (7)**

Colorless powder; Mp 179-181 °C; UV (MeOH) $\lambda_{max}$/nm: 3331, 320, 249, 241 (sh); IR (KBr) $\nu_{max}$/cm$^{-1}$: 3117, 3009, 2977, 1618, 1576, 1549, 1447, 1389, 1362, 1265, 1236, 1090, 991; EIMS *m/z* (%) : 259 ([M]$^+$, 100), 244 (84), 230 (33), 216 (25), 213 (15), 201 (19), 199 (13), 173 (10), 130 (10); $^1$H-NMR (200 MHz, $CDCl_3$): 8.01 (1H, d, *J* = 9.4 Hz, H-5), 7.58 (1H, d, *J* = 2.9 Hz, H-2′), 7.23 (1H, d, *J* = 9.4 Hz, H-6), 7.04 (1H, d, *J* = 2.9 Hz, H-1′), 4.43 (3H, s, 4-$OCH_3$), 4.11 (3H, s, 8-$OCH_3$), 4.03 (3H, s, 7-$OCH_3$).

**Psoralen (8)**

Colorless needles; Mp 162-163 °C; UV (MeOH) $\lambda_{max}$/

nm: 325 (sh), 296, 267 (sh), 247, 241 (sh), 224 (sh), 209; IR (KBr) $\nu_{max}$/cm$^{-1}$: 1720, 1628, 1576, 1281, 1128, 1016, 889, 822, 752; EIMS *m/z* (%): 186 ([M]$^+$, 100), 173 (13), 158 (79), 130 (11), 102 (22), 51 (18); $^1$H-NMR (200 MHz, $CDCl_3$): δ 7.81 (1H, d, *J* = 9.6 Hz, H-4), 7.70 (1H, d, *J* = 2.4 Hz, H-2′), 7.69 (1H, s, H-5), 7.48 (1H, s, H-8), 6.76 (1H, dd, *J* = 2.4, 0.9 Hz, H-3′), 6.38 (1H, d, *J* = 9.6 Hz, H-3).

**Bergapten (9)**

Colorless needles; Mp 189-190 °C; UV (MeOH) $\lambda_{max}$/nm: 307, 268, 257, 247, 241 (sh), 221; IR (KBr) $\nu_{max}$/cm$^{-1}$: 2924, 2853, 1734, 1578, 1541, 1470, 1358, 1126, 752; EIMS *m/z* (%): 216 ([M]$^+$, 100), 201 (24), 186 (27), 173 (39), 158 (17), 145 (15); $^1$H-NMR (200 MHz, $CDCl_3$): δ 8.15 (1H, d, *J* = 9.8 Hz, H-4), 7.59 (1H, d, *J* = 2.4 Hz, H-2′), 7.13 (1H, s, H-8), 7.01 (1H, d, *J* = 2.4 Hz, H-3′), 6.27 (1H, d, *J* = 9.8 Hz, H-3).

**Xanthotoxin (10)**

Colorless needles; Mp 147-149 °C; UV (MeOH) $\lambda_{max}$/nm: 304, 263, 247, 243, 219; IR (KBr) $\nu_{max}$/cm$^{-1}$: 3121, 2999, 2949, 1740, 1622, 1587, 1471, 1460, 1400, 1333, 1148, 1097; EIMS *m/z* (%): 216 ([M]$^+$, 100), 201 (21), 188 (11), 173 (33), 145 (12), 89 (13); $^1$H-NMR (200 MHz, $CDCl_3$): δ 7.77 (1H, d, *J* = 9.6 Hz, H-4), 7.69 (1H, d, *J* = 2.1 Hz, H-2′), 7.35 (1H, s, H-8), 6.82 (1H, d, *J* = 2.1 Hz, H-3′), 6.37 (1H, d, *J* = 9.6 Hz, H-3).

**Isopimpinellin (11)**

Colorless needles; Mp 186-187 °C; UV (MeOH) $\lambda_{max}$/nm: 303, 263 (sh), 248, 243, 219; IR (KBr) $\nu_{max}$/cm$^{-1}$: 1720, 1597, 1477, 1427, 1356, 1142, 1069, 818, 748; EIMS *m/z* (%): 246 ([M]$^+$, 100), 231 (81), 203 (10); $^1$H-NMR (200 MHz, $CDCl_3$): δ 8.13 (1H, d, *J* = 9.8 Hz, H-4), 7.63 (1H, d, *J* = 2.4 Hz, H-2′), 7.00 (1H, d, *J* = 2.4 Hz, H-3′), 6.30 (1H, d, *J* = 9.8 Hz, H-3), 4.17 (6H, s, 5, 8-$OCH_3$).

**Chalepensin (12)**

Colorless needles; Mp 82-83 °C; UV (MeOH) $\lambda_{max}$/nm: 326, 291, 246, 241 (sh), 206; IR (KBr) $\nu_{max}$/cm$^{-1}$: 3086, 3003, 2970, 2934, 2874, 1728, 1682, 1583, 1543, 1452, 1412, 1377, 1312, 1285, 1167, 1101, 993, 756; EIMS *m/z* (%): 254 ([M]$^+$, 100), 239 (80), 225 (12), 211 (52), 199 (50), 171 (17), 155 (24), 129 (15), 128 (15), 115 (14); $^1$H-NMR (200 MHz, $CDCl_3$): δ 7.68 (1H, s, H-4), 7.67 (1H, d, *J* = 2.1 Hz, H-2′), 7.65 (1H, s, H-5), 7.43 (1H, s, H-8), 6.82 (1H, dd, *J* = 2.1, 0.9 Hz, H-3′), 6.21 (1H, dd, *J* = 17.7, 10.3 Hz, H-2″), 5.15 (1H, d, *J* = 0.8 Hz, H-3″), 5.08 (1H, dd, *J* = 5.8, 0.8 Hz, H-3″), 1.52 (6H, s, 4″, 5″-$CH_3$).

**Clausindin (13)**

Colorless prisms; Mp 80-81 °C; UV (MeOH) $\lambda_{max}$/nm: 329 (sh), 300, 247, 243 (sh), 212, 204; IR (KBr) $\nu_{max}$/cm$^{-1}$: 3086, 3003, 2970, 2934, 2874, 1728, 1682, 1583, 1543, 1452, 1412, 1377, 1312, 1285, 1167, 1101, 993, 756; EIMS *m/z* (%): 254 ([M]$^+$, 100), 239 (80), 225 (12), 211 (52), 199 (50), 171 (17), 155 (24), 129 (15), 128 (15), 115 (14); $^1$H-NMR (200 MHz, $CDCl_3$): δ 7.67 (1H, d, *J* = 2.2 Hz, H-2′), 7.61 (1H, s, H-4), 7.47 (1H, s, H-5), 7.38 (1H, s, H-8), 6.81 (1H, d, *J* = 2.2 Hz, H-3′), 1.87 (1H, t, *J* = 5.1 Hz, H-1″), 1.31 & 0.91 (each 3H, s, 4″, 5″-$CH_3$), 0.85 (1H, dd, *J* = 8.0, 5.1 Hz, H-3″), 0.78 (1H, dd, *J* = 10.8, 5.1 Hz, H-3″).

**Rutamarin (14)**

Colorless prisms; Mp 107-109 °C; $[\alpha]_D$ +16.6° (*c* 0.48, MeOH); UV (MeOH) $\lambda_{max}$/nm: 334, 299 (sh), 259, 249, 223; IR (KBr) $\nu_{max}$/cm$^{-1}$: 3013, 2966, 2924, 2853, 1728, 1628, 1580, 1485, 1385, 1369, 1267, 1250, 1130, 989, 756; EIMS *m/z* (%): 356 ([M]$^+$, 28), 341 (12), 296 (19), 282 (19), 281 (100), 243 (10); $^1$H-NMR (400 MHz, $CDCl_3$): δ 7.48 (1H, s, H-4), 7.19 (1H, s, H-5), 6.71 (1H, s, H-8), 6.17 (1H, dd, *J* = 17.2, 10.4 Hz, H-2″), 5.08 (2H, d, *J* = 10.0 Hz, H-3″), 5.07 (1H, t, *J* = 6.8 Hz, H-2′), 3.24 (1H, dd, *J* = 16.0, 9.2 Hz, H-1′), 3.15 (1H, dd, *J* = 15.6, 7.2 Hz, H-1′), 1.98 (3H, s, $CH_3$), 1.59 (3H, s, 5′-$CH_3$), 1.53 (3H, s, 4′-$CH_3$), 1.50 (6H, s, 4″, 5″-$CH_3$); $^{13}$C-NMR (100 MHz, $CDCl_3$): δ 170 ($\underline{C}OCH_3$), 162.3 (C-7), 160.0 (C-2), 154.6 (C-8a), 145.5 (C-2″), 138.0 (C-4), 130.2 (C-3), 123.8 (C-6), 123.0 (C-5), 112.9 (C-4a), 119.4 (C-3″), 96.9 (C-8), 88.2 (C-2′), 82.1 (C-3′), 40.2 (C-1″), 29.6 (C-1′), 26.0 (C-4″, 5″), 22.2 ($CO\underline{C}H_3$), 21.8 (C-5′), 20.9 (C-4′).

**Scopoletin (15)**

Colorless needles; Mp 178-180 °C; UV (MeOH) $\lambda_{max}$/nm: 345, 298, 253, 229, 206; IR (KBr) $\nu_{max}$/cm$^{-1}$: 3339, 1719, 1609, 1570, 1514, 1296, 1263, 1149, 860; EIMS *m/z* (%): 192 ([M]$^+$, 100), 177 (68), 164 (32), 149 (54), 121 (22), 79 (18), 69 (35), 51 (18); $^1$H-NMR (200 MHz, $CDCl_3$): δ 7.60 (1H, d, *J* = 9.5 Hz, H-4), 6.91 (1H, s, H-5), 6.84 (1H, s, H-8), 6.26 (1H, d, *J* = 9.5 Hz, H-3), 6.18 (1H, br. s, $D_2O$ exchangeable, OH), 3.95 (3H, s, $OCH_3$).

**Graveolinine (16)**

Colorless powder; Mp 114-115 °C; UV (MeOH) $\lambda_{max}$/nm: 310, 300, 273, 233, 221 (sh); IR (KBr) $\nu_{max}$/cm$^{-1}$: 2918, 2851, 1618, 1591, 1558, 1504, 1450, 1418, 1373, 1246, 1113, 1040, 758; EIMS *m/z* (%): 279 ([M]$^+$, 100), 252 (13), 251 (82), 192 (11); $^1$H-NMR (200 MHz, $CDCl_3$): δ 8.13 (1H, ddd, *J* = 8.8, 8.8, 1.2 Hz, H-7), 7.71 (1H, dd, *J* = 8.2, 1.2 Hz, H-5),

C.E.P.S.

7.67 (1H, d, *J* = 1.7 Hz, H-2′), 7.67 (1H, dd, J = 8.8, 1.5 Hz, H-8), 7.61 (1H, dd, *J* = 8.1, 1.7 Hz, H-6′), 7.45 (1H, ddd, *J* = 8.8, 8.2, 1.5 Hz, H-6), 7.07 (1H, s, H-3), 6.93 (1H, d, *J* = 8.1 Hz, H-5′), 6.03 (2H, s, $OCH_2O$), 4.09 (3H, s, $OCH_3$).

**Graveoline (17)**

Colorless powder; Mp 214-216 °C; UV (MeOH) $\lambda_{max}$/nm: 337, 324, 314 (sh), 291, 273, 243, 213; IR (KBr) $\nu_{max}$/$cm^{-1}$: 2926, 1620, 1597, 1564, 1488, 1468, 1445, 1250, 1038, 760; EIMS *m/z* (%): 279 ($[M]^+$, 100), 278 (34), 250 (19), 249 (24), 191 (13), 178 (16); $^1$H-NMR (200 MHz, $CDCl_3$): δ 8.48 (1H, dd, *J* = 8.2, 1.7 Hz, H-5), 7.76 (1H, ddd, *J* = 8.5, 8.5, 1.6 Hz, H-7), 7.60 (1H, d, *J* = 8.5 Hz, H-8), 7.46 (1H, ddd, *J* = 8.5, 8.5, 0.9 Hz, H-6), 6.92 (1H, d, *J* = 1.7 Hz, H-2′), 6.89 (1H, d, *J* = 8.3 Hz, H-6′), 6.89 (1H, dd, *J* = 8.3, 1.7 Hz, H-5′), 6.49 (1H, s, H-3), 6.08 (2H, s, $OCH_2O$), 3.70 (3H, s, N-$CH_3$).

**Safrole (18)**

Colorless syrup; IR (KBr) $\nu_{max}$/$cm^{-1}$: 1687; $^1$H-NMR (200 MHz, $CDCl_3$): δ 6.67 (3H, m, H-5, 8, 9), 5.92 (2H, s, $OCH_2O$), 5.80 (1H, dd, *J* = 15.7, 6.7 Hz, H-2), 5.00 (1H, dd, *J* = 15.7, 5.2 Hz, H-1), 4.94 (1H, dd, *J* = 6.7, 5.2 Hz, H-1), 2.53 (2H, t, *J* = Hz, H-3).

**β-Sitosterol (18)**

Colorless powder; Mp 214-216 °C; IR (KBr) $\nu_{max}$/$cm^{-1}$: 3450, 1650, 1645, 1385; EIMS *m/z* (%): 414 ($[M]^+$, 100), 396 (34), 381 (15), 303 (24), 255 (22), 213 (17), 145 (22), 105 (25); $^1$H-NMR (200 MHz, $CDCl_3$): δ 5.35 (1H, d, *J* = 5.0 Hz, H-6), 3.53 (1H, m, H-3), 1.01 (3H, s, H-19), 0.91 (3H, d, *J* = 6.4 Hz, H-21), 0.85 (3H, t, *J* = 7.4 Hz, H-29), 0.83 (3H, d, *J* = 4.2 Hz, H-26), 0.81 (3H, d, *J* = 4.2 Hz, H-27), 0.68 (3H, s, H-18).

**Biological Assay**

Washed rabbit platelets were obtained from EDTA-anticoagulated platelet-rich plasma according to the method reported by Teng et al.[19] The platelet pellets were suspended in Tyrode's solution of the following composition (nM): NaCl (136.8), KCl (2.8), $NaHCO_3$ (11.9), $MgCl_2$ (2.1), $NaH_2PO_4$ (0.33), $CaCl_2$ (1.0), and glucose (11.2), containing bovine serum albumin (0.35%). Platelet aggregation was measured by the turbidimetric method reported by O'Brien.[20] Percentages of aggregation were calculated using the absorbance of platelet suspension to represent 0% aggregation and the absorbance of Tyrode's solution as 100% aggregation. The *in vitro* cytotoxicity assay was carried out according to the method reported by Eilliott et al.[21]

**ACKNOWLEDGMENT**

We thank the National Science Council, R. O. C. (NSC 90-2113-M-006-019) for support of this research.

Received October 3, 2002.

**REFERENCES**

1. Jiangsu New Medicine College in Encyclopedia of Chinese Material Media, Shanghai Science and Technology Press: Shanghai, 1977; Vol. 2, p 1887.
2. Novak, I.; Buzas, G.; Minker, E.; Koltai, M.; Szendrei, K. *Pharmazie* **1965**, *20*, 738, and therein.
3. Kong, Y. C.; Lau, C. P.; Wat, K. H.; Ng, K. H.; But, P. P. H.; Chen, K. F.; Waterman, P. G. *Plant Med.* **1989**, *55*, 176.
4. Reisch, J.; Rozsa, Z.; Szendrei, K.; Novak, I.; Minker, E. *Phytochemistry* **1976**, *15*, 240.
5. Hubert, P.; Reiner, W.; Jutta, K.; Oskar, S. *Planta Med.* **1991**, *57*, 82, and therein.
6. Zobel, A. M.; Brown, S. A. *J. Chem. Ecol.* **1991**, *17*, 1801, and therein.
7. Brown, R. D.; Lahey, F. N. *Aust. J. Sci. Res. Ser. A.* **1972**, *3*, 593.
8. Wu, T. S.; Li, C. Y.; Leu, Y. L.; Hu, C. Q. *Phytochemistry* **1999**, *50*, 509.
9. Brader, G.; Bacher, M.; Greger, H.; Hofer, O. *Phytochemistry* **1996**, *42*, 881.
10. Mester, I.; Reisch, J.; Rozsa, Z.; Szendrei, K. *Heterocycles* **1981**, *16*, 77.
11. Khalid, S. A.; Waterman, P. G. *Phytochemistry* **1981**, *20*, 2761.
12. Masuda, T.; Takasugi, M.; Anetai, M. *Phytochemistry* **1998**, *47*, 13.
13. Razdan, T. K.; Qadri, B.; Harkar, S.; Waight, E. S. *Phytochemistry* **1987**, *26*, 2063.
14. Joshi, B. S.; Kamat, V. N.; Gawad, D. H. *J. Chem. Soc., Perkin Trans I* **1974**, 1561.
15. Kozawa, M.; Baba, K.; Minami, M.; Nitta, H.; Heta, K. *Chem. Pharm. Bull.* **1974**, *22*, 2746.
16. Koyama, J.; Toyokuni, I.; Tagahara, J. *Chem. Pharm. Bull.* **1999**, *47*, 1038.
17. Wu, T. S.; Shi, L. S.; Kuo, S. C. *Phytochemistry* **1999**, *50*, 1411.
18. Pouchert, C. J.; Behnke, J. *The Aldrich Library of $^{13}$C and $^1$H FTNMR Spectra*; Aldrich Chemical Company Inc.: USA, 1993, Vol. 2, p 232B.
19. Teng, C. M.; Chen, W. Y.; Ko, W. C.; Ouyang, C. *Biochim. Biophys. Acta* **1987**, *924*, 375.
20. O'Brien, J. R. *J. Clin. Pathol.* **1962**, *15*, 452.
21. Elliott, W. M.; Auersperg, N. *Biotechn. and Histochem*, **1993**, *68*, 29.

C.E.P.S.
