# Supplementary material for: Chalepin: isolated from Ruta angustifolia L. Pers induces mitochondrial mediated apoptosis in lung carcinoma cells
Source: BMC Complement Altern Med. 2016 Oct 12;16:389. doi: 10.1186/s12906-016-1368-6 (PMC5059921; doi:10.1186/s12906-016-1368-6)
Supplement: Additional file 3: — Manuscript of Yang et al., 2013, i.e. reference [10], entitled "Bioactive coumarins from Boenninghausenia sessilicarpa." (PDF 192 kb) [file 12906_2016_1368_MOESM3_ESM.pdf]

Phytochemistry, Vol. 25, No. 9, pp. 2209–2210, 1986.
Printed in Great Britain.

0031-9422/86 \$3.00 + 0.00
Pergamon Journals Ltd.

FOUR AROMATIC DERIVATIVES FROM *RUTA ANGUSTIFOLIA*

J. Borges del Castillo, M. Secundino and F. Rodríguez Luis*

Departamento de Química Orgánica, Facultad de Ciencias, Universidad Autónoma de Madrid, Cantoblanco, 28049-Madrid, Spain;
*Departamento de Química Orgánica, Facultad de Ciencias, Universidad de Cádiz, Apdo 40, Puerto Real, Cádiz, Spain

(*Revised received* 20 *January* 1986)

Key Word Index—*Ruta angustifolia*; Rutaceae; moskachans; moskachans A, B, C and D.

Abstract—A new group of four shikimate metabolites have been isolated from aerial parts of *Ruta angustifolia* and their structures deduced from IR, ^{1}H NMR, ^{13}C NMR and MS data. They have been named moskachans.

INTRODUCTION

In the previous paper [1], we reported on the coumarin and alkaloid content in *Ruta angustifolia* Pers. (Rutaceae). In the present study we describe the isolation and identification of a new group of derivatives of shikimic acid, formula C_6–$C_2(C_2)_n$ where $n = 0$, 2 and 3, and in which the polyketide chain remains linear. These derivatives were named as follows: moskachans A (**1**), B (**2**), C (**4**) and D (**3**), derived from 'moskatxa', the Basque name for this species of *Ruta*.

RESULTS AND DISCUSSION

Four products, probably shikimic acid metabolites, have been isolated from aerial parts of *Ruta angustifolia* Pers. Two of them (**3** and **4**) are new in the literature, while the other two (**1** and **2**), although already described [2–5], have been isolated as secondary metabolites for the first time. These compounds were obtained in small quantities and no attempt for crystallization was made. The structures are determined by their spectroscopic data and their purity determined by GC.

Compound **1** shows IR, ^{1}H NMR and ^{13}C NMR data in agreement with those reported in the literature [4, 5] and was compared with a pure sample synthesized from piperonal and MeMgBr with later oxidation with $K_2Cr_2O_7$–H_2SO_4. Its MS exhibits peaks at *m/z* 149 and 121, for $[M-Me]^+$ and $[M-Ac]^+$, characteristic of those molecules in which the formation of a tropylium ion from benzyl cleavage is prevented. We could explain the ion at *m/z* 135 as a rearrangement of a methyl group and loss of CHO, because the compounds of similar structures exhibit only small peaks for a fragmentation involving loss of formaldehyde from the methylenedioxy group, but larger peaks associated with its retention (at *m/z* 149 and 121) [6]. Its ^{13}C NMR spectrum is in agreement with the literature and the assignment of C-2 and C-5 was based on the observed results in compound **2**.

For compound **2**, the MS data are according with those reported in the literature [2], which results in *m/z* 135 as the base peak, corresponding to the benzylic cleavage in the side chain to give the more stable methylenedioxy ion, which also is present in the remaining compounds. The other peaks can be assigned through rearrangements in the chain. The IR spectrum of **2** showed absorption at 1715 cm^{-1} due to the carbonyl group. It is quite similar to the IR of **4**, but different to that of **1**, where the CO absorption appears at 1665 cm^{-1}.

Compound **3** is the corresponding alcohol of **2** (dihydromoskachan B). Its mass spectrum gave a molecular ion at *m/z* 222, two units more than **2**, and its IR shows an absorption at 3420 cm^{-1}, corresponding to a hydroxyl group. On the other hand, the ^{1}H NMR spectra (Tables 1 and 2) confirm the proposed structure for **3**, which is a methyl alcohol derivative with the same number of carbon atoms as **2**.

Finally, **4** was characterized by the differences in its spectroscopic data in comparison with the data of **2**, since the former has in its ^{1}H NMR (Table 1) a group of methylenes with chemical shifts greater than those observed in **2** (δ 1.21–1.35) and its mass and ^{13}C NMR spectra (Table 2) show two more methylene substituents than **2**.

EXPERIMENTAL

Material was collected during the flowering season, in June, 1982, in the neighbourhood of San Agustín de Guadalix, Madrid,

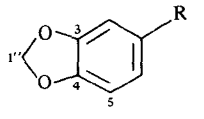

1 R = CO(1′)—Me(2′)

2 R = CH$_2$(1′)—CH$_2$(2′)—CH$_2$(3′)—CH$_2$(4′)—CO(5′)—Me(6′)

3 R = CH$_2$(1′)—CH$_2$(2′)—CH$_2$(3′)—CH$_2$(4′)—CHOH(5′)—Me(6′)

4 R = CH$_2$(1′)—CH$_2$(2′)—CH$_2$(3′)—CH$_2$(4′)—CH$_2$(5)—CH$_2$(6′)—CO(7′)—Me(8′)

Table 1. ^1H NMR data for compounds **1–4** (δ, $CDCl_3$, int. ref. TMS)

**1**	**2**	**3**	**4**
H-2 7.43 *dd*	H-2 6.64 *d*	H-2 6.66 *d*	H-2 6.62 *d*
H-5 6.84 *dd*	H-5 6.70 *d*	H-5 6.72 *d*	H-5 6.68 *d*
H-6 7.55 *dd*	H-6 6.59 *dd*	H-6 6.60 *dd*	H-6 6.57 *dd*
H-1″ 6.04 *s*	H-1″ 5.89 *s*	H-1″ 5.90 *s*	H-1″ 5.88 *s*
H-2′ 2.54 *s*	H-6′ 2.11 *s*	H-6′ 1.17 *s*	H-8′ 2.09 *s*

Coupling constants (Hz). **1**: $J_{2,5} = 0.5$; $J_{2,6} = 1.8$; $J_{5,6} = 8.1$. **2**: $J_{2,6} = 1.7$; $J_{5,6} = 7.8$; δ2.33–2.61, *m*, 2H-1′ + 2H-4′; 1.46–1.68, *m*, 2H-2′ + 2H-3′. **3**: $J_{2,6} = 1.6$; $J_{5,6} = 7.8$; $J_{1',2'} = 6.7$; $J_{5',6'} = 6.2$; δ2.53, *t*, 2H-1′; 3.76, *m*, H-5′; 1.66–1.36, *m*, 2H-2′ + 2H-3′ + 2H-4′. **4**: $J_{2,6} = 1.6$; $J_{5,6} = 7.9$; $J_{1',2'} = 7.3$; δ2.48, *t* (w), 2H-1′; 2.38, *t* (w), 2H-6′; 1.43–1.62, *m*, 2H-2′ + 2H-5′; 1.21–1.35, *m*, 2H-3′ + 2H-4′.

Table 2. ^{13}C NMR data for compounds **1–5** (δ, $CDCl_3$, int. ref. TMS)

C	[1]	**2**	**3**	**4**
1	n.o.	135.9	136.4	136.6
2	108.4	108.7	108.8	108.9
3	n.o.	147.5	147.5	147.4
4	n.o.	145.5	145.4	145.5
5	107.8	108.0	108.0	108.1
6	124.7	121.0	121.0	121.0
1′	n.o.	35.4	35.6	35.4
2′	29.7	31.1	31.4	31.4
3′	—	23.3	23.5	29.1*
4′	—	43.5	39.1	29.0*
5′	—	208.8	68.0	23.9
6′	—	29.8	25.2	43.7
7′	—	—	—	208.8
8′	—	—	—	29.7
1″	100.8	100.7	100.7	100.7

*Signals may be interchanged.
n.o., Not observed under the conditions used.

Spain. Voucher specimens were deposited in the Herbario del Jardín Botánico de Madrid (M.A. 243201).

From the petrol extract of 2.1 kg of dried plant compounds **1** (12 mg), **2** (40 mg) and **4** (20 mg) were isolated; from the $CHCl_3$ extract, 35 mg of **2** and 14 mg of **3** were obtained, in addition to coumarins and alkaloids previously reported [1]. The ^1H NMR spectra were run on a Bruker 2000 (200 Mz) for compounds **2–4**: for **1**, on a Varian XL-100 (100 Mz). The ^{13}C NMR were run on a Bruker 2000 (50.32 Mz). For **3**, the identification of the carbon atoms has been carried out with the DEPT (Distortional Enhancement by Polarization Transfer) technique, which permits the differentiation of the carbon atoms by the number of hydrogens they bear. All the other spectra were uncoupled and compared to those obtained for **3**. The GC, on a Perkin–Elmer 3920 chromatograph (10 ft × 1/8″ column, 10% FFAP on chromosorb W A W DMCS, 235°, with He as carrier gas, at 84 cm^3/min flow.

Moskachan A (**1**). IR ν_{max} cm^{-1}: 2925, 2860, 1665, 1605, 1495, 1460, 1275, 1045, 925, 830: MS *m/z* (rel. int.): 164 (34.4), 149 (82.1), 135 (72.9), 121 (33), 91 (18.8), 77 (19.3), 65 (39.9), 63 (35.5), 57 (10.6), 51 (27.1), 43 (100); UV λ_{max} nm (ε): 229 (14 000), 273 (6700), 308 (7530); GC R_t 336 sec. This compound was identical by spectroscopic data and TLC with a synthetic sample.

Moskachan B (**2**). IR ν_{max} cm^{-1}: 2950, 2860, 1715, 1505, 1495, 1440, 1360, 1245, 1190, 1035, 935; MS *m/z* (rel. int.): 220 (47.1), 162 (11.5), 148 (8.4), 147 (7.5), 135 (100), 91 (5.3), 79 (9.5), 43 (26.8); UV λ_{max} nm (ε): 231 (6000), 287 (5630); GC R_t 852 sec.

Moskachan C (**3**). IR ν_{max} cm^{-1}: 3420, 2960, 2880, 1515, 1500, 1280, 1260, 1245, 1190, 930, 805; MS *m/z* (rel. int.): 222 (22.2), 161 (11.2), 148 (22.1), 136 (23.6), 135 (100), 131 (11.6), 91 (5.7), 77 (15.1), 51 (11.6), 45 (14.5), 43 (8.3); $[\alpha]_D^{25}$ −8.2 (*c* 0.25; CH_2Cl_2); UV λ_{max} nm (ε): 234 (5980), 286 (5740); GC R_t 978 sec.

Moskachan D (**4**). IR ν_{max} cm^{-1}: 2925, 1715, 1680, 1600, 1505, 1490, 1440, 1365, 1280, 1260, 1245, 1190, 930, 805; MS *m/z* (rel. int.): 248 (13.7), 164 (8.3), 149 (20.2), 148 (8.5), 136 (18.6), 135 (100), 121 (7.8), 105 (5.1), 91 (8.3), 77 (13.4), 51 (9.6), 43 (35.3); UV λ_{max} nm (ε): 235 (5540), 288 (5100); GC R_t 1404 sec.

Acknowledgements—We thank Prof. J. Fernández Casas, Departamento de Botánica, Universidad Autónoma de Madrid, for the identification of the plant material, and C.A.I.C.Y.T. (Spain) for financial assistance (ref. 0247–84).

REFERENCES

1. Borges del Castillo, J., Rodríguez Luis, F. and Secundino, M. (1984) *Phytochemistry* **23**, 2095.
2. Semmelhack, M. F. and Bargar, T. (1980) *J. Am. Chem. Soc.* **102**, 7765.
3. Mors, W. D., Gottlieb, O. R. and Djerassi, C. (1957) *J. Am. Chem. Soc.* **79**, 4507.
4. Julia, M. and Binet, C. (1975) *Bull. Soc. Chim. Fr.* 751.
5. Takai, H., So, K. and Sasaki, Y. (1978) *Chem. Pharm. Bull.* **26**, 1303.
6. Willhalm, B., Thomas, A. F. and Gautschi, F. (1964) *Tetrahedron* **20**, 1185.
