# Supplementary material for: Chalepin: isolated from Ruta angustifolia L. Pers induces mitochondrial mediated apoptosis in lung carcinoma cells
Source: BMC Complement Altern Med. 2016 Oct 12;16:389. doi: 10.1186/s12906-016-1368-6 (PMC5059921; doi:10.1186/s12906-016-1368-6)
Supplement: Additional file 4: — Manuscript of Orlita et al., 2008, i.e. reference [11], entitled "Identification of Ruta graveolens L. metabolites accumulated in the presence of elicitors." (PDF 115 kb) [file 12906_2016_1368_MOESM4_ESM.pdf]

This article was downloaded by: [University of Malaya]
On: 03 July 2013, At: 21:54
Publisher: Taylor & Francis
Informa Ltd Registered in England and Wales Registered Number: 1072954 Registered office: Mortimer House, 37-41 Mortimer Street, London W1T 3JH, UK

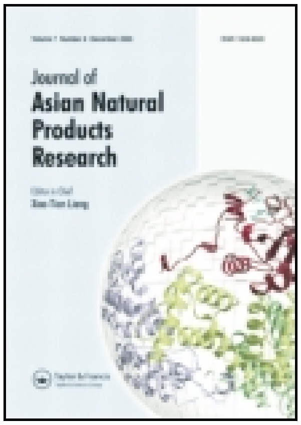

Journal of Asian Natural Products Research
Publication details, including instructions for authors and subscription information:
http://www.tandfonline.com/loi/ganp20

Bioactive coumarins from Boenninghausenia sessilicarpa

Q.-Y. Yang [a], X.-Y. Tian [a] & W.-S. Fang [a]

[a] Key Laboratory of Bioactive Substances and Resources Utilisation of Chinese Herbal Medicine, Ministry of Education, Institute of Materia Medica, Chinese Academy of Medical Sciences, Beijing, 100050, China

Published online: 28 Feb 2007.

To cite this article: Q.-Y. Yang , X.-Y. Tian & W.-S. Fang (2007) Bioactive coumarins from Boenninghausenia sessilicarpa , Journal of Asian Natural Products Research, 9:1, 59-65, DOI: 10.1080/10286020500382397

To link to this article: http://dx.doi.org/10.1080/10286020500382397

PLEASE SCROLL DOWN FOR ARTICLE

Taylor & Francis makes every effort to ensure the accuracy of all the information (the “Content”) contained in the publications on our platform. However, Taylor & Francis, our agents, and our licensors make no representations or warranties whatsoever as to the accuracy, completeness, or suitability for any purpose of the Content. Any opinions and views expressed in this publication are the opinions and views of the authors, and are not the views of or endorsed by Taylor & Francis. The accuracy of the Content should not be relied upon and should be independently verified with primary sources of information. Taylor and Francis shall not be liable for any losses, actions, claims, proceedings, demands, costs, expenses, damages, and other liabilities whatsoever or howsoever caused arising directly or indirectly in connection with, in relation to or arising out of the use of the Content.

This article may be used for research, teaching, and private study purposes. Any substantial or systematic reproduction, redistribution, reselling, loan, sub-licensing, systematic supply, or distribution in any form to anyone is expressly forbidden. Terms & Conditions of access and use can be found at http://www.tandfonline.com/page/terms-and-conditions

Journal of Asian Natural Products Research, Vol. 9, No. 1, January–February 2007, 59–65

Taylor & Francis
Taylor & Francis Group

Downloaded by [University of Malaya] at 21:54 03 July 2013

Bioactive coumarins from *Boenninghausenia sessilicarpa*

Q.-Y. YANG, X.-Y. TIAN and W.-S. FANG*

Key Laboratory of Bioactive Substances and Resources Utilisation of Chinese Herbal Medicine, Ministry of Education, Institute of Materia Medica, Chinese Academy of Medical Sciences, Beijing 100050, China

(Received 16 May 2005; revised 15 August 2005; in final form 9 September 2005)

Bioassay guided fractionation of *Boenninghausenia sessilicarpa* (Rutaceae) resulted in the isolation of a new dimeric coumarin glucoside 9′-methoxyl rutarensin (**1**) and a cytotoxic compound rutamarin (**4**), as well as an antivirus component leptodactylone (**8**), together with six known coumarins. Their structures were elucidated by 1D- and 2D NMR spectroscopy and ESI-MS analyses, respectively. Rutamarin (**4**) showed significant inhibitory activities against A-549, Bel-7402, HepG-2 and HCT-8 tumour cell lines with IC_{50}s of 1.318, 2.082, 2.306 and 2.497 μg/ml. In addition, leptodactylone (**8**) showed potent protective activity on cells infected by SARS-CoV with ratio of 60% at 100 μg/ml.

Keywords: *Boenninghausenia sessilicarpa*; Rutamarin; Leptodactylone; 9′-Methoxyl rutarensin; Anti-tumour activity

1. Introduction

Boenninghausenia sessilicarpa (Rutaceae), a slender and perennial plant, has long been known as a coumarin-rich Chinese herbal medicine distributed in the temperate hilly regions at an altitude of 1500–2500 m in southwestern China, which is traditionally used for the treatment of fever, fester and tonsillitis [1]. The 95% ethanol extract of *B. sessilicarpa* was subjected to MTT cytotoxic assay, exhibiting obvious anti-tumour activity. In order to search the bioactive components of this herb, investigation of *B. sessilicarpa* with a bioassay-guided method led to the isolation of nine coumarins. Their structures were identified as 9′-methoxyl rutarensin (**1**), rutarensin (**2**) [2,3], chalepensin (**3**) [4], rutamarin (**4**) [5], 5,7-dimethoxycoumarin (**5**) [6], xanthotoxin (**6**) [7], isopimpinellin (**7**) [8], leptodactylone (**8**) [9,10] and 5,7,8-trimethoxycoumarin (**9**) [4], on the basis of spectral analyses (figure 1). Among them **4** is the main cytotoxic component of the extract, and 9′-methoxyl rutarensin (**1**) is a new dimeric coumarin glucoside. Moreover, leptodactylone (**8**) was found to have a strong protective effect on virus-infected cells and anti-SARS-CoV activity, and rutamarin (**4**) had a weak protective effect.

*Corresponding author. Email: wfang@imm.ac.cn

Journal of Asian Natural Products Research
ISSN 1028-6020 print/ISSN 1477-2213 online © 2007 Taylor & Francis
http://www.tandf.co.uk/journals
DOI: 10.1080/10286020500382397

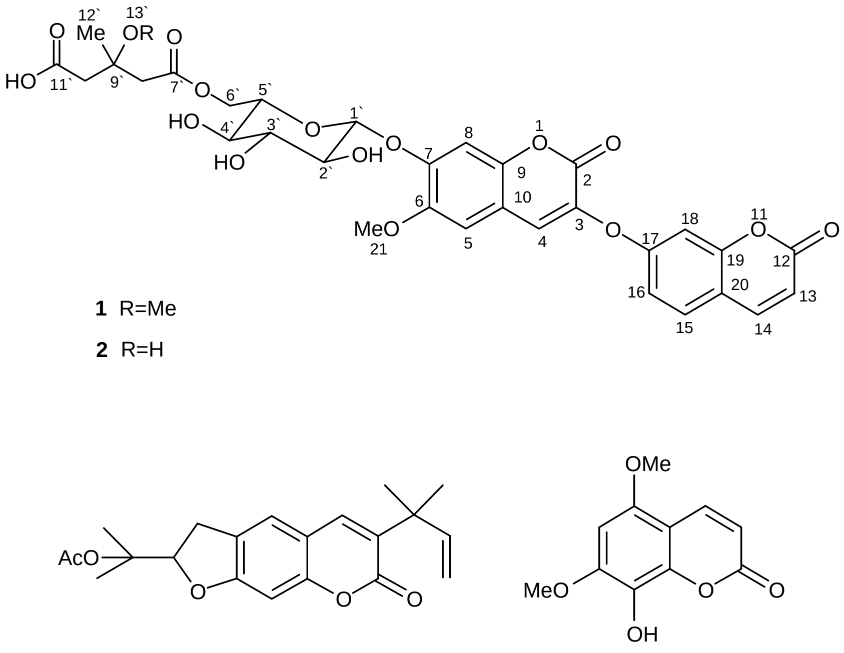

4 **8**

Figure 1. Structures of compounds **1**, **2**, **4** and **8**.

2. Results and discussion

Ethanol extracts of *Boenninghausenia sessilicarpa* were separated into five fractions. As shown in table 1, fraction I exhibited significant anti-tumour activity against Bel-7402 and A-549 tumour cell lines by MTT assay in comparison with other fractions. This fraction was then subjected to silica gel column chromatography to give five fractions. Among them, fraction I-2 showed remarkable cytotoxic activity on the above tumour cell lines. With a bioassay-guided method, fraction I-2 was further purified by repeated chromatography on

Table 1. Cytotoxicities of the fractions from *B. sessilicarpa* on tumour cell lines.

		Cell line	
Sample	*Dose (μg/ml)*	*Bel-7402 (%)*	*A-549 (%)*
Fraction I	50	74.73	69.73
Fraction I-1	5	15	9
Fraction I-2	5	71	66
Fraction I-3	5	25	17
Fraction I-4	5	28	20
Fraction I-5	5	21	18
Fraction II	50	6.13	3.96
Fraction III	50	15.50	20.86
Fraction IV	50	NA	NA
Fraction V	50	NA	NA

NA, not active.

Downloaded by [University of Malaya] at 21:54 03 July 2013

silica gel and reversed-phase preparative HPLC to furnish seven compounds, **3**–**9**. Anti-tumour activity examination of these compounds indicated that **4** showed significant inhibitory effect against several tumour cell lines (table 2), while another six components were found to be almost inactive ($IC_{50} > 5\ \mu g/ml$). The results implicated rutamarin **4** was the major cytotoxic constituent of *B. sessilicarpa*. Moreover, fraction III was found to have moderate cytotoxic activity. Further investigation of this fraction resulted in the isolation of an unknown dimeric coumarin glucoside 9′-methoxyl rutarensin **1**. Its bioactive test is still ongoing. In addition, when the antiviral activities of all coumarin derivatives from *B. sessilicarpa* were evaluated, leptodactylone **8** was found to show potent antiviral activity and rutamarin **4** exhibited weak activity (table 3).

9′-Methoxyl rutarensin **1** was obtained as a yellowish amorphous powder. The ESI-MS of **1** showed a pseudomolecular ion peak $[M + Na]^+$ at *m/z* 695.0, indicating a molecular formula of $C_{32}H_{32}O_{16}$, which was confirmed by the HRFAB-MS. The IR spectrum of **1** indicated the presence of hydroxyl groups ($3410\,cm^{-1}$) and a lactone carbonyl ($1732\,cm^{-1}$). The UV spectrum showed absorption maxima at 236.2 and 308.0 nm, suggesting the presence of coumarin skeleton. The ^1H NMR spectrum for **1** in the downfield region showed one pair of doublets at δ 6.28 and 7.87 ($J = 9.5$ Hz) attributed to the C-13 and C-14 protons of the coumarin nucleus, and an ABX system at δ 7.58 (1H, d, $J = 8.5$ Hz), 7.04 (1H, dd, $J = 8.5$, 2.0 Hz), and 7.02 (1H, d, $J = 2.0$ Hz) confirmed the presence of the benzopyran moiety. Three single aromatic proton signals were shown, one at δ 7.72 assigned to the C-4 proton, the second and third at δ 7.17, 7.25 assigned to the C-5 and C-8 protons, respectively. The doublet at δ 5.03 (1H, $J = 7.0$ Hz) was assigned to the C-1′ proton of the sugar, which should be the β anomer. Remaining protons in the sugar moiety resonated at δ 3.20–4.50 ppm. The signals including an AB quartet at δ 2.59, three singlets at δ 2.71, 3.50, 1.26 in the ^1H NMR spectrum combined with the corresponding carbon signals at δ 45.8, 46.2, 51.9, 28.0, 74.6, 172.4, 173.2 in the ^{13}C NMR spectrum indicated the presence of the 3-methoxy-3-methyl pentadioic acid monoester. The ^{13}C NMR and DEPT spectra of **1** displayed 26 carbon atoms except for the glucose moiety, among which 16 sp^2 carbon atoms, 4 carbonyl carbons, 4 sp^3 carbons and 2 methoxyl carbons were detected. Combining the ^1H NMR and ^{13}C NMR data, a bicoumarin glycoside with 3-methoxy-3-methyl pentatioic acid chain was proposed as the structure for **1**. The imperceptible difference between **1** and **2** on spectral data indicated that the structure of **1** was similar to that of **2** (see table 4), except for a methoxyl group in **1** in place of a hydroxyl group in **2**. In the HMBC experiment (figure 2), the correlations between δ 3.51 ($-OCH_3$) and δ 74.6 (C-9′) enabled us to assign the methoxyl group to C-9′ positions unambiguously. The significant long-range correlations between H-1′

Table 2. Cytotoxicities of **4** against tumour cell lines.

	IC_{50} (μg/ml)			IC_{50} (μg/ml)	
Tumour cell line	*4*	*Taxol*	*Tumour cell line*	*4*	*Taxol*
A-549	1.318	0.0166	KB	>10	0.0046
Bel-7402	2.082	0.0871	BGC-803	>10	<0.001
HepG-2	2.306	0.0047	BGC-823	>10	0.0063
HCT-8	2.497	0.0316	KB/VCR	>10	0.141
A-2780	3.889	0.0054	PC-3M	>10	0.0070
MCF-7	4.500	0.0009	CaEs-17	4.620	<0.001
CNE-2Z	4.543	0.0037			

Downloaded by [University of Malaya] at 21:54 03 July 2013

Downloaded by [University of Malaya] at 21:54 03 July 2013

Table 3. Antiviral activities of **8** and **4**.

Compounds	*Concentration (μg/ml)*	*CPE*[a]	*Effect of cell protection (EC) %*[b]	*Cell toxicity (CC) %*[c]
**8**	100	++	60	41
	20	++++	4	−8
	4	++++	0	−47
**4**	100	++++	10.0	20
	20	+++	27.2	17
	4	++++	10.1	13

[a] CPE (incytopathic effect): " − " means the modality of attached cells were intact, without evident deviation, but the cell amounts were not compared; " + " means the degree of pathological changes of cells: +, <25%; ++, 25–50%; +++, 50–75%; ++++, >75%.
[b] Protective rates against infected-cells: the protective effects of compounds against virus infection were calculated by comparing the OD values of the virus-transfected group, mock-transduced group and compound-treated group. The compounds with protective rate > EC_{20} were considered to have some protective effects on virus-infected cells and have anti-SARS-CoV activity.
[c] Sample toxicity: a compound would not be evaluated with CPE protective rates if its cytotoxicity was more than 50%.

Table 4. ^{13}C NMR and ^{1}H NMR spectral data of **1** and **2**[a].

	1		***2***	
Position	δ_C	δ_H	δ_C	δ_H
2	159.1		157.5	
3	139.1		137.1	
4	130.9	7.72 s	130.6	7.72 s
5	110.3	7.25 s	110.1	7.25 s
6	148.4		146.9	
7	150.6		149.3	
8	105.3	7.17 s	103.6	7.27 s
9	148.0		147.3	
10	114.3		112.9	
12	162.7		160.9	
13	115.1	6.28 d (9.6)	114.6	6.37 d (9.6)
14	145.3	7.88 d (9.6)	144.7	8.03 d (9.6)
15	131.3	7.58 d (8.5)	130.5	7.71 d (8.7)
16	115.0	7.04 dd (9.0, 2.0)	114.3	7.11 dd (8.7, 2.7)
17	161.4		160.0	
18	105.5	7.02 d (2.0)	105.0	7.22 d (2.7)
19	156.7		155.6	
20	116.3		115.2	
21	57.1	3.86 s	56.6	3.84 s
1′	101.7	5.03 d (7.0)	99.3	5.13 d (7.8)
2′	71.7	3.33–3.72 m	73.5	3.15–3.75 m
3′	77.7		76.9	
4′	70.8		70.2	
5′	75.6		74.3	
6′	64.6	H-6a′ 4.45 dd (11.5, 2.0)<br>H-6b′ 4.17 dd (11.5, 6.5)	63.7	H-6a′ 4.27 d (11.7)<br>H-6b′ 4.06 dd (11.7, 6.6)
7′	172.4		171.4	
8′	46.2	2.71 s (2H)	45.7	2.55 d[b] (2H)
9′	74.6		69.5	
10′	45.8	2.59 d (15.5) (2H)	45.5	2.45 d[b] (2H)
11′	173.2		173.1	
12′	28.0	1.26 s	28.1	1.19 s
13′	51.9	3.51 s	–	–

[a] **1** was measured in CD_3OD at 500 MHz for ^{1}H NMR and 125 MHz for ^{13}C NMR. **2** was measured in DMSO at 300 MHz for ^{1}H NMR and 75 MHz for ^{13}C NMR.
[b] Coupling constants were difficult to determine due to interference of the solvent peak.

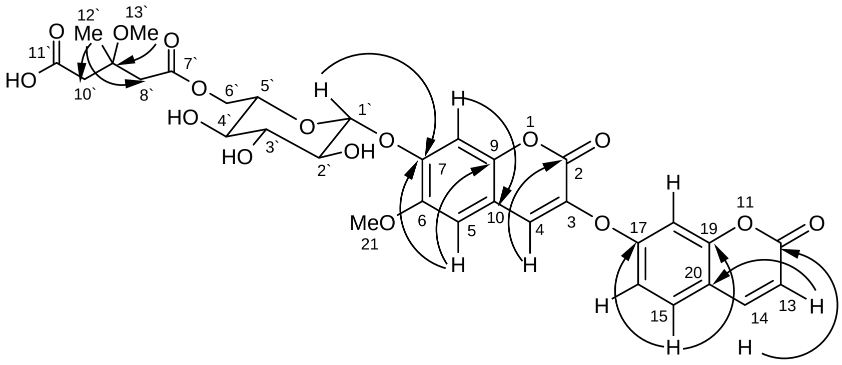

Figure 2. Important HMBC correlations of **1**.

and C-7 confirmed the location of the sugar moiety. From the above evidence, compound **1** was identified as 9′-methoxyl rutarensin.

Chalepensin **3** was previously isolated from *B. albiflora* var. *japonica*, and its partial ^{1}H NMR data have been reported [4]. We now report its complete spectral assignments in this paper.

3. Experimental

3.1 Determination of cell viability by MTT assay

3.1.1 Cell culture. The A-549, Bel-7402, HepG-2, HCT-8 and KB cells were maintained in RPMI-1640 supplemented with 10% foetal bovine serum. The cell cultures were incubated at 37°C in humidified atmosphere with 5% CO_2.

3.1.2 Method. Cells were plated in the appropriate media on 96-well plates in 100 μl total volume at a density of 1×10^4 cells/well. Triplicate wells were treated with media and CAT. The plates were incubated at 37°C in 5% CO_2 for 72 h. Cell viability was determined based on mitochondrial conversion of 3-[4,5-dimethylthiazol-2-yl]-2,5-diphenyltetrazolium bromide (MTT, Sigma) to formazan. The amount of MTT converted to formazan is a sign of the number of viable cells. Each well was supplemented with 50 μl of a 2 mg/ml solution of MTT in complete media. The plates were incubated at 37°C in 5% CO_2 for another 4 h. The medium was carefully removed from each well and 150 μl of DMSO was added. The plates were gently agitated until the colour reaction was uniform and the OD_{570} was determined using a microplate reader. Microsoft Excel software was used for data analysis. Media-only treated cells served as the indicator of 100% cell viability [11].

3.2 Antiviral activity experiments

3.2.1 Sample preparation. The compounds were dissolved in DMSO and diluted to appropriate concentrations (100, 20 and 4 μg/ml) in RPMI-1640.

Downloaded by [University of Malaya] at 21:54 03 July 2013

3.2.2 Method. Vero-E6 cells infected by SARS-CoV were plated in 96-well plates and incubated with different concentrations of compounds in humidified 5% CO_2 atmosphere at 37°C. For each test compound five groups were set up, namely, the virus-transfected group, the mock-transfected group, and three different concentrations of compounds-treated groups. The cell-protective effects (CPE) were detected by fluorescence microscope daily. The OD values were detected by neutral red staining as indicators for the anti-ASRS-CoV effects.

3.3 General experimental procedures

Melting points were determined with a Germany-68992 apparatus and are uncorrected. Optical rotations were measured on a PE-241 digital polarimeter. EI-MS and HRFAB-MS were obtained on a QB-200 mass spectrometer. ESI-MS measurements were carried out at an Agilent 1100 series LC/MSD Trap SL mass spectrometer. IR spectra were recorded on a IR-47 spectrometer. NMR spectra were recorded on a Varian Mercury-400 and Inova-500 spectrometer using TMS as internal standard, and chemical shifts were given on the δ-scale. Assignments were confirmed by COSY, HMBC and HMQC experiments. Silica gel (100–200 mesh) for chromatography and silica gel H for TLC were obtained from Qingdao Marine Chemical Factory, Qingdao, Shandong Province, China. Preparative HPLC was performed on a Shimadzu liquid chromatograph LC-9A instrument with UV–Vis spectrophotometric detector (SPD-6AV) at 254 nm using an ODS column (Unicorn ODS, 5 μm, 300 × 10 mm).

3.4 Plant material

Boenninghausenia sessilicarpa was collected from Dali, Yunnan Province, China, in October 2001, and identified by Professor Guangming Liu at Dali Medicinal College. The authenticated sample of the plant is deposited in the Herbarium of the Institute of Materia Medica, Chinese Academy of Medical Sciences, Beijing, China (No. 0031).

3.5 Extraction and isolation

The air-dried whole plant of *B. Sessilicarpa* (8.4 kg) was powdered and extracted three times with 95% ethanol (3 × 40 L, each 2 h) under reflux. Evaporation of the solvent *in vacuo* provided ethanolic extracts (1.4 kg), which were absorbed to silica gel (60–100 mesh, 2 kg) and eluted with petroleum ether/acetone (95:5), petroleum ether/acetone (50:50), acetone, acetone/methanol (50:50) and methanol to provide fractions I (50 g), II (46 g), III (180 g), IV (90 g) and V (40 g), respectively. Fraction I was subjected to silica gel (100–200 mesh, 3 kg) column chromatography using a petroleum ether/acetone (9:1, 3:2, 1:5) gradient to furnish five fractions [fraction I-1 (5 g), fraction I-2 (8 g), fraction I-3 (10 g), fraction I-4 (12 g), fraction I-5 (12 g)]. Fraction I-2 was further separated by silica gel [100–200 mesh, 900 g, petroleum ether/ethyl acetate (4:1, 1:1, 1:9)] to give four fractions [fraction I-2-1 (1.3 g), fraction I-2-2 (4.3 g), fraction I-2-3 (0.7 g), fraction I-2-4 (1.5 g)]. Compound **3** (800 mg) was obtained from fraction I-2-1 by silica gel (100–200 mesh, 260 g) column chromatography eluting with petroleum ether/ethyl acetate (3:1). Fraction I-2-2 was further purified on silica gel (100–200 mesh, 800 g) column chromatography using petroleum ether/acetone (5:1) as eluent to afford compounds **4** (1.5 g) and **5** (460 mg). Fraction I-2-3 was applied to reversed-phase preparative HPLC [MeOH/H_2O (75:25), flow rate 3.0 ml/min] to afford compounds **6**

Downloaded by [University of Malaya] at 21:54 03 July 2013

(10 mg, t_R 30.17 min) and **7** (5 mg, t_R 41.85 min). Fraction I-2-4 was subjected to silica gel (100–200 mesh, 150 g) column chromatography using 5% MeOH in chloroform as eluent, followed by reversed-phase preparative HPLC [MeOH/H_2O (50:50), flow rate 3.0 ml/min] to give compounds **8** (15 mg, t_R 15.23 min) and **9** (68 mg, 26.46 min). Fraction III (150 g) was firstly divided into three fractions [fraction III-1 (75 g), fraction III-2 (50 g) and fraction III-3 (23 g)] through silica gel (60–100 mesh, 2 kg) column chromatography eluting with a $CHCl_3/CH_3OH/H_2O$ (90:10:1, 80:20:2, 50:50:2) gradient. Fraction III-1 was then subjected to a silica gel column chromatography (100–200 mesh, 1 kg) eluting with 20% MeOH in chloroform, followed by LH-20 column chromatography using $CHCl_3/CH_3OH$ (80:20) as eluent to afford **1** (30 mg) and **2** (6 g).

3.5.1 9′-Methoxyl rutarensin (1). Slightly yellow amorphous powder; mp >275°C; $[\alpha]_D^{25}$ −0.69 (*c* 0.017, DMSO); UV (MeOH): λ_{max} (log ε) = 236 (4.16), 308 (3.98); IR (KBr): ν_{max} = 3410, 1732, 1614, 1506, 1421, 1381, 1276, 1124, 1074 cm^{-1}; HRESI-MS: *m*/*z* = 672.1696 (calcd for $C_{32}H_{32}O_{16}$, 672.1690); ^{1}H NMR and ^{13}C NMR spectral data: see table 4.

3.5.2 Chalepensin (3). Colourless crystals; FAB-MS: *m*/*z* (rel. int.) = 255 ([M + H]$^+$, 100), 254 (20), 239 (10), 231 (14), 199 (21); ^{1}H NMR (300 MHz, CD_3OD) δ (ppm): 1.53 (6H, s, CH_3-14, 15), 5.13 (2H, m, H-13a, 13b), 6.23 (1H, dd, *J* = 10.2, 16.5 Hz, H-12), 6.80 (1H, d, *J* = 2.1 Hz, H-1′), 7.42 (1H, s, H-8), 7.64 (1H, s, H-4), 7.66 (1H, s, H-5), 7.67 (1H, d, *J* = 2.4 Hz, H-2′); ^{13}C NMR (75 MHz, CD_3OD) δ (ppm): 159.8 (C-2), 115.9 (C-3), 119.5 (C-4), 138.3 (C-5), 133.1 (C-6), 151.3 (C-7), 98.9 (C-8), 155.8 (C-9), 124.5 (C-10), 40.5 (C-11), 145.4 (C-12), 112.3 (C-13), 26.1 (14-CH_3), 26.1 (15-CH_3), 106.3 (C-1′), 146.5 (C-2′). Its IR and UV data are the same as in the literature [4].

Acknowledgements

The authors thank the Department of Instrumental Analysis of our institute for the measurement of UV, IR, NMR, and MS spectra. We also acknowledge Beijing Municipal Science and Technology Commission of China for financial support. In addition, we thank Mr Hongyan Liu for the cytotoxicity assays and the Shanghai Institute of Materia Medica for the antiviral assays.

References

[1] S.Q. Luo, Z.K. Lv. *Pharm. Ind.*, **18**, 216 (1987).
[2] H. Fischer, A. Roemer, B. Ulbrich, H. Arens. *Planta Med.*, **54**, 398 (1988).
[3] B. Kreher, A. Neszmelyi, H.D. Wagner. *Phytochemistry*, **29**, 3633 (1990).
[4] J. Reisch, K. Szendrei, E. Minker, I. Novak. *Tetrahedron Lett.*, **41**, 4395 (1968).
[5] X.J. Hao, B.T. Zhao. *Acta Bot. Yunnan*, **16**, 310 (1994).
[6] P. Basnet, S. Kadota, K. Manandhar, M.D. Manandhar, T. Namba. *Planta Med.*, **59**, 384 (1993).
[7] M. Kozawa, K. Baba, M. Minami, H. Nitta, K. Hata. *Chem. Pharm. Bull.*, **22**, 2746 (1974).
[8] K.T. Sunil, K.M. Swapan, T. Bani. *Phytochemistry*, **12**, 2312 (1973).
[9] H. Ishii, J.I. Kobayashi. *Ishikawa. Chem. Pharm. Bull.*, **31**, 3330 (1983).
[10] F.M. Dean, A.M.B. Costa, J.B. Harborne, D.M. Smith. *Phytochemistry*, **17**, 505 (1978).
[11] R. Han. *Chemoprevention and Drug Therapy of Cancer*, p. 29, Peking Union Medical College Press, Beijing (1991).

Downloaded by [University of Malaya] at 21:54 03 July 2013
