# Supplementary material for: Chalepin: isolated from Ruta angustifolia L. Pers induces mitochondrial mediated apoptosis in lung carcinoma cells
Source: BMC Complement Altern Med. 2016 Oct 12;16:389. doi: 10.1186/s12906-016-1368-6 (PMC5059921; doi:10.1186/s12906-016-1368-6)
Supplement: Additional file 5: — Manuscript of Orlita et al., 2008, i.e. reference [12], entitled "Application of chitin and chitosan as elicitors of coumarins and furoquinolone alkaloids in Ruta graveolens L. (common rue)." (PDF 80 kb) [file 12906_2016_1368_MOESM5_ESM.pdf]

Identification of *Ruta graveolens* L. Metabolites Accumulated in the Presence of Abiotic Elicitors

Aleksandra Orlita,† Matylda Sidwa-Gorycka,‡ Jolanta Kumirska,† Edmund Maliński,† Ewa M. Siedlecka,† Jerzy Gajdus,† Ewa Łojkowska,‡ and Piotr Stepnowski*,†

Faculty of Chemistry, University of Gdańsk, PL 80-952 Gdańsk, ul. Sobieskiego 18, Poland, and Intercollegiate Faculty of Biotechnology, Medical University of Gdańsk and University of Gdańsk, ul. Kładki 1, 80-211 Gdańsk, Poland

The study aimed to elucidate the effects of benzothiadiazole (BTH) and saccharin on the biosynthesis of simple coumarins, linear furanocoumarins, dihydrofuranocoumarins, and furoquinolone alkaloids in shoots of *R. graveolens* cultivated in vitro. The biosynthesized metabolites were analyzed and identified by GC−MS and by comparison of Kovats indices. Eight coumarin metabolites were identified: bergapten, chalepin, isopimpinelin, pinnarin, psoralen, rutacultin, rutamarin, and xanthotoxin, and also four alkaloids: dictamnine, γ-fagarine, skimmianine, and kokusaginine. Each of the tested BTH concentrations induced a significant production of furanocoumarins and furoquinolone alkaloids. The use of saccharin also increased the production of bergapten, isopimpinelin, pinnarin, psoralen, and xanthotoxin several times.

Introduction

The in vitro culture of *R. graveolens* is a useful biotechnological source of biologically active linear furanocoumarins and furoquinolone alkaloids. Linear furanocoumarins, particularly xanthotoxin, bergapten, and isopimpinelin, have been applied in the treatment of skin diseases characterized by excessive cell proliferation (e.g., psoriasis, mycosis fungoides) or in pigmentation disorders (e.g., vitiligo) (*1*) and also in neurology (the symptomatic treatment of demyelinating diseases, particularly multiple sclerosis) (*2*). In addition, alkaloids like dictamnine and methoxydictamnine from *R. graveolens* tissues are well-known antimicrobial factors (*3*). Apart from their antibiotic effect, furoquinolone alkaloids appear to have a spasmolytic effect (*4*). The biological effects of furanocoumarins and furoquinolone alkaloids makes them attractive for pharmaceutical uses, hence the considerable interest shown in their availability and sources. However, the commercial production of secondary metabolites is usually limited by their low yield. Elicitation has therefore become an extensively used tool for enhancing secondary metabolites: it is an integral part of any large-scale process for secondary metabolite production.

The mechanisms of action of biotic and abiotic elicitors are thought to be different; they are complex, and many hypotheses have been set up in this regard. Moreover, since little is known about the biosynthetic pathways of secondary metabolites, the effect of an elicitor on a plant cell or tissue culture is not easily predictable; the majority of elicitation approaches are therefore empirical (*5*). The search for elicitors is of the utmost importance in order to obtain the most effective biotechnological system for producing the required metabolites. The objective of our study was to elucidate the effects of saccharin, as well as benzothiadiazole (BTH, benzo(1,2,3)-thiadiazole-7-carbothionic acid *S*-methyl ester) on the biosynthesis of simple coumarins, linear furanocoumarins, dihydrofuranocoumarins, simple coumarins, and furoquinolone alkaloids in shoots of *R. graveolens* cultivated in vitro.

Benzothiadiazole is the main component of the BION preparation (*6*, *7*). Chemically, it is a functional analog of salicylic acid, which is known to be an activator of systemic acquired resistance (SAR) (*7*, *8*). The SAR mechanism plays a central role in induction of disease resistance through the biosynthesis of lipooxygenases or the inhibition of catalase and ascorbic peroxidase, which further limits pathogen infection (*7*, *9*).

So far, little has been discovered about the elicitation potential of saccharin; however, it is known that saccharin can also induce SAR in tissues of the cucumber, pea, and tobacco (*10*). This data motivated us to find out whether saccharin could also act as an elicitor of phytoalexins in our system.

Materials and Methods

In Vitro Culture. The *R. graveolens* shoot cultures were obtained from callus grown. *R. graveolens* shoot cultures were raised from in vitro germinated, surface sterilized seeds obtained from Laboratoire Agronomie et Environment, INPL-INRA, Nancy, France. Seeds were germinated and shoots were grown on B_5 liquid medium supplemented with 3% sucrose (*11*, *12*). Flasks (250 mL) containing 50 mL of medium and the plant biomass were continuously agitated on a rotating (orbital) shaker at 110 rpm, amplitude 9. Cultures elucidated with BTH alone and in the presence of a cell lysates of the *P. atrosepticum* were maintained in duplicate for 28 days at 20 ± 2 °C, under a 16 h photoperiod and illumination of 30−35 mol m^{-2} s^{-1} or in darkness. Cultures elicitated with saccharin were maintained in duplicate only under 16 h photoperiod.

Elicitors. Benzothiadiazole (BTH) is the main component of BION (*6*, *10*); 1%, 3%, and 5% solutions of BTH (Novartis Poland, Warsaw, Poland) were used. Benzothiadiazole elicitors were added to the B_5 medium directly before the cultures were transferred to a fresh flask.

* To whom correspondence should be addressed. Tel. (+48 58) 5235 448. E-mail: sox@chem.univ.gda.pl.
† University of Gdańsk.
‡ Medical University of Gdańsk and University of Gdańsk.

10.1021/bp070261d CCC: $40.75 © 2008 American Chemical Society and American Institute of Chemical Engineers
Published on Web 12/06/2007

Saccharin (Sigma Aldrich) solution was prepared by dissolving 1 g of saccharin powder in 100 mL of deionized, sterile, warm water. The solution was added to the 4-week-old cultures, 6 days before the culture was terminated.

Applied elicitors were added to the cultures media according to the results of the preliminary experiments.

Extraction of Secondary Metabolites. Twenty-eight-day-old co-cultures were harvested and dried for 24 h at 50 °C. Samples (3 g weight of DM) were extracted exhaustively with petroleum ether, chloroform, and methanol in a Soxhlet apparatus. Chloroform and methanol extracts were collected separately, evaporated at 50 °C, and dissolved in ethanol (equivalent of 3 g DM per 5 mL). They were then stored in the dark at room temperature until required for further experiments.

Sample Preparation. Samples were prepared for analysis at room temperature. The filtrate was diluted with 13 mL of a methanol/water (10:3) mixture. The solution was shaken twice with hexane to remove chlorophylls, lipids, and oils. The methanol–water layer was separated, and in the next step, the methanol was removed under a stream of nitrogen. The coumarin-rich fraction was separated from the remaining aqueous solution by addition of 3 mL hydrochloric acid (35–38%) and subsequent extraction twice with chloroform (5 mL). The obtained coumarin fraction was further evaporated under a stream of nitrogen. The residual suspension was then diluted with 1 mL of ethyl acetate, and the coumarins were fractionated on a silica gel column to remove any remaining contamination.

The alkaloid fraction was obtained by addition of 3 g of solid sodium hydroxide to the remaining residual suspension after separation of the coumarins fraction, and re-extraction with chloroform (2 × 5 mL).

Chemicals. Chloroform, petroleum ether, ethyl acetate, methanol, hydrochloric acid (35%), and sodium hydroxide were obtained from POCH (Gliwice, Poland). Deionized water was provided by a MilliQ water purification system (Millipore, Bedford, MA). Stock standard solutions of each coumarin compound were prepared in 10 mL of methanol and then diluted to the required concentration of 1 mg/mL.

The reference standards of the coumarin compounds tested [3-acetylcoumarin, bergapten (5-methoxypsoralen), esculetin (6,7-dihydroxycoumarin), psoralen, scopoletin (7-hydroxy,6-methoxycoumarin), umbelliferone (7-hydroxycoumarin), xanthotoxin (8-methoxypsoralen), 7-methoxycoumarin, 4-methylcoumarin, 7,8-dihydroxy,6-methoxy-coumarin, and coumarin] were purchased from Sigma Aldrich.

GC and GC–MS. A 8000 TOP CE Instruments gas chromatograph was used together with a fused-silica capillary column containing EC-1 (30 m × 0.25 i.d., 0.25 μm film thickness) equipped with a flame ionization detector. The carrier gas was argon. In each case 1 μL of sample was injected in splitless mode. The operating conditions were as follows: initial GC column temperature 100 °C; after injection, temperature held at 100 °C for 5 min; temperature programming at 4 °C min^{-1} to 320 °C. The injector and detector temperatures were both 320 °C.

Individual compounds were identified from their electron impact mass spectral patterns and compared with the literature data. EI-MS analyses were done on a Trio-3 mass spectrometer (VG Masslab Ltd). The samples were introduced through a Hewlett-Packard 5890 gas chromatograph equipped with a BP-1 capillary column (30 m × 0.25 i.d., 0.25 μm film thickness) (Alltech, Poland) in the following temperature program: 4 °C min^{-1} from 100 to 320 °C and held for 5 min.

Table 1. Kovats Indices Calculated for Six Coumarins Found in Extracts of *Ruta graveolens* L. Shoot Cultures

compounds	Kovats indices
coumarin[b]	1429
3-acetylcoumarin[b]	1718
7-methoxycoumarin[b]	1744
6,7-dimetoxycoumarin [c]	2028
psoralen[b]	1842
6,7-dimetoxy, 4-metylocoumarin [c]	2166
bergapten[c]	2120
xanthotoxin[c]	2100
scopolein[c]	2028
7,8-dihydroxy, 6-metoxycoumarin[c]	2187
7-hydroxycoumarin[b]	1836

[a] Values for coumarin standards were the same. Indices derived from the net retention data on a semipolar phase (DB-5). [b] 80–200 °C, at 0.9 °C/min, injector and detector temperature 220 °C, carrier gas at 86 kPa. [c] 160–260 °C, at 3 °C /min, injector and detector temperature 280 °C, carrier gas at 86 kPa.

Kovats Retention Indices. The mixture of *n*-alkanes was prepared as follows: Each hydrocarbon (from C_{12} to C_{19}) was diluted in toluene to a concentration of 1 mg/mL; then 100 μL portions of each individual alkane solution were combined. The same was done to produce the C_{19}–C_{24} mixture.

The mixture of standard coumarins was prepared as follows: Each standard compound was diluted in toluene to a concentration of 1 mg/mL. Then, 100 μL portions of each solution of coumarin, 7-hydroxycoumarin, 3-acetylcoumarin, psoralen, and 7-methoxycoumarin were combined. A further 50 μL of the C_{12}–C_{19} *n*-alkane mixture was added to the solution of coumarin standards.

The same was done to produce a solution of the standards of the following compounds: 6,7-dimethylcoumarin, 6,7-dimethoxy-4-methylcoumairn, bergapten, xanthotoxin, and scopolamine. The solution was further spiked with 100 μL of the C_{19}–C_{24} *n*-alkane mixture.

In the case of the coumarins from the natural extract, 200 μL of the *R. graveolens* extract was split into two portions. One (100 μL) was spiked with 40 μL of the C_{12}–C_{19} *n*-alkane mixture, and the other with the same amount of the C_{19}–C_{24} *n*-alkane mixture.

The solutions prepared in this way were further analyzed in order to calculate the Kovats indices of the analyzed coumarins.

The components were separated by flow splitting (1:12) on a GC 8000 TOP CE Instruments gas chromatograph equipped with a flame ionization detection (GC-FID) system on a fused-silica capillary column DB-5 (Restek, USA) (30 m × 0.25 i.d., 0.25 μm film thickness). Two temperature programs were applied: (1) column temperature programmed at 0.9 °C/min from 80 to 200 °C, injector and detector temperatures both 220 °C; (2) column temperature programmed at 3 °C/min from 160 to 260 °C, injector and detector temperatures both 280 °C. The net-retention times were further used to calculate the linear Kovats indices *I* values according to the following equation:

$$I = 100z + 100\frac{t_R - t_{Rz}}{t_{R(z+1)} - t_{Rz}}$$

where *z* is the number of carbon atoms in the alkane eluting before the analyzed compound, and t_{Rz} and $t_{R(z+1)}$ are the net retention times of *n*-alkanes eluting before (*z* carbon atoms) and after ($z + 1$ carbon atoms) the analyzed compound, respectively.

Results and Discussion

Identification of Coumarins and Furoquinolone Alkaloids. Since it is impossible to define the type of isomers of the

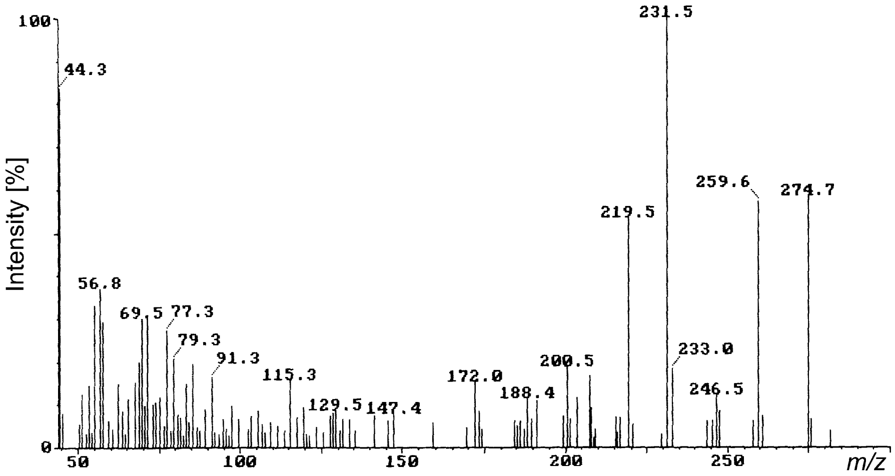

Figure 1. Mass spectrum (EI, 70 eV) of pinnarin: *m/z* 274 $[M]^+$ (74%), 259 $[M - Me]^+$ (100%), 231 $[M - CH_3CO]^+$ (58%), and 219 $[M - 55u]^+$ (95%).

secondary metabolites analyzed here solely on the basis of mass spectra, the coumarins and furoquinolone alkaloids were additionally identified on the basis of calculated Kovats indices. Because of differences in the volatility of the compounds, the indices were derived from retention data obtained in two temperature programs. Table 1 presents the mean indices for six coumarins found in the extract of *R. graveolens* shoot cultures (values for standard coumarins calculated from five chromatographic runs were the same).

The following important ion fragments were registered in the EI-MS mode.

Two simple coumarins:

• rutacultin at *m/z* 274 $[M]^+$ (74%), 259 $[M - Me]^+$ (100%), 231 $[M - CH_3CO]^+$ (58%) ,and 219 $[M - 55u]^+$ (95%);

• pinnarin (Figure 1) at *m/z* 274 $[M]^+$ (74%), 259 $[M - Me]^+$ (100%), 231 $[M - CH_3CO]^+$ (58%), and 219 $[M - 55u]^+$ (95%).

Two dihydrofuranocoumarins:

• rutamarin (Figure 2) at *m/z* 356 $[M]^+$ (11%), 281 $[M - aryl]^+$ (100%), 341 $[M - Me]^+$ (7%), 296 (23%) formed as a result of alkyl chain fragmentation, 313 (9%) $[M - 43u]^+$ characteristic of a coumarin fragment substituted in the lactone ring, 301 (very low intensity) formed by the elimination of the 55u fragment, 253 (16%) formed as a result of the elimination of CO from the *m/z* 281 ion;

• chalepin at *m/z* 314 $[M]^+$(94%), 299 $[M - Me]^+$ (100%), 255 $[M - OHC^\bullet (CH_3)_2]^+$ (53%), 281 (36%) formed as a result of side chain fragmentation.

Four furanocoumarins:

• psoralen at *m/z* 186 $[M]^+$ (100%), 158 $[M - CO]^+$ (65%), 130 $[M - CO - CO]^+$ (19%) and 102 $[M - CO - CO - CO]^+$ (32%);

• xanthotoxin (Figure 3) at *m/z* 216 $[M]^+$ (100%), 201 $[M - Me]^+$ (29%), 188 $[M - CO]^+$ (14%), 173 $[M - Me - CO]^+$ (54%), 145 $[M - Me - CO - CO]^+$ (21%), 89 (26%) and 63 (15%);

• bergapten at *m/z* 216 $[M]^+$ (100%), 201 $[M - Me]^+$ (29%), 188 $[M - CO]^+$ (14%), 173 $[M - Me - CO]^+$ (54%), 145 $[M - Me - CO - CO]^+$ (21%) and 89 (26%);

• isopimpinelin at *m/z* 246 $[M]^+$ (79%), 231$[M - Me]^+$ (100%), 203 $[M - Me - CO]^+$ (19%), 188 (28%), 175 (26%), 147 (11%) and 89 (25%).

Four alkaloids:

• dictamnine at *m/z* 199 $[M]^+$ (100%), 184 $[M - Me]^+$ (41%), 156 (32%), 128 (19%);

• γ-fagarine at *m/z* 229 $[M]^+$ (100%), 214 $[M - Me]^+$ (36%), 200 $[M - CHO^\bullet]^+$ (91%), 156 (25%), 128 (10%);

• skimmianine at *m/z* 259 $[M]^+$ (51%), 244 $[M - Me^+]$ (100%), 230 (68%);

• kokusaginine (Figure 4) at *m/z*: 259 $[M]^+$ (100%), 244 $[M - Me]^+$ (11%), 230 $[M - CHO^\bullet]^+$ (63%), 216 (27%), 201 (23%), 173 (18%).

The EI−MS spectra of the coumarins is very characteristic of this type of compound, and the fragments presented here are consistent with those found in the literature (*13*−*17*). The molecular ions $[M^{+\bullet}]$ obtained are always present, usually at high intensities. One of the characteristic fragmentation steps of all these compounds is the elimination of the CO moiety from the pyrone ring. The residual oxygen atoms are also eliminated through the loss of CO or as the $CHO^\bullet$ radical (*15*, *18*). In the spectra of simple coumarins the presence of $[M - 15]^+$ indicates a methoxy functional group at position 6 in the benzo-α-pyrone ring (*18*, *19*).

The 4-methoxycoumarins fragment differently in comparison with the other coumarins.

The molecular ions of these coumarins were found to fragment via the displacement of a hydrogen atom, following a retro-Diels−Alder (RDA) recombination. In most cases, this leads to the formation of an ion at *m/z* 93. The enolic nature of these ions was also confirmed. The mass spectra of long chain coumarins are much more difficult to interpret; the fragmentations depend on the substituted functional groups and the substitution site in the molecule (*20*, *21*).

The mass spectra of furanocoumarins are similar to those characterizing simple coumarins. The furan ring does not affect the fragmentation process. Therefore, the spectrum of psoralen is characteristic of unsubstituted furanocoumarins (*18*).

However, in methoxylated furanocoumarins a very intensive $[M - 15]^+$ is present, formed when a methyl radical is eliminated. Additionally, characteristic fragmentation ions are formed as a result of the loss of CO. Therefore, in the spectrum of bergapten, there is an ion at *m/z* 201 ($[M - 15]^+$) as well as one at *m/z* 188 ($[M - CO]^{+\bullet}$). The ion at *m/z* 201 ($[M - CO]^{+\bullet}$) fragments further to yield another characteristic ion at *m/z* 201 (*13*, *15*, *22*).

Effect of BTH. Table 2 shows the results of BTH elicitation of the secondary metabolites in the *R. graveolens* shoots tissue. It was found that each of the tested BTH concentrations induced a significant production of coumarins as compared to the control

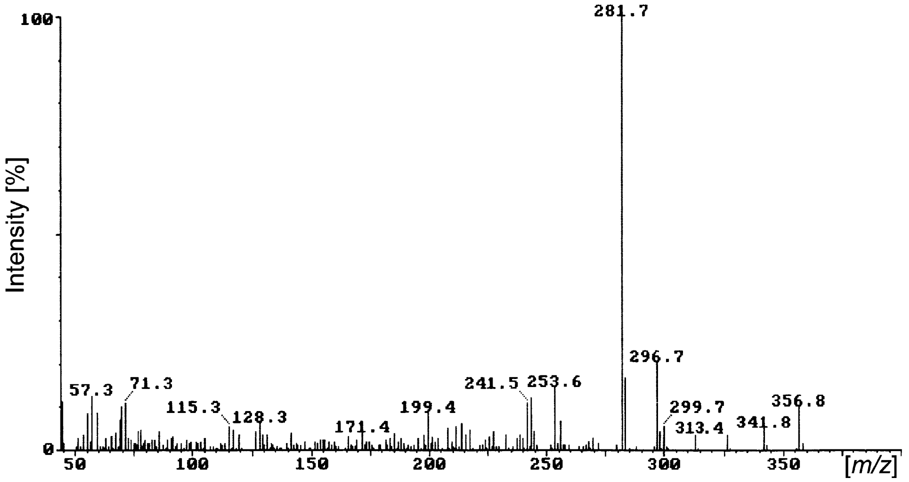

Figure 2. Mass spectrum (EI, 70 eV) of rutamarin: *m/z* 356 $[M]^+$ (11%), 341 $[M - Me]^+$, (7%), 313 (9%) $[M - 43u]^+$, 281 $[M - aryl]^+$ (100%), and 296 (23%).

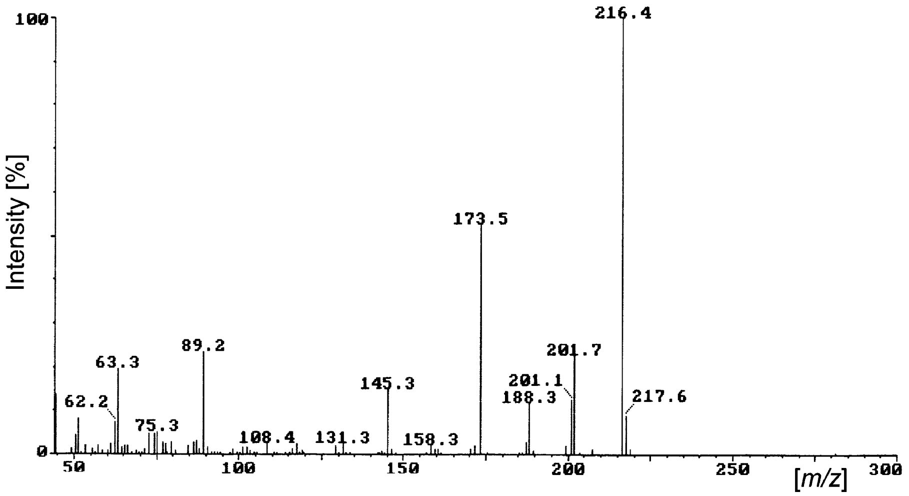

Figure 3. Mass spectrum (EI, 70 eV) of xanthotoxin: *m/z* 216 $[M]^+$ (100%), 201 $[M - Me]^+$ (29%), 188 $[M - CO]^+$ (14%), 173 $[M - Me - CO]^+$ (54%), 145 $[M - Me - CO - CO]^+$ (21%), 89 (26%), and 63 (15%).

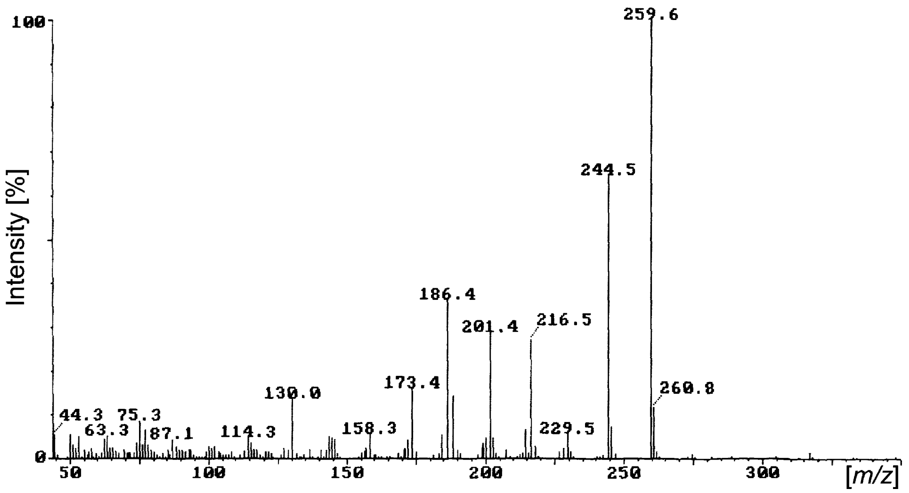

Figure 4. Mass spectrum (EI, 70 eV) of kukusaginine: *m/z* 259 $[M]^+$ (100%), 244 $[M - Me]^+$ (11%), 230 $[M - CHO^{\bullet}]^+$ (63%), 216 (27%), 201 (23%), and 173 (18%).

sample. Therefore, BTH appeared to be an effective elicitor of coumarins and furanocoumarins accumulation.

The addition of BTH at the highest applied concentration (5%) led to the highest rate of coumarin synthesis: the rate of synthesis of isopimpinelin was elevated 14 times (789.0 μg/g dry wt), that of pinnarin 12 times (357.2 μg/g dry wt), that of bergapten 3.7 times (1537.8 μg/g dry wt), and those of rutacultin and rutamarin c. 2.5 times. When 1% BTH was used as elicitator but plants were grown in the dark, the coumarin concentrations either dropped (xanthotoxin, psoralen, and rutacultin), with values close to the detection limit, or rose only slightly (pinnarin and isopimpinelin).

A similar effect was observed for the furoquinolone alkaloids. Each of the BTH concentrations tested significantly induced

Table 2. BTH Elicitation of Secondary Metabolites in *Ruta graveolens* L. Shoots[a]

	concn of secondary metabolites (μg/g dry wt)				
compound	control	1%BTH	3% BTH	5% BTH	1% BTH (darkness)
		Simple Coumarins			
pinnarin	28.0	46.3	103.0	357.2	38.1
rutacultin	136.0	76.5	113.7	323.3	n.a.
		Furanocoumarins			
bergapten	412.0	612.4	819.7	1537.8	39.2
isopimpinelin	56.0	97.4	325.0	789.0	67.9
psoralen	n.a.	634.7	206.0	820.0	n.a.
xanthotoxin	338.0	1223.0	1624.2	2883.6	n.a.
		Dihydrofuranocoumarins			
rutamarin	224.0	147.1	225.0	565.0	8.7
		Furoquinolone Alkaloids			
dyctamnine	n.a.	n.a.	n.a.	n.a.	n.a.
γ-fagarine	4.9	27.9	44.2	58.5	n.a.
kokusaginine	5.3	12.2	20.7	28.2	3.9
skimmianine	4.1	43.8	58.3	64.5	2.3

[a] Given values represent metabolites concentration in μg/g calculated to the dry weight. n.a. = not analyzed (below detection limit).

Table 3. Saccharin Elicitation of Secondary Metabolites in *Ruta graveolens* L. Shoots

	concn of secondary metabolites (μg/g dry wt)	
compound	control	saccharin 0.0003%
	Simple Coumarins	
pinnarin	28.0	316.0
rutacultin	136.0	216.4
	Furanocoumarins	
bergapten	412.0	1186.3
isopimpinelin	56.0	846.9
psoralen	n.a.	380.0
xanthotoxin	338.0	1887.2
	Dihydrofuranocoumarins	
rutamarin	224.0	387.0

[a] Given values represent metabolites concentration in μg/g calculated to the dry weight. n.a. = not analyzed (below detection limit).

the production of these compounds in comparison to the control sample. The addition of BTH at the highest applied concentration (5%) led to the highest rate of alkaloid synthesis: the rate of synthesis of skimmianine was elevated 15.7 times (64.5 μg/g dry wt), γ-fagarine 12 times (58.5 μg/g dry wt) and kokusaginine 5.3 times (28.2 μg/g dry wt). When the elicitation experiments were run in the dark (in the presence of 1% BTH), alkaloid concentrations was not higher than in control.

During earlier study it was shown that BTH induce accumulation of secondary metabolites in *Ammi visnaga* L. callus and cell suspensions. The stimulation of accumulation of visnagin, umbelliferone, scopoletin, and dehydrogeyerin in shoot tissues was also observed (*23*). There is limited information about coumarins elicitation in *R. graveolens* tissue by abiotic compounds. In the case of common application of BTH and biotic elicitor, in cell lysates of the *P. atrosepticum*, the induction of secondary metabolites were much less effective than after application of BHT only.

Effect of Saccharin. Table 3 lists the results of saccharin elicitation of secondary metabolites of *R. graveolens* L. shoots. It was found that in the presence of 0.0003% of saccharin, coumarin biosynthesis was significantly induced in comparison to the control sample. This abiotic activator can therefore be regarded as a potentially effective elicitor of phytoalexins in *R. graveolens*. The application of saccharin raised the production of isopimpinelin (846.9 μg/g dry wt) 15 times, that of pinnarin (316.0 μg/g dry wt) 11 times, and those of xanthotoxin and bergapten 5 and 3 times, respectively. Under the same experimental conditions, concentrations of rutacultin and rutamarin were elevated >1.5 times. In the case of psoralen, it was impossible to detect any amount of this coumarin in non-induced shoots, but in the presence of saccharin 380 μg/g dry wt was accumulated by *R. graveolens*.

Conclusions

An in vitro culture of *R. graveolens* L. is a useful biotechnological source of biologically active linear furanocoumarins and furoquinolone alkaloids. This study aimed to elucidate the effects of saccharin and BTH on the biosynthesis of simple coumarins, linear furanocoumarins, dihydrofuranocoumarins, and furoquinolone alkaloids in shoots of *R. graveolens* cultivated in vitro. It was found that each of the tested BTH concentrations induced a significant production of coumarins in comparison to the control sample. It appears, therefore, that BTH is not only an activator of systemic acquired resistance but also an effective elicitor of phytoalexins. It was also found that the application of saccharin led to a several-times higher production of bergapten, isopimpinelin, pinnarin, psoralen, and xanthotoxin.

Further studies should be undertaken to obtain data on the effectiveness of elicitation over time. In addition, a search for other biosources of coumarins and alkaloids in the presence of BTH and/or saccharin should be undertaken. These preliminary results have demonstrated the very considerable utility of both elicitors in the production of coumarins and alkaloids by *R. graveolens*, which is a promising prospect for the biotechnological production of these valuable metabolites.

Acknowledgment

Financial support was provided by the Polish Ministry of Research and Higher Education under grant DS 8200-4-0085-7.

References and Notes

(1) Ekiert, H.; Czygan, F. C. Accumulation of biologically active furanocoumarins in agitated cultures of *Ruta graveolens* L. and *Ruta graveolens* ssp. *divaricata* (Tenore) *Gams. Pharmazie* **2005**, *60*, 623−626.

(2) Bohuslavizki, K. H.; Hansel, W.; Kneip, A.; Koppenhofer, E.; Niemoller, E.; Sanmann, K. Mode of action of psoralens, benzofurans, acridinons, and coumarins on the ionic currents in intact myelinated nerve fibres and its significance in demyelinating diseases. *Gen. Physiol. Biophys.* **1994**, *13*, 309−328.

(3) Wolters, B.; Eilert, U. Antimicrobial substances in callus cultures of *Ruta graveolens*. *Planta Med.* **1981**, *43*, 166−174.

(4) Minker, E.; Bartha, C.; Rozsa, Z.; Szendrei, K.; Reisch, J. Antispasmogenic effect of rutamarin and arborinine on isolated smooth muscle organs. *Planta Med.* **1979**, *37*, 156−160.

(5) Bhagwath, S. G.; Hjortso, M. A. Statistical analysis of elicitation strategies for thiarubrine A production in hairy root cultures of *Ambrosia artemisiifolia*. *J. Biotechnol.* **2000**, *80*, 159−167.

(6) Schweizer, P.; Schlagenhauf, E.; Schaffrath, U.; Dudler, R. Different patterns of host genes one induced in rice by Pseudomonas syringae a biological inducer of resistance and the chemical inducer benzothiadiazole (BTH). *Eur. J. Plant Biol.* **1999**, *105*, 659−665.

(7) Koo, Y. J.; Kim, M. A.; Kim, E. H.; Song, J. T.; Jung, C.; Moon, J. K.; Kim, J. H.; Seo, H. S.; Song, S. I.; Kim, J. K.; Lee, J. S.; Cheong, J. J.; Choi, Y. D. Overexpression of salicylic acid carboxyl methyltransferase reduces salicylic acid-mediated pathogen resistance in *Arabidopsis thaliana*. *Plant Mol. Biol.* **2007**, *64*, 1−15.

(8) Kim, M. R.; El-Aty, A. M.; Kim, I. S.; Shim, J. H. Determination of volatile flavor components in danggui cultivars by solvent free injection and hydrodistillation followed by gas chromatographic-mass spectrometric analysis. *J. Chromatogr. A* **2006**, *1116*, 259−264.

(9) Katz, V. A.; Thulke, O. U.; Conrath, U. A. Benzothiadiazole primes parsley cells for augmented elicitation of defense responses. *Plant Physiol.* **1998**, *117*, 1333−1339.

(10) Liu, H.; Jiang, W.; Bi, Y.; Luo, Y. Postharvest BTH treatment induces resistance of peach (*Prunus persica* L. cv. Jiubao) fruit to infection by *Penicillium expansum* and enhances activity of fruit defense mechanisms. *Postharv. Biol. Biotechnol.* **2005**, *35*, 263−269.

(11) Massot, B.; Milesi, S.; Gontier, E.; Bourgaud, F.; Guckert, A. Optimized culture conditions for the production of furanocoumarins by micropropagated shoots of *Ruta graveolens*. *Plant Cell, Tissue Organ Cult.* **2000**, *62*, 11−19.

(12) Sidwa−Gorycka, M.; Krolicka, A.; Kozyra, M.; Głowniak, K.; Bourgaud, F.; Lojkowska, E. Establishment of a coculture of *Ammi majus* L. and *Ruta graveolens* L. for the synthesis of furanocoumarins. *Plant Sci.* **2003**, *165*, 1315−1319.

(13) Dercks, W.; Trumble, J.; Winter, C. Impact of atmospheric pollution on linear furanocoumarin content in celery. *J. Chem. Ecol.* **1990**, *16*, 443−453.

(14) Dugo, P.; Mondello, L.; Lamonica, G.; Dugo, G. Characterization of cold-pressed key and persian lime oils by gas chromatography, gas chromatography/mass spectroscopy, high-performence liquid chromatography and physicochemical indices. *J. Agric. Food Chem.* **1997**, *45*, 3608−3616.

(15) Hamerski, D.; Beier, R. C.; Kneusel, R. E.; Matern, U.; Himmelspach, K. Accumulation of coumarins in elicitor-treated cell suspension cultures of *Ammi majus*. *Phytochemistry* **1990**, *29*, 1137−1142.

(16) Ivie, G. W. Linear furocoumarins (psoralens) from the Seed of Texas *Ammi majus* l. (Bishop's weed). *J. Agric. Food Chem.* **1978**, *26*, 1394−1403.

(17) Weinberg, D. S.; Manier, M. L.; Richardson, M. D.; Haibach, F. G. Identification and quantification of coumarin, phthalide, and sesquiterpene compliance markers in an *Umbelliferous* vegetable beverage. *J. Agric. Food Chem.* **1993**, *41*, 48−51.

(18) Mabry, T. J.; Ulubelen, A. Flavonoids and Related Plant Phenolics. In *Biochemical Applications of Mass Spectrometry*; Waller, G. R., Dermer, O. C., Eds.; John Wiley & Sons, Inc.: New York, 1980.

(19) Kuo, P. C.; Hsu, M. Y.; Damu, A. G.; Su, C. R.; Li, C. Y.; Sun, H. D.; Wu, T. S. Flavonoids and coumarins from leaves of *Phellodendron chinense*. *Planta Med.* **2004**, *70*, 183−185.

(20) Kotha, S.; Banerjee, S.; Patil, M. P.; Sunoj, R. B. Retro Diels-Alder reaction under mild conditions: experimental and theoretical studies. *Org. Biomol. Chem.* **2006**, *4*, 1854−1856.

(21) Wijnen, J. W.; Engberts, J. B. Retro-Diels-Alder Reaction in Aqueous Solution: Toward a better understanding of organic reactivity in water. *J. Org. Chem.* **1997**, *62*, 2039−2044.

(22) Cho, Y. H.; Kim, J. H.; Park, S. M.; Lee, B. C.; Pyo, H. B.; Park, H. D. New cosmetic agents for skin whitening from *Angelica dahurica*. *J. Cosmet. Sci.* **2006**, *57*, 11−21.

(23) Staniszewska, I.; Królicka, A.; Maliński, E.; Szafranek, J. Elicitation of secondary metabolites in in vitro cultures of *Ammi majus* L. *Enz. Microb. Technol.* **2003**, *33*, 565−568.

Received August 1, 2007. Accepted Octobert 30, 2007.

BP070261D
