# Supplementary material for: Chalepin: isolated from Ruta angustifolia L. Pers induces mitochondrial mediated apoptosis in lung carcinoma cells
Source: BMC Complement Altern Med. 2016 Oct 12;16:389. doi: 10.1186/s12906-016-1368-6 (PMC5059921; doi:10.1186/s12906-016-1368-6)
Supplement: Additional file 6: — GCMS analysis of isolated compounds which includes the total ion chromatogram (TIC) and mass spectrum data. (PDF 12380 kb) [file 12906_2016_1368_MOESM6_ESM.pdf]

Biotechnology and Applied Biochemistry

www.babonline.org

Biotechnol. Appl. Biochem. (2008) 51, 91–96 (Printed in Great Britain) doi:10.1042/BA20070200

Application of chitin and chitosan as elicitors of coumarins and furoquinolone alkaloids in *Ruta graveolens* L. (common rue)

Aleksandra Orlita*, Matylda Sidwa-Gorycka†, Monika Paszkiewicz*, Edmund Malinski*, Jolanta Kumirska*, Ewa M. Siedlecka*, Ewa Łojkowska† and Piotr Stepnowski*[1]

**Faculty of Chemistry, University of Gdańsk, ul. Sobieskiego 18, PL 80-952 Gdańsk, Poland, and †Intercollegiate Faculty of Biotechnology, University of Gdańsk and Medical University of Gdańsk, ul. Kładki 1, 80-211 Gdańsk, Poland*

Common rue (*Ruta graveolens* L.) accumulates various types of secondary metabolites, such as coumarins furanocoumarins, acridone and quinolone alkaloids and flavonoids. Elicitation is a tool extensively used for enhancing secondary-metabolite yields. Chitin and chitosan are examples of elicitors inducing phytoalexin accumulation in plant tissue. The present paper describes the application of chitin and chitosan as potential elicitors of secondary-metabolite accumulation in *R. graveolens* shoots cultivated *in vitro*. The simple coumarins, linear furanocoumarins, dihydrofuranocoumarins and furoquinolone alkaloids biosynthesized in the presence of chitin and chitosan were isolated, separated and identified. There was a significant increase in the growth rate of *R. graveolens* shoots in the presence of either chitin or chitosan. Moreover, the results of the elicitation of coumarins and alkaloids accumulated by *R. graveolens* shoots in the presence of chitin and chitosan show that both compounds induced a significant increase in the concentrations of nearly all the metabolites. Adding 0.01 % chitin caused the increase in the quantity (μg/g dry weight) of coumarins (pinnarin up to 116.7, rutacultin up to 287.0, bergapten up to 904.3, isopimpinelin up to 490.0, psoralen up to 522.2, xanhotoxin up to 1531.5 and rutamarin up to 133.7). The higher concentration of chitosan (0.1 %) induced production of simple coumarins (pinnarin up to 116.7 and rutacultin up to 287.0), furanocoumarins (bergapten up to 904.3, isopimpinelin up to 490.0, psoralen up to 522.2, xanhotoxin up to 1531.5) and dihydrofuranocoumarins (chalepin up to 18 and rutamarin up to 133.7). Such a dramatic increase in the production of nearly all metabolites suggests that these compounds may be participating in the natural resistance mechanisms of *R. graveolens*. The application of chitin- and chitosan-containing media may be considered a promising prospect in the biotechnological production of xanthotoxin, isopimpinelin, psoralen, chalepin or methoxylated dictamnine derivatives.

Introduction

Ruta graveolens L. (common rue) accumulates various types of secondary metabolites, such as coumarins, furanocoumarins, acridone and quinolone alkaloids, and flavonoids. Coumarins and furanocoumarins have been successfully and effectively used in the symptomatic treatment of demyelinating diseases, particularly multiple sclerosis [1], and of leucoderma/vitiligo (acquired depigmentation of the skin) and psoriasis [2]. Many alkaloids, including those previously found in *Ruta* tissues (dictamnine and methoxydictamnine), have been found to possess antimicrobial properties [3]. The properties of the secondary metabolites of *R. graveolens* have attracted commercial interest, especially in their natural form. *In vitro* cultures of *R. graveolens* are a good biotechnological source of biologically active phytoalexins [4–6].

A major reason why the commercial production of secondary metabolites using plant culture has not met with success is their low yield. Elicitation is a tool extensively used for enhancing secondary-metabolite yields, and elicitors are compounds that induce plants to synthesize elevated levels of phytoalexins. There are reports, however, that the synthesis of secondary metabolites other than phytoalexins can also be stimulated by elicitors [7–9]. In addition to reducing the time needed to reach a high product concentration, elicitation as a process strategy is readily integrated with other yield-enhancement methodologies such as *in situ* product removal [10]. The modes of action of elicitors are complex and there are many hypotheses regarding the mechanism of elicitation [7]. Moreover, since little is known about the biosynthetic pathways of most secondary metabolites, the effect of an elicitor on a plant cell or tissue culture cannot easily be predicted. The majority of elicitation approaches are therefore empirical [11].

Key words: alkaloids, coumarins, elicitation, *in vitro* cultures, *Ruta graveolens* L. (common rue), secondary metabolites.

Abbreviation used: EI, electron impact.

[1] To whom correspondence should be addressed (email sox@chem.univ.gda.pl).

© 2008 Portland Press Ltd

A wide variety of elicitors have been employed to alter cell metabolism in order to induce plant defence mechanisms, enhancing the production of secondary metabolites in plants and plant cell cultures [7,12–14]. Chitin and chitosan (an *N*-deacetylated derivative of chitin), constituents of the cell walls of many filamentous fungi, are examples of such elicitors. The physiological and biochemical changes occurring in plants as a result of chitin and chitosan elicitation have been described [15–17]. One of the main changes seems to be a decrease in the size of the stomatal aperture, which reduces fungal access to the inner leaf tissues. Although they do not display any phytotoxic effects when used as plant protection agents, chitin and chitosan have been found to elicit phytoalexin accumulation in pea (*Pisum sativum*) pods, to cause proteinase inhibitors to accumulate in tomato [*Solanum lycopersicum* (formerly *Lycopersicon esculentum*)] and potato (*Solanum tuberosum*) leaves, and to give rise to the synthesis of a β-1,3-glucan, callose, in parsley (*Petroselinium crispum*) [18,19].

In the present study we investigated the usefulness of chitin and chitosan as potential elicitors of the biosynthesis of secondary metabolites in shoots of *R. graveolens* cultivated *in vitro*. The influence of both elicitors on the growth rate was studied. The simple coumarins, linear furanocoumarins, dihydrofuranocoumarins and furoquinolone alkaloids elicited in the presence of chitin and chitosan were isolated, separated and identified.

Materials and methods

R. graveolens in vitro shoot culture

The *R. graveolens* shoot cultures were grown on B_5 liquid medium [20] supplemented with 3 % (w/v) sucrose [5]. Flasks of volume 250 ml, containing 50 ml of medium and the plant biomass, were continuously agitated on a rotating (orbital) shaker at 110 rev./min at amplitude 9. All cultures were maintained for 28 days at 20 ± 2 °C, under a 16 h photoperiod and illumination of 30–35 $\text{mol} \cdot \text{m}^{-2} \cdot \text{s}^{-1}$ or in darkness. All experiments were performed in triplicate.

Elicitors

Chitin and chitosan were purchased from Sigma–Aldrich. Both elicitors were dissolved in warm sterile water to produce 1 % (w/v) solutions. They were added to prepared cultures (50 ml) such as to obtain 0.1 and 0.01 % solutions of both polysaccharides. The addition of elicitors into *Ruta* shoot culture took place on week 4 (6 days before the end of the culture) of the experiment. This time of addition was chosen because of preliminary observations revealing that the most effective elicitation was achieved during that period.

Extraction of secondary metabolites

Cultures (28 days old) were harvested and dried at 50 °C for 24 h. Samples (3 g dry weight) were extracted exhaustively with light petroleum (boiling range 40–60 °C), chloroform and methanol using a Soxhlet apparatus [21]. Chloroform and methanol extracts were collected separately, evaporated at 50 °C, and the material left dissolved in ethanol (equivalent of 3 g dry mass/5 ml). They were stored in the dark at room temperature (20 °C) until required for further experiments.

Sample preparation

Samples were prepared for analysis at room temperature. The filtrate was diluted with 13 ml of methanol/water (10:3, v/v). The solution was shaken twice with hexane to remove chlorophylls, lipids and oils, after which the methanol/water layer was separated. The methanol was then removed from the methanol/water layer. The residual suspension was treated with 3 ml of 35–38 % (v/v) HCl and extracted twice with chloroform (5 ml) to remove the coumarin-rich fraction. A 3 g portion of solid NaOH was dissolved in the remaining aqueous solution, which was then re-extracted with chloroform (2×5 ml) to yield the alkaloid fraction. The coumarin fraction was evaporated under a stream of nitrogen. The residual suspension was then diluted with 1 ml of ethyl acetate and the coumarins fractionated on a silica-gel column to remove any remaining contamination.

Chemicals

Chloroform, light petroleum, ethyl acetate, methanol, HCl and NaOH were obtained from POCH S.A. Deionized water was supplied by a MilliQ water purification system (Millipore). Stock standard solutions of each coumarin compound were prepared in 10 ml of methanol, then diluted to the required concentration of 1 mg/ml.

The reference standards of the coumarin compounds tested, namely umbelliferone (7-hydroxycoumarin), scopoletin (7-hydroxy,6-methoxycoumarin), bergapten (5-methoxypsoralen), psoralen, xanthotoxin (8-methoxypsoralen), 3-acetylcoumarin, esculetin (6,7-dihydroxycoumarin), 7-methoxycoumarin, 4-methylcoumarin, 7,8-dihydroxy-6-methoxycoumarin and coumarin, were purchased from Sigma–Aldrich.

GC and GC–MS analyses

A GC-8000 TOP gas chromatograph (CE Instruments, Milan, Italy) was used together with a fused-silica capillary column containing EC-1 (30 m long $\times$ 0.25 mm internal diameter; 0.25 μm film thickness) equipped with a flame-ionization detector. In each case a 1 μl sample was injected in splitless mode. The operating conditions were as follows. The initial GC column temperature was 100 °C and, after injection, the temperature was held at 100 °C for 5 min. The temperature

© 2008 Portland Press Ltd

was programmed to rise at 4 °C/min to 320 °C. The injector and detector temperatures were both at 320 °C.

Individual compounds were identified from their EI (electron impact)–MS patterns and compared with the data in the literature. EI–MS analyses were done on a Trio-3 mass spectrometer (VG Masslab Ltd, Altrincham, Cheshire, U.K.). The samples were introduced through a Hewlett–Packard 5890 gas chromatograph equipped with a BP-1 capillary column (30 m long × 0.25 mm internal diameter; 0.25 μm film thickness; Alltech Poland, Warsaw, Poland) with the temperature programmed to rise 4 °C/min from 100 to 320 °C, at which temperature it was held for 5 min. Analyses were performed in triplicate.

All compounds analysed were identified from their EI mass spectra. The following coumarins were identified by their m/z values: rutacultin, 274 $[M]^+$ (74 %), 259 $[M-Me]^+$ (100 %), 231 $[M-CH_3CO]^+$ (58 %) and 219 $[M-55\ u]^+$ (95 %); pinnarin: 274 $[M]^+$ (74 %), 259 $[M-Me]^+$ (100 %), 231 $[M-CH_3CO]^+$ (58 %) and 219 $[M-55\ u]^+$ (95 %); rutamarin: 356 $[M]^+$ (11 %), 341 $[M-Me]^+$, (7 %), 313 (9 %) $[M-43\ u]^+$, 281 $[M-aryl]^+$ (100 %) and 296 (23 %); chalepin: 314 $[M]^+$(94 %), 299 $[M-Me]^+$ (100 %), 255 $[M-OHC^{\bullet}(CH_3)_2]^+$ (53 %) and 281 (36 %); psoralen: 186 $[M]^+$ (100 %), 158 $[M-CO]^+$ (65 %), 130 $[M-CO-CO]^+$ (19 %) and 102 $[M-CO-CO-CO]^+$ (32 %); xanthotoxin: 216 $[M]^+$ (100 %), 201 $[M-Me]^+$ (29 %), 188 $[M-CO]^+$ (14 %), 173 $[M-Me-CO]^+$ (54 %), 145 $[M-Me-CO-CO]^+$ (21 %), 89 (26 %) and 63 (15 %); bergapten: 216 $[M]^+$ (100 %), 201 $[M-Me]^+$ (29 %), 188 $[M-CO]^+$ (14 %), 173 $[M-Me-CO]^+$ (54 %), 145 $[M-Me-CO-CO]^+$ (21 %) and 89 (26 %); isopimpinelin: 246 $[M]^+$ (79 %), 231 $[M-Me]^+$ (100 %), 203 $[M-Me-CO]^+$ (19 %), 188 (28 %), 175 (26 %), 147 (11 %) and 89 (25 %). In addition, four alkaloids were identified by EI–MS: dictamnine: 199 $[M]^+$ (100 %), 184 $[M-Me]^+$ (41 %), 156 (32 %), 128 (19 %); γ-fagarine: 229 $[M]^+$ (100 %), 214 $[M-Me]^+$ (36 %), 200 $[M-CHO^{\bullet}]^+$ (91 %), 156 (25 %), 128 (10 %); skimmianine: 259 $[M]^+$ (51 %), 244 $[M-Me^+]$ (100 %), 230 (68 %); kokusaginine: 259 $[M]^+$ (100 %), 244 $[M-Me]^+$ (11 %), 230 $[M-CHO^{\bullet}]^+$ (63 %), 216 (27 %), 201 (23 %), 173 (18 %).

Results and discussion

Effect of elicitors on the shoots growth of *R. graveolens*

The influence of selected elicitors on growth rates of *R. graveolens* was determined. The elicitors, namely chitin and chitosan, were added at two concentrations (0.01 % and 0.1 %) directly to the medium used for *R. graveolens* culture. Under all tested conditions, significant increases in *R. graveolens* shoot growth rate were observed (Figure 1). The strongest effect was observed when chitin was added

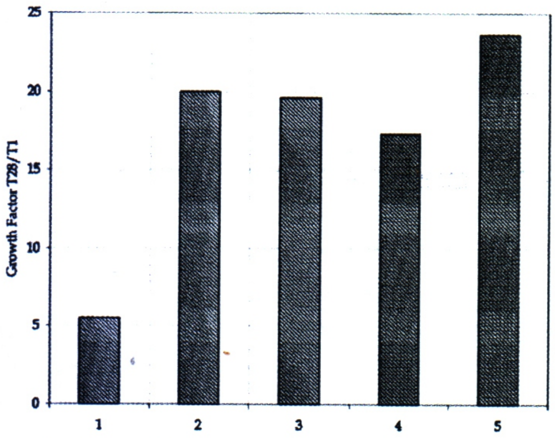

Figure 1 Influence of elicitors on the growth of *R. graveolens* shoot cultures

Key to columns: 1, control without elicitation; 2, 0.01 % chitosan; 3, 0.1 % chitosan; 4, 0.01 % chitin; 5, 0.1 % chitin; with growth factors: 5.5 ± 0.5, 20.0 ± 3.0, 19.5 ± 3.0, 17.3 ± 2.3 and 23.7 ± 3.2 for 1, 2, 3, 4 and 5 respectively.

at a concentration of 0.1 % in the culture medium. In this case, growth increased over 4-fold in comparison with non-elicited tissues. In the case of 0.01 % chitin solution, growth was also enhanced, but to a lower extent. In the case of chitosan, there was hardly any difference between the concentrations used (0.01 and 0.1 %): in both cases growth increased 4-fold in comparison with non-elicited tissue.

Elicitation of coumarins

The bioaccumulation of elicited coumarins by *R. graveolens* shoots in the presence of chitin and chitosan are shown in Table 1. The experiments were performed using two different concentrations (0.1 and 0.01 %) of chitin or chitosan in the media. The results show that application of both compounds resulted in a significant increase in the concentration of nearly all coumarins in *R. graveolens* shoot cultures.

In the case of furanocoumarins, such as bergapten, xanthotoxin or isopimpinelin, the application of the 0.1 % chitosan medium solution doubled production in comparison with the 0.01 % solution. Psoralen and chalepin were not detected in the control samples, but, as a result of chitosan elicitation, the biosynthesis of these coumarins in *R. graveolens* shoots was observed.

Chitin, too, proved to be a very effective elicitor for the production of coumarins by *R. graveolens* shoots. It is known to stimulate the accumulation of proteinase inhibitors in plants and to induce an early cellular response by stimulating the biosynthesis of pathogen-related proteins [23]. The application of 0.01 % of this polysaccharide in the culture medium doubled the production of bergapten and rutacultin, quadrupled the production of pinnarin, and increased the production of xanthotoxin and isopimpinelin 5- and 9-fold respectively as compared with the control value. In the case

© 2008 Portland Press Ltd

Table 1 Influence of elicitors on biosynthesis of coumarins by *R. graveolens*

Abbreviation: n.a., not analysed (below detection limit).

	Concentration of secondary metabolites (μg/g dry wt)				
		Chitosan		Chitin	
Compound	Control	0.01%	0.1%	0.01%	0.1%
Simple coumarins					
Pinnarin	28.0±6	88.7±17	89.4±19	116.7±18	68.2±13
Rutacultin	136.0±21	208.1±37	207.9±35	287.0±48	155.5±31
Furanocoumarins					
Bergapten	412.0±84	333.1±64	772.0±140	904.3±187	514.1±106
Isopimpinelin	56.0±9	182.3±37	470.5±94	490.0±92	167.4±37
Psoralen	n.a.	100.0±21	81.7±16	522.2±131	226.8±54
Xanthotoxin	338.0±72	618.2±112	1200.8±220	1531.5±286	763.8±132
Dihydrofuranocoumarins					
Chalepin	n.a.	n.a.	18.0±3	n.a.	70.0±19
Rutamarin	224.0±46	199.0±42	190.0±38	133.7±35	86.1±12

of psoralen, which was not detected in the control sample, the presence of 0.01% chitin induced accumulation by *R. graveolens* of this coumarin up to 522.2 μg/g dry wt. Such an effective increase in the production of nearly all coumarins suggests that these compounds may be participating in the natural resistance mechanisms of *R. graveolens*. However, a higher concentration of chitin (0.1%) is not as effective as a lower one (0.01%). In the applied concentration (0.1%), apart from the above-discussed rutamarin, most of the analysed coumarins are accumulated to a much lesser extent than when a lower concentration (0.01%) is applied, although still above the level of the control sample. This observation suggests that, above 0.01%, chitin loses its elicitation potential and only few compounds are still synthesized at an elevated level. This might be for several reasons, including inhibitory potential of this polysaccharide at higher concentration ranges as well as its sorption potential, which eliminates metabolites from the solution. Nevertheless, when optimizing this elicitation tool, one has to be aware of this limitation of the concentration range of the chitin applied.

Interestingly, chalepin, which was not detected either in plant samples elicitated by 0.01% chitosan or in the samples grown in the presence of 0.01% of chitin, begins to be accumulate up to 70 μg/g dry wt in the *R. graveolens* shoots elicitated with 0.1% chitin.

On the other hand, in the case of both simple coumarins, namely rutacultin and pinnarin, there was practically no difference in the concentrations of the accumulated compounds in the presence of either concentration of chitosan. With chitosan, regardless of concentration (0.01 or 0.1%), the level of this dihydrofuranocoumarin decreased only slightly. However, when chitin was used as elicitor, the concentration of rutamarin decreased over 2.5-fold in comparison with non-elicited plants. Moreover, there was a slight decrease in the accumulation of bergapten when 0.01% of chitosan was used.

Chitosan, a natural polycationic polymer, is a constituent of the cell walls of various fungi, such as species of the genera *Mucor*, *Absidia*, *Rhizopus* and *Gongronella* of the class Zygomycetes [24]. Several studies have indicated that chitosan's presence induces a natural defence response in many plants [15,24]. Elicitation of a cellular suspension of the brassicaceous flowering plant *Farsetia aegyptia* with chitosan was found to induce the biosynthesis of various phytoalexins [25]. It was also found that chitosan effectively elicits the bioaccumulation of alkaloids, naphthoquinones, phenylpropanoids and terpenoids in the callus and hairy-root cultures [26,27]. Chitin, a polysaccharide component of the cell walls of fungi as well as of arthropod cuticles [24], is known to stimulate the accumulation of proteinase inhibitors in plants and to induce early cellular response by biosynthesis of pathogen-related proteins in the pea, potato and tomato [28].

So far, biotic elicitors have been successfully used to induce the synthesis of coumarin compounds. For example, the lysate from the bacterium *Enterobacter sakazaki* stimulated an increase in the bioaccumulation of umbelliferone, scopoletin and bergapten in cultures of *Ammi majus* (bishop's weed) [29] and *A. majus* hairy-root-culture exudate elicits production of xanthotoxin in *R. graveolens* shoot cultures [22]. Salicylic acid, an abiotic elicitor, was also used as an effective elicitor of umbelliferone and herniatin synthesis in leaves of *Matricaria chamomilla* L. (German chamomile) [30].

Elicitation of alkaloids

The results of the elicitation of alkaloids bioaccumulated by *R. graveolens* shoots in the presence of chitin and chitosan

© 2008 Portland Press Ltd

Table 2 Influence of elicitors on biosynthesis of alkaloids by *R. graveolens*

Abbreviation: n.a., not analysed (below detection limit).

	Concentration of secondary metabolites (μg/g dry wt)				
		Chitosan		Chitin	
Compound	Control	0.01%	0.1%	0.01%	0.1%
Dictamnine	n.a.	n.a.	n.a.	10.4 ± 4	14.5 ± 5
γ-Fagarine	4.9 ± 2	31.1 ± 7	51.8 ± 9	126.5 ± 32	104.7 ± 31
Skimmianine	4.1 ± 2	58.3 ± 9	125.9 ± 21	147.1 ± 40	97.5 ± 26
Ko kusaginine	5.3 ± 3	19.0 ± 7	30.5 ± 8	44.1 ± 8	43.8 ± 9

are presented in Table 2. The experiment was performed using two different concentrations (0.1 and 0.01 %) of chitin or chitosan in the medium. The results show that the application of either compound resulted in a significant increase in the accumulation of nearly all alkaloids by *R. graveolens*. Only in the case of dictamnine did the presence of chitosan not induce biosynthesis of this alkaloid. Application of 0.01 % chitosan did, however, effectively induce the accumulation of methoxylated dictamnine derivatives, i.e., kokusaginine (up to 19.0 μg/g dry wt.), γ-fagarine (up to 31.1 μg/g dry wt.) and skimmianine (up to 58.3 μg/g dry wt.), amounts representing 4-, 6- and 14-fold greater concentrations respectively. The higher concentration of chitosan induced a 10-fold greater production of γ-fagarine, an over 30-fold greater production of skimmianine and a 4-fold more intensive biosynthesis of kokusaginine when compared with the control sample.

The results show that elicitation with chitin is even more effective. Biosynthesis of dictamnine is in this instance initiated and, in the case of this alkaloid, the higher the elicitor concentration, the greater is the accumulation rate. As far as the other elicitors are concerned, the situation resembles the elicitation of coumarins: a lower concentration of chitin is a more effective elicitor of alkaloids than a higher one. The presence of 0.01 % of chitin increases the production of kokusaginine 9-fold, that of skimmianine 25-fold and that of γ-fagarine 36-fold.

Chitosan and its oligomers have been found to be some of the most effective elicitors of secondary metabolites, such as alkaloids, in many plant species [14,31]. It has been effectively used to synthesize menthol in a suspended culture of *Mentha piperita* (peppermint) [26]. When applied at a concentration of 0.02 %, it significantly increased the production of anthraquinone and indirubin in a culture of *Polygonum tinctorum* (dyer's knotweed or Chinese indigo) [32]. It was also used for the elicitation of taxanes by suspended cell cultures of *Taxus chinensis* (Chinese yew) [33,34]. Chitosan has also been found to elevate the biosynthesis of anthracene by *Rheum palmatum* L. (Chinese rhubarb) [35].

Conclusion

In the present study we have demonstrated the enhancement of the growth rate of *R. graveolens* shoots severalfold in all chitosan- or chitin-treated plants; the most potent effect (4-fold growth) being obtained with 0.1 % chitin in the culture medium. Additionally, this is the first report, to our knowledge, of successful application of chitin and chitosan as elicitors for the biosynthesis of simple coumarins, linear furanocoumarins, dihydrofuranocoumarins and furoquinolone alkaloids. The results show that the application of both compounds also significantly increased the concentrations of nearly all coumarins and alkaloids in *R. graveolens* shoot cultures. In the case of furanocoumarins, 0.1 % chitosan doubled the production of secondary metabolites when compared with a concentration of 0.01 %. However, 0.01 % chitin in the culture medium increased coumarin production 2–9-fold in comparison with the control. Such a dramatic increase in the production of nearly all metabolites suggests that these compounds may be participating in the natural resistance mechanisms of *Ruta graveolens* L. The application of the chitin and chitosan in growth media should be considered a promising prospect in the biotechnological production of xanthotoxin, isopimpinelin, psoralen, chalepin or methoxylated dictamnine derivatives.

Acknowledgment

Financial support was provided by the Polish Ministry of Research and Higher Education under grant no. DS 8200-4-0085-7.

References

1 Bohuslavizki, K. H., Hansel, W., Kneip, A., Koppenhofer, E., Niemoller, E. and Sahmann, K. (1994) Gen. Physiol. Biophys. 13, 309–328

© 2008 Portland Press Ltd

2 Zobel, A. M. and Brown, S. A. (1990) J. Chem. Ecol. **16**, 693–700
3 Wolters, B. and Eilert, U. (1981) Planta Med. **43**, 166–174
4 Ekiert, H., Chołoniewska, M. and Gomółka, E. (2001) Biotechnol. Lett. **23**, 543–545
5 Massot, B., Milesi, S., Gontier, E., Bourgaud, F. and Guckert, A. (2000) Plant Cell Tiss. Organ Cult. **62**, 11–19
6 Milesi, S., Massot, B., Gontier, E., Bourgaud, F. and Guckert, A. (2001) Plant Sci., **161**, 189–199
7 Eilert, U. (1987) in Cell Culture and Somatic Genetics of Plants (Constabel, F. and Vasil, I.K., eds.), pp. 153–196, Academic Press, New York
8 Funk, C., Gugler, K. and Brodelius, P. (1987) Phytochemistry **26**, 401–405
9 Tani, M., Fukui, H., Shimomura, M. and Tabata, M. (1992) Phytochemistry **31**, 2719–2723
10 Payne, G.F., Bringi, V., Prince, C.L. and Shuler, M.L. (1991) Plant Cell and Tissue Culture in Liquid Systems, Hanser Publishers, Munich
11 Bhagwath, S.G. and Hjortso, M.A. (2000) J. Biotechnol. **23**, 159–167
12 Cline, S. D. and Coscia, C. J. (1988) Plant Physiol. **86**, 161–165
13 Darvill, A. G. and Albersheim, P. (1984) Annu. Rev. Plant Physiol. **35**, 243–275
14 Funk, C. and Brodelius, P. (1990) Phytochemistry **29**, 845–848
15 Lee, S., Choi, S., Suh, S., Doo, I.-S., Oh, K.-Y., Choi, E. J., Schroeder, A. T., Low, T. P. S. and Lee, Y. (1999) Plant Physiol. **121**, 147–152
16 Lesney, M. S. (1990) Phytochemistry **29**, 1123–1125
17 Vander P. V., Rum, K. M., Domard, A., Eddine, E., Gueddari, N. and Moerschbacher, B. M. (1998) Plant Physiol. **118**, 1353–1359
18 Côté, F. and Hahn, M. G. (1994) Plant Mol. Biol. **26**, 1379–1411
19 Ebel, J. and Mithofer, A. (1998) Planta **206**, 335–348
20 Gamborg, O. L., Miller, R. A. and Ojima, K. (1968) Exp. Cell Res. **50**, 151–158
21 Kamiński, M., Kartanowicz, R., Kamiński, M. M., Królicka, A., Sidwa-Gorycka, M., Łojkowska, E. and Gorzeń, W. (2003) J. Sep. Sci. **26**, 1287–1291
22 Sidwa-Gorycka, M., Krolicka, A., Kozyra, M., Głowniak, K., Bourgaud, F. and Lojkowska, E. (2003) Plant Sci. **165**, 1315–1319
23 Tsigos, I., Martinou, A., Kafetzopoulos, D. and Bouriotis, V. (2000) Trends Biotechnol. **18**, 305–312
24 Rabea, E. I., Bodawy, M. E. T., Stevens, C. V., Smagghe, G. and Steurbaut, W. (2003) Biomacromolecules **4**, 1457–1465
25 Al-Gendy, A. A. and Lockwood, G. B. (2005) Fitoterapia **76**, 288–295
26 Chang, J. H., Shin, J. H., Chung, I. S. and Lee, H. J. (1998) Biotech. Lett. **20**, 1097–1099
27 Komaraiah, P., Amrutha, R. N., Kavi Kishor, P. B. and Rhamakrishna, S. V. (2003) J. Biotechnol. **6**, 181–187
28 Lee, S. C., Kim, Y. J. and Hwang, B. K. (2001) Plant Cell Physiol. **42**, 1321–1330
29 Staniszewska, I., Krolicka, A., Malinski, E., Lojkowska, E. and Szafranek, J. (2003) Enzyme Microb. Technol. **33**, 565–568
30 Eliasova, A., Repcak, M. and Pastirova, A. (2004) Z. Naturforsch. C **59**, 543–548
31 Brodelius, P., Funk, C., Häner, A. and Villegas, M. (1989) Phytochemistry **28**, 2651–2654
32 Kim, J. H., Shin, J. H., Lee, H. J., Chung, I. S. and Lee, H. J. (1997) J. Ferment. Bioeng. **83**, 206–208
33 Zhang, C. H., Mei, X. G., Liu, L. and Yu, L. J. (2000) Biotechnol. Lett. **22**, 1561–1564
34 Zhang, C. H. and Xu, H. B. (2001) Biotechnol. Lett. **23**, 189–193
35 Kasparova, M. and Siatka, T. (2001) Ceska Slov. Farm. **50**, 249–253

Received 20 September 2007/21 December 2007; accepted 22 January 2008
Published as Immediate Publication 22 January 2008, doi:10.1042/BA20070200

© 2008 Portland Press Ltd
